# Biofluid Biomarkers of Cognitive Functioning in Bipolar Disorder: A Systematic Review by the Targeting Cognition and Older‐Age Bipolar Disorder ISBD Task Forces

**DOI:** 10.1111/bdi.70109

**Published:** 2026-07-01

**Authors:** Alexandra J. M. Beunders, Sigfried N. T. M. Schouws, Andrew T. Olagunju, Vicent Balanzá‐Martínez, Nicole C. M. Korten, Ralph W. Kupka, Katherine E. Burdick, Andre F. Carvalho, Ariel G. Gildengers, Lars V. Kessing, Roger S. McIntyre, Paula V. Nunes, Ayal Schaffer, Ivan J. Torres, Shang‐Ying Tsai, Tamsyn E. Van Rheenen, Eduard Vieta, Lakshmi N. Yatham, Allan H. Young, Lisa T. Eyler, Kamilla W. Miskowiak, Annemiek Dols

**Affiliations:** ^1^ GGZ inGeest, Specialized Mental Health Care Amsterdam the Netherlands; ^2^ Amsterdam Public Health Research Institute Amsterdam UMC, location VUmc Amsterdam the Netherlands; ^3^ Department of Psychiatry and Behavioural Neurosciences McMaster University/St. Joseph's Healthcare Hamilton Ontario Canada; ^4^ Department of Medicine Universitat de València, Hospital Clínic Universitari de València, INCLIVA, CIBERSAM Valencia Spain; ^5^ Department of Medical Psychology Northwest Clinics Alkmaar the Netherlands; ^6^ Department of Psychiatry, Brigham and Women's Hospital Harvard Medical School Boston Massachusetts USA; ^7^ IMPACT (Innovation in Mental and Physical Health and Clinical Treatment), strategic Research Centre, School of Medicine, Barwon Health Deakin University Geelong Victoria Australia; ^8^ Department of Psychiatry University of Pittsburgh Pittsburgh Pennsylvania USA; ^9^ Copenhagen Affective Disorder Research Center (CADIC), Psychiatric Center Copenhagen Denmark; ^10^ Department of Clinical Medicine University of Copenhagen Copenhagen Denmark; ^11^ Department of Psychiatry Department of Pharmacology and Toxicology University of Toronto Toronto Ontario Canada; ^12^ Bipolar Disorder Program (PROMAN), Department of Psychiatry University of São Paulo Medical School Sao Paulo Brazil; ^13^ University of British Columbia Vancouver British Columbia Canada; ^14^ Department of Psychiatry University of British Columbia Vancouver British Columbia Canada; ^15^ British Columbia Mental Health and Substance Use Services Vancouver British Columbia Canada; ^16^ Department of Psychiatry Taipei Medical University and Hospital Taipei Taiwan; ^17^ Department of Psychiatry, Faculty of Medicine Dentistry and Health Sciences University of Melbourne Melbourne Australia; ^18^ Centre for Mental Health and Brain Sciences, School of Health Sciences Swinburne University Melbourne Australia; ^19^ Bipolar and Depressive Disorders Unit, Institute of Neuroscience, Hospital Clinic University of Barcelona, IDIBAPS, CIBERSAM Barcelona Catalonia Spain; ^20^ Department of Psychological Medicine Institue Psychiatry, King's College London London UK; ^21^ Department of Brain Sciences, Faculty of Medicine Imperial College London London UK; ^22^ Department of Psychiatry University of California San Diego San Diego California USA; ^23^ Desert‐Pacific Mental Illness Research Education and Clinical Center VA San Diego Healthcare System San Diego California USA; ^24^ Neurocognition and Emotion in Affective Disorders (NEAD) Centre, Department of Psychology University of Copenhagen, and Mental Health Services, Capital Region of Denmark Copenhagen Denmark; ^25^ Neuroscience Research Institute Amsterdam UMC location VUmc Amsterdam the Netherlands; ^26^ Department of Psychiatry, UMC Utrecht Brain Center University Medical Center Utrecht Utrecht the Netherlands

**Keywords:** biomarker, bipolar disorder, cognition, cognitive impairment, elderly, older age bipolar disorder, systematic review

## Abstract

**Background:**

Cognitive impairment is common in bipolar disorder (BD), but the underlying pathophysiology remains unclear. This systematic review aimed to (1) summarize all literature describing relationships of biofluid biomarkers and cognition in BD and (2) identify which biofluid biomarkers correlate most consistently with cognition in BD.

**Methods:**

This systematic review followed procedures of the PRISMA statement. PubMed, EMBASE, and PsycINFO were searched from inception until July 2023. Original studies assessing the relationships between biofluid biomarkers and cognitive functioning in adults with BD were included. Studies on neuroimaging markers and genetic biomarkers were excluded.

**Results:**

We identified 60 studies, together describing 184 biofluid biomarkers that were measured in relation to cognitive functioning in BD. Biomarkers were organized into ten categories: oxidative stress markers (*n* = 14); growth factors (*n* = 13); neurotransmitters (*n* = 14); neuropeptides and hormones (*n* = 14); neurodegenerative markers (*n* = 11); inflammatory/immune markers (*n* = 59); serostatus to infectious agents (*n* = 11); amino acids, vitamins, and minerals (*n* = 7); metabolic factors (*n* = 23); hemogram, coagulation, and fibrinolysis markers (*n* = 18). Preliminary evidence for a significant relationship with cognition appeared for HSV‐1 IgG, CRP, and homocysteine (Hcy); higher biomarker levels were associated with worse cognition.

**Discussion:**

Included studies were heterogeneous and many were deemed to be of low quality following risk of bias assessment. The identified three biofluid biomarkers represent history of previous infections, current inflammation, and/or physical or psychological stress. Poor physical health, possibly represented by a broad range of biomarker aberrations, may play a role in the pathophysiology of cognitive impairment in BD.

**Trial Registration:**

PROSPERO registration number: CRD42021224226

## 1 Introduction

Cognitive impairment is common in bipolar disorder (BD) [1]. Cognition is affected not only during periods of depression or (hypo)mania but also during the euthymic phase in up to 50% of individuals with BD. The observed cognitive impairment is broad; domains of attention, verbal memory, processing speed, executive functions, and social cognition seem affected [2]. Cognition can be affected already after the first episode [3]. Among BD patients, significant cognitive heterogeneity is present. Recent studies suggest three meaningful cognitive subgroups, comprising a *cognitively spared* subtype with “near‐normal” cognition, an *intermediate impairment* subtype, and a *deficit* subtype with impaired performance across all domains [4].

Cognitive impairment in BD occurs in patients of all ages, but is particularly prominent among individuals with older adult bipolar disorder (OABD) [5, 6]. Also, BD is associated with an increased risk of dementia [7]. Importantly, cognitive impairment negatively influences personal, functional, occupational, and social functioning [8, 9]. To date, the pathophysiology of BD‐related cognitive impairment is unclear. An improved understanding of this pathophysiology is of utmost importance to identify patients at risk at an early stage and provide them with adequate monitoring and personalized treatment.

Many correlates of cognitive impairment in BD have been identified [10]. Psychiatric factors such as the number of manic episodes [11], childhood trauma [12], psychotic features [13], psychiatric hospitalizations [14], and use of psychotropic medication [14, 15] were associated with cognitive impairment, indicating that illness severity plays a role. In addition, lifestyle factors such as smoking, hypertension, poor nutrition, substance/alcohol abuse, impaired sleep, physical inactivity, and obesity [16, 17] (partly caused by select psychotropic agents [18]) seem to negatively impact cognition in BD patients. Researchers have also tried to identify biological correlates (biomarkers) of cognitive functioning in BD in order to increase knowledge on the underlying pathophysiology [10, 19]. For this review, we use a broad definition of a biomarker, according to the Biomarkers, EndpointS and other Tools (BEST) glossary definition: “A defined characteristic that is measured as an indicator of normal biological processes, pathogenic processes, or responses to an exposure or intervention, including therapeutic interventions” [20].

Many biomarkers have previously been examined with respect to cognition in BD. These can roughly be divided into three groups: neuroimaging markers, genetic markers, and markers derived from bodily fluids (i.e., serum, plasma, urine, cerebrospinal fluid [CSF], saliva), hereafter referred to as biofluid biomarkers [21]. Other types of markers associated with cognition in BD are arguably not directly biological, such as digital markers [22]. Several reviews have appeared on neuroimaging markers [19, 23] and specific genetic polymorphisms [24, 25] in relation to cognition in BD. Regarding biofluid biomarkers, previous reviews focused on a specific biomarker or biomarker category, for example, “brain‐derived neurotrophic factor (BDNF) and cognition in BD” [26] or “inflammatory biomarkers and cognition in BD” [27, 28, 29], but to date, there has been no systematic review of the *complete* spectrum of biofluid biomarkers in relation to cognition in BD. This is despite a variety of biofluid biomarkers having been examined in the literature, including hormones, vitamins, cytokines, growth factors, neurodegenerative markers, and serostatus to infectious agents.

The aim of the current systematic review by the Targeting Cognition and Older‐Age Bipolar Disorder Task Forces of the International Society for Bipolar Disorders (ISBD) was to summarize and synthesize all literature assessing relationships between biofluid biomarkers and cognitive function in BD patients, irrespective of the function or type of biomarker. Our main focus was to delineate the most consistent cognitive correlates of biofluid biomarkers that are relatively practicable and inexpensive to assay, given these types of biofluid biomarkers have the most potential for use in clinical practice in future [30].

## 2 Methods

### 2.1 Search Strategy

Before commencing the review, the review protocol was registered on PROSPERO on the 13th of August, 2021 (CRD42021224226). This systematic review was conducted according to the updated 2020 Preferred Reporting Items for Systematic Reviews and Meta‐analysis (PRISMA) guidelines [31]. Searches were conducted on PubMed/MEDLINE, EMBASE, and PsycINFO from inception until July 4, 2023. For each database, the search strategy combined terms relating to or describing the domain (bipolar disorder), determinant (biomarker) and outcome (cognitive functioning). Keywords were (“bipolar” OR “manic” OR “mania”) AND (“biomarker” OR “marker”) AND (“cognition” OR “cognitive” OR “neuropsychological”) and all imaginable synonyms of these terms, adapted to each database. Both subject heading terms (MeSH/EMTREE) and free text terms were used (see Appendix S1 for the complete search profile). Duplicate articles were removed automatically using EndNote and manually using Rayyan (www.rayyan.ai). A manual revision of duplicates to be deleted was performed for verification.

Primary title/abstract screening (Appendix S2) and secondary full text screening, (Appendix S3) were performed in duplicate by at least two authors independently using Rayyan. For the primary screening, one author (AJMB) screened all abstracts and five other authors (ATO, AD, SNTMS, VBM, NCMK) each screened a proportion of all abstracts as a second author. Conflicts were discussed and consensus was reached in all cases. In case of doubt during primary screening, the abstract was included for full‐text screening. For the secondary screening, one author (AJMB) screened all full‐texts and three other authors (AD, SNTMS, VBM) each screened a proportion of all full‐texts as a second author. When access to full‐text articles was not available, the corresponding author was contacted once. If full text remained inaccessible, the study was excluded. Agreement between the first and second screener was high (primary screening: 96.7%; secondary screening: 84.6%). Reference lists of all eligible articles were screened manually for additional studies. In order to identify studies that did not mention “(bio)marker” in the abstract but a specific compound name instead, additional snowball searches in PubMed and Google Scholar were performed, e.g., (“bipolar” AND “glucose” AND “cognition”), (“bipolar” AND “triglycerides” AND “cognition”).

### 2.2 Screening Strategy

During primary screening, we selected records that fulfilled the following inclusion criteria: (1) original, published, peer‐reviewed research articles; (2) human subjects aged ≥ 18 years meeting diagnostic criteria for BD; (2) measurement of at least one biomarker in a bodily fluid; (3) investigation of an objective cognitive measure (neuropsychological test or cognitive composite score) OR comparison of separate diagnostic cognitive BD groups (e.g., BD‐no cognitive impairment vs. BD‐MCI vs. BD‐dementia). Although many of the studies included a healthy control (HC) group, a BD versus HC comparison on biomarkers or cognitive measures was not the focus of this review (Appendix S2). Rather, we were interested in studies assessing the relationship between a biofluid biomarker and a cognitive outcome *within* the BD population. During secondary screening, we therefore also thoroughly checked if the study assessed and reported on a *direct* relationship between a biomarker and cognitive outcome (Appendix S3). As we aimed to focus on biomarkers that are naturally present in the human body, we only included an RCT if there was a baseline evaluation of an association between biomarker and cognition.

### 2.3 Eligibility Criteria

We excluded studies that: (1) did not include peer‐reviewed original research data in humans, such as vitro/animal research, conference or poster abstracts, letters, editorials, opinions, reviews, dissertations; (2) were case reports describing < 5 individuals with BD; (3) included combined samples with several diagnoses (unless data for BD was reported separately); (4) included samples with individuals aged below 18 years (unless data for adults were reported separately); (5) combined analyses for a mixed group of BD and HC (unless data for BD was reported separately); (6) *only* measured structural or functional imaging biomarkers (e.g., neuroanatomical markers, fMRI, CT, PET, SPECT, EEG, measurement of cerebral blood flow, diffusion tensor imaging); (7) *only* measured genetic markers (e.g., polymorphisms of genes (such as APO‐E or BDNF alleles), candidate genes, chromosome regions (GWAS studies), circulating mRNA/DNA in blood, or other genetic analyses); (8) did not clearly describe or define the cognitive outcome; (9) *only* measured IQ, general intellectual ability, or a cognitive screening instrument (e.g., MMSE, MoCA, FAB), as these have lower discriminative power and may not reflect the real cognitive performance; (10) *only* used subjective assessments of cognition (either self‐report or expert opinion from clinicians, rather than objective cognitive testing); (11) *only* compared a biomarker and/or cognitive measure in BD versus healthy controls; (12) did not assess or report on a (statistical) relationship between a biofluid biomarker and cognitive outcome for a BD group. We excluded articles that focused on the relationship between specific diseases or health conditions and cognition in BD; for example, we considered triiodothyronine (T3), insulin, and glucose as biofluid biomarkers, but excluded studies on hyper‐ or hypothyroidism, insulin resistance, and diabetes in relation to cognition. See Appendix S2 and S3 for the detailed inclusion and exclusion criteria per screening stage.

### 2.4 Data Extraction

Data extraction was performed in parallel by two authors (AB, SNTMS) using an a priori designed, standardized data extraction spreadsheet in Microsoft Excel. Extracted information included: study design, study population and sample size, participant demographics (age, gender, education level, illness duration, use of psychotropic medication), type of biomarker and biofluid, neuropsychological assessment (tests and domains), criteria used for MCI or dementia diagnosis, statistical methods, effect sizes, standard errors, covariates, and main findings. In case articles shared study samples, we evaluated if different biomarkers were reported. If multiple articles reported the same biomarkers using the same study samples, the most comprehensive source was used.

### 2.5 Methodological Quality Assessment

The majority of studies that were identified were observational, cross‐sectional studies, but also included two randomized controlled trials (RCTs) [32, 33]. We adapted the “Joanna Briggs Institute Critical Appraisal tool for analytical cross‐sectional studies”, focusing on specific methods for bipolar disorder diagnosis, biomarker measurement, neuropsychological assessment and on the quality of statistical analyses. We added an extra criterion on sample size: a sufficient sample size was defined as at least 100 BD patients. Risk of bias assessment was carried out in parallel by two authors (AB, SNTMS). For each study, eight appraisal questions were evaluated for the presence (“Yes”) or absence (“No”) of good quality. In case of unclear quality due to poor reporting, the specific criteria received a “No”. Ultimately, each study received an overall quality appraisal: “Good”, “Fair”, or “Poor” (see Appendix S4 for the complete adapted criteria).

## 3 Results

### 3.1 Study Selection

**FIGURE 1:**
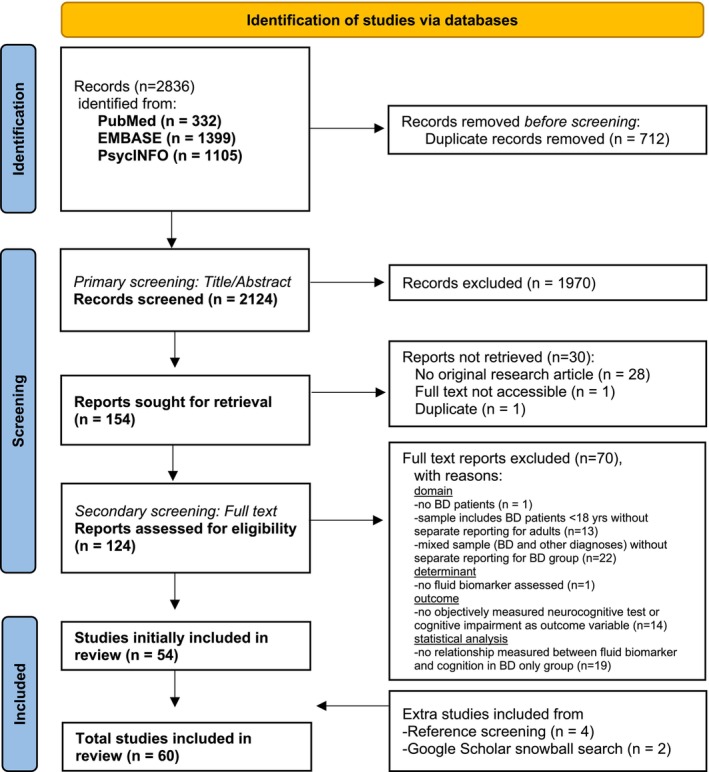
PRISMA 2020 flow diagram for this systematic review. BD, bipolar disorder. Last search performed on July 4th, 2023. Format from: Page et al. [31]. http://www.prisma‐statement.org/.

The computerized search identified 2124 articles (after removal of duplicates), all of which were included for the title/abstract screening (see Figure 1 for Flowchart). Out of 124 articles evaluated with full‐text screening, 70 were excluded for different reasons. The search of reference lists of included articles and snowball searches yielded 6 additional articles. The complete search process resulted in 60 studies that met eligibility criteria and were included in this review (Figure 1).

### 3.2 Study Characteristics

The study characteristics of all 60 studies are listed in Appendix S5, Table S1. Most studies (*n* = 53) performed cross‐sectional analyses. Four studies with a longitudinal design combined cognitive data from multiple timepoints [34, 35, 36, 37]. We included two RCTs (one with infliximab [33] and one with open label valproate [38]), as both of these also investigated the cross‐sectional relationship of a biomarker and cognition at baseline before treatment.

Although one of the inclusion criteria was inclusion of “BD‐only” samples, two studies did include a few patients with schizoaffective disorder bipolar subtype within the BD sample, since the authors of these two studies considered this a subtype of BD [35, 39]. The number of BD patients included in the studies ranged from 8 [40] to 791 [41]; most studies included around 40–80 individuals with BD. Out of 60 studies, 45 also included a healthy control (HC) group. Overall, females were included more often than males. The mean age of BD patients was mostly between 30 and 45 years. Three studies specifically included older patients: [40] included geriatric inpatients (mean age 77.0 years), [42] patients aged ≥ 55 years, and [43] patients aged ≥ 50 years. Almost all studies included patients that used psychotropic medication; exceptions were [44] (*n* = 10 out of 15/67% medication‐free), [45] (*n* = 27 out of 27/100% medication‐free), and two studies by Lee et al. (100% medication‐free at baseline before start of the RCT) [32, 46]. By far, most of the 60 studies in this review included euthymic outpatients using strict inclusion criteria. With regard to the included studies on CRP and Hcy, however, there were also some studies that included inpatients or outpatients with an affective episode or suicidal ideations (see Table 1 and Appendix S5, Table S1) [41, 67, 72, 74, 84, 86].

**TABLE 1:** (a–j). Biofluid biomarkers in relation to cognition in BD.

a. Oxidative stress markers (*n* = 14)						
Biomarker	Biomarker abbreviation	Type of biofluid	Study	Relationship with cognitive functioning	Cognitive outcome	Effect size and standard error/95% CI^a^
			First author (year)	**Positive =** higher biomarker level related to better cognition; **Inverse =** higher biomarker level related to worse cognition; **Non‐significant =** relationship not statistically significant; **Unclear =** unclear direction of effect or data not shown	Reported only for significant relationships	Reported only for significant relationships
Total glutathione	GSH	Serum	Garés‐Caballer et al. [37]	**Positive**	Processing speed (at 1 year FU)	Predictive model with PCR‐us, GSH, and SOD explained 29.9% of variance (*R* ^2^) of processing speed. GSH: *β* = 0.46, 95% CI (0.00–0.01), *p* < 0.05
		Whole blood	Aydemir et al. [47]	Non‐significant	—	—
Superoxide dismutase	SOD	Serum	Aydemir et al. [47]	Non‐significant	—	—
			Garés‐Caballer et al. [37]	**Positive**	Processing speed (at 1 year FU)	Predictive model with PCR‐us, GSH, SOD explained 29.9% of variance (*R* ^2^) of processing speed. SOD: *β* = 0.47, 95% CI (0.00–0.01), *p* < 0.05
				**Positive**	Verbal fluency (at 1 year FU)	Predictive model with IL‐6, PCR‐us, SOD, HGB, HCT explained 49.8% of variance (*R* ^2^) of verbal fluency. SOD: *β* = 0.44, 95% CI (0.002–0.009), *p* < 0.01
Nitric oxide	NO	Serum	Aydemir et al. [47]	Non‐significant	—	—
			Tozoglu et al. [48]	**Inverse**	Stroop 3 duration	*r* = 0.324, *p* = 0.038
Malondialdehyde	MDA	Plasma	Aydemir et al. [47]	Non‐significant	—	—
Asymmetrical dimethylarginine	ADMA	Serum	Tozoglu et al. [48]	**Positive**	RAVLT 4	*r* = 0.298, *p* = 0.011
				**Positive**	RAVLT total	*r* = 0.251, *p* = 0.033
				**Positive**	RAVLT separation ratio correct	*r* = 0.301, *p* = 0.011
Symmetrical dimethylarginine	SDMA	Serum	Tozoglu et al. [48]	**Inverse**	Stroop 1 duration	*r* = 0.384, *p* = 0.001
				**Inverse**	Stroop 2 duration	*r* = 0.390, *p* = 0.001
				**Inverse**	Stroop 5 duration	*r* = 0.415, *p* < 0.001
Total antioxidant capacity	TAC	Serum	Tozoglu et al. [48]	**Positive**	WCST total number correct	*r* = 0.310, *p* = 0.043
Neurotrophin‐4	NT‐4	Serum	Aydemir et al. [47]	Non‐significant	—	—
Reactive oxygen species	ROS	Serum	Garés‐Caballer et al. [37]	Non‐significant	—	—
Mitochondrial reactive oxygen species	mROS	Serum	Garés‐Caballer et al. [37]	Non‐significant	—	—
8‐Dihydroguanosine	8‐oxo‐Guo	CSF	Miskowiak et al. [34]	**Inverse** (non‐significant after Bonferroni correction)	Global cognition	B = −0.137, 98.25% CI (−0.442 to 0.169), *p* = 1 (Bonferroni correction)
				**Inverse** (non‐significant after Bonferroni correction)	Verbal learning and memory	B = −0.265, 98.25% CI (−0.673 to 0.142), *p* = 0.572 (Bonferroni correction)
		Spot urine	Miskowiak et al. [34]	Non‐significant	—	—
8‐Hydroxy‐2‐deoxyguanosine	8‐oxo‐dG	CSF	Miskowiak et al. [34]	Non‐significant	—	—
		Spot urine	Miskowiak et al. [34]	Non‐significant	—	—
Adenine nucleotide translocase 3	ANT3	Plasma	Knöchel et al. [49]	Non‐significant	—	—
HSP70 escort protein 2	HEP2	Plasma	Knöchel et al. [49]	Non‐significant	—	—

b. Growth factors (*n* = 13)						
Biomarker	Biomarker abbreviation	Type of biofluid	Study	Relationship with cognitive functioning	Cognitive outcome	Effect size and standard error/95% CI^a^
			First author (year)	**Positive =** higher biomarker level related to better cognition; **Inverse =** higher biomarker level related to worse cognition; **Non‐significant =** relationship not statistically significant; **Unclear =** unclear direction of effect or data not shown	Reported only for significant relationships	Reported only for significant relationships
Brain‐derived neurotrophic factor	BDNF	Serum	Mora et al. [50]	**Positive**	Executive functioning	*β* = 0.010, SE = 0.004, *p* = 0.020
				**Positive**	Verbal memory	*β* = 0.013, SE = 0.005, *p* = 0.005
		Plasma	Chou et al. [51]	Unclear	Divided attention, subscore Sounds RT	‘significant correlation’, *p* = 0.04
				Unclear	Faces memory, subscore faces 2 true positive	‘significant correlation’, *p* = 0.01
			Liou et al. [41]	**Positive**	Continuous Performance Test—Omission	*r* = −0.11, *p* < 0.05
				**Positive**	Continuous Performance Test—Hit reaction time	*r* = −0.09, *p* < 0.05
				**Positive**	Continuous Performance Test—Hit reaction time standard error	*r* = −0.13, *p* < 0.05
			Strawbridge et al. [52]	Non‐significant	—	—
Erythropoietin/hematopoietin	EPO	CSF	Miskowiak et al. [34]	**Inverse** (non‐significant after Bonferroni correction)	Global Cognition	B = −0.011, 98.25% CI (−0.103 to 0.082), *p* = 1 (Bonferroni correction)
				**Inverse** (non‐significant after Bonferroni correction)	Verbal Learning and Memory	B = −0.03, 98.25% CI (−0.143 to 0.084), *p* = 1 (Bonferroni correction)
Basic fibroblast growth factor	bFGF	plasma	Poletti et al. [53]	**Inverse**	Executive function	Wald = 5.01, B = 0.07, SE = 0.03, *p* = 0.025
			Strawbridge et al. [52]	**Positive**	Global cognitive performance	Multivariable: B = 0.344, 95% CI (0.085 to 0.894), *p* = 0.007
Fibroblast growth factor 21	FGF21	plasma	Omileke et al. [54]	**Positive**	Letter fluency	*r* = 0.42, *p* = 0.034
				**Positive**	Motor speed	*r* = 0.48, *p* = 0.014
Placental growth factor	PlGF	plasma	Strawbridge et al. [52]	**Positive**	Cognitive impairment (dichotomous)	Multivariable: OR = 0.000, 95% CI (0.000–0.265), *p* = 0.023
				**Positive**	Global cognitive performance	Multivariable: B = 0.426, 95% CI (0.298–3.861), *p* = 0.048
fms‐like tyrosine kinase	Flt‐1	plasma	Strawbridge et al. [52]	Non‐significant	—	—
Angiopoietin‐1 receptor	Tie‐2	plasma	Strawbridge et al. [52]	Non‐significant	—	—
Platelet‐derived growth factor with two B subunits	PDGF‐BB	plasma	Poletti et al. [53]	Non‐significant	—	—
Granulocyte‐colony stimulating factor	G‐CSF	plasma	Poletti et al. [53]	Non‐significant	—	—
Granulocyte‐macrophage colony‐stimulating factor	GM‐CSF	plasma	Poletti et al. [53]	Non‐significant	—	—
Vascular endothelial growth factor	VEGF	plasma	Poletti et al. [53]	Non‐significant	—	—
			Strawbridge et al. [52]	Non‐significant	—	—
Vascular endothelial growth factor C	VEGF‐C	plasma	Strawbridge et al. [52]	**Positive**	Cognitive impairment (dichotomous)	Multivariable: OR = 0.022, 95% CI (0.001–0.590), *p* = 0.006
				**Positive**	Global cognitive performance	Multivariable: B = 0.381, 95% CI (0.275–1.815), *p* = 0.006
Vascular endothelial growth factor D	VEGF‐D	plasma	Strawbridge et al. [52]	Non‐significant	—	—

c. Neurotransmitters (*n* = 14)						
Biomarker	Biomarker abbreviation	Type of biofluid	Study	Relationship with cognitive functioning	Cognitive outcome	Effect size and standard error/95% CI^a^
			First author (year)	**Positive =** higher biomarker level related to better cognition; **Inverse =** higher biomarker level related to worse cognition; **Non‐significant =** relationship not statistically significant; **Unclear =** unclear direction of effect or data not shown	Reported only for significant relationships	Reported only for significant relationships
Glutamate	Glu	‘blood samples’	King et al. [44]	Non‐significant	—	—
5‐Hydroxytryptamine (serotonin)	5‐HT	plasma	Paribello et al. [55]	Unclear	—	—
Tryptophan	TRP	plasma	Paribello et al. [55]	Unclear	—	—
		serum	Platzer et al. [56]	Unclear	—	—
5‐Hydroxytryptophan (oxitriptan)	5‐HTP	plasma	Paribello et al. [55]	**Inverse**	BAC‐A: color naming subtask	LSA subgroup: *R* = −0.5, *p* = 0.049. LSI subgroup: *R* = −0.52, *p* = 0.013. Adjusted for illness duration and gender: *p* = 0.002.
Quinolinic acid	QA	plasma	Hebbrecht et al. [35]	Non‐significant	—	—
			Paribello et al. [55]	Unclear	—	—
Kynurenine	KYN	plasma	Paribello et al. [55]	Unclear	—	—
		serum	Platzer et al. [56]	Unclear	—	—
Kynurenic acid	KYNA	plasma	Hebbrecht et al. [35]	**Positive**	Global cognitive functioning	BD‐depression subgroup: *β* = 0.114, *p* = 0.02
				**Positive**	BACS‐SC (processing speed)	BD‐depression subgroup: *β* = 0.139, *p* = 0.03
				**Positive**	BACS‐SC (processing speed)	BD‐mania subgroup: *β* = 0.279, *p* = 0.02
			Paribello et al. [55]	Unclear	—	—
		Serum	Platzer et al. [56]	Unclear	—	—
3‐Hydroxykynurenine	3‐HK	Plasma	Hebbrecht et al. [35]	Non‐significant	—	—
			Paribello et al. [55]	Unclear	—	—
		Serum	Platzer et al. [56]	Unclear	—	—
3‐Hydroxykynurenine/kynurenic acid ratio	3‐HK/KYNA ratio	Plasma	Hebbrecht et al. [35]	Non‐significant	—	—
		Serum	Platzer et al. [56]	**Inverse**	CVLT trial 1–5	Male subgroup: *r* = −0.43, *p* = 0.01
				**Inverse**	CVLT short recall	Male subgroup: *r* = −0.41, *p* = 0.02
				**Inverse**	CVLT long recall	Male subgroup: *r* = −0.37, *p* = 0.04
Kynurenine/3‐hydroxykynurenine ratio	KYN/3‐HK ratio	Serum	Platzer et al. [56]	**Positive**	CVLT trial 1–5	Male subgroup: *r* = 0.43, *p* = 0.01
5‐Hydroxytryptophan/tryptophan ratio	5‐HTP/TRP ratio	Plasma	Paribello et al. [55]	Unclear	—	—
Kynurenine/kynurenic acid ratio	KYN/KYNA ratio	Serum	Platzer et al. [56]	Non‐significant	—	—
Kynurenine/tryptophan*1000 ratio	KYN/TRP*1000 ratio	Plasma	Paribello et al. [55]	Unclear	—	—
Quinolinic acid/kynurenic acid ratio	QA/KYNA ratio	Plasma	Paribello et al. [55]	Unclear	—	—

d. Neuropeptides and hormones (*n* = 14)						
Biomarker	Biomarker abbreviation	Type of biofluid	Study	Relationship with cognitive functioning	Cognitive outcome	Effect size and standard error/95% CI^a^
			First author (year)	**Positive =** higher biomarker level related to better cognition; **Inverse =** higher biomarker level related to worse cognition; **Non‐significant =** relationship not statistically significant; **Unclear =** unclear direction of effect or data not shown	Reported only for significant relationships	Reported only for significant relationships
α‐Melanocyte‐stimulating hormone	α‐MSH	Plasma	Hidese et al. [57]	Non‐significant	—	—
*β*‐Endorphin		Plasma	Hidese et al. [57]	Non‐significant	—	—
Neurotensin		Plasma	Hidese et al. [57]	Non‐significant	—	—
Oxytocin	OT	Plasma	Hidese et al. [57]	Non‐significant	—	—
		Serum	Rubin et al. [58]	Non‐significant	—	—
Substance P		Plasma	Hidese et al. [57]	Non‐significant	—	—
Pentraxin‐3	PTX3	Serum	Tanaka et al. [59]	Non‐significant	—	—
Arginine vasopressin	AVP	Serum	Rubin et al. [58]	Non‐significant	—	—
Cortisol		Serum	Lee et al. [32]	Non‐significant (at baseline)	—	—
			Tournikioti et al. [60]	Non‐significant	—	—
			Tournikioti et al. [61]	**Inverse**	Paired associative learning (PAL) (visuospatial)	*r* = 0.317, *p* = 0.022; Adjusted: *β* = 0.264, *p* = 0.022
		Saliva	Thompson et al. [62]	**Positive**	Stroop colour‐word latency	*r* = −0.33, *p* = 0.015
				**Positive**	Tower of London (TOL): excess moves	*r* = −0.312, *p* = 0.022
				**Positive**	COWAT (verbal fluency)	*r* = 0.303, *p* = 0.025
			van der Werf [36]	Non‐significant	—	—
Transcortin	CBG	Plasma	Knöchel et al. [49]	Non‐significant	—	—
Dehydroepiandrosterone	DHEA	Serum	Lee et al. [46]	Unclear	—	—
Dehydroepiandrosterone sulfate	DHEA‐S	Serum	Lee et al. [46]	**Inverse**	BACA verbal memory	BD‐II subgroup: *F* = 12.67, *p* = 0.001
				**Inverse**	BACA composite score	BD‐II subgroup: *F* = 7.27, *p* = 0.011
			Tournikioti et al. [60]	Non‐significant	—	—
Pregnenolone		Serum	Lee et al. [46]	Unclear	—	—
Cortisol/DHEA‐S ratio		Serum	Tournikioti et al. [60]	**Inverse**	Stockings of Cambridge (SOC)	*β* = −0.318, *p* = 0.015
Triiodothyronine	T3	Serum	Chen et al. [42]	Non‐significant	—	—

e. Neurodegenerative markers (*n* = 11)						
Biomarker	Biomarker abbreviation	Type of biofluid	Study	Relationship with cognitive functioning	Cognitive outcome	Effect size and standard error/95% CI^a^
			First author (year)	**Positive =** higher biomarker level related to better cognition; **Inverse =** higher biomarker level related to worse cognition; **Non‐significant =** relationship not statistically significant; **Unclear =** unclear direction of effect or data not shown	Reported only for significant relationships	Reported only for significant relationships
Neurofilament light chain	NfL	Serum	Chen et al. [63]	**Inverse**	Cognitive composite score	Younger age subgroup: −0.574 (−1.144 to −0.005), *p* = 0.048
				**Inverse**	Verbal fluency	Younger age subgroup: −0.522 (−0.726 to −0.319), *p* < 0.001
				**Inverse**	Processing speed	Younger age subgroup: −0.769 (−1.235 to −0.303), *p* = 0.001
				**Positive**	Verbal fluency	Older age subgroup: 0.238 (0.071–0.404), *p* = 0.005
		CSF	Rolstad et al. [64]	**Inverse**	Memory	*β* = −0.37, *p* < 0.0005
				**Inverse**	Verbal functions	*β* = −0.39, *p* < 0.005
Total Tau	T‐Tau	CSF	Jakobsson et al. [39]	**Positive**	Trail making test visual scanning (TMT‐1)	*r* = 0.302, *p* = 0.005
				**Positive**	Trail making test number sequencing (TMT‐2)	*r* = 0.276, *p* = 0.011
				**Positive**	Trail making test letter sequencing (TMT‐3)	*r* = 0.283, *p* = 0.009
				**Positive**	WAIS‐III working memory	*r* = 0.348, *p* = 0.001
			Rolstad et al. [64]	**Inverse**	Executive functions	*β* = −0.35, *p* < 0.005
				**Positive**	Verbal functions	*β* = 0.14, *p* = 0.03
				**Positive**	Visuospatial functions	*β* = 0.33, *p* = 0.01
Hyperphosphorylated Tau	P‐Tau	CSF	Jakobsson [39]	Non‐significant	—	—
			Rolstad et al. [64]	Non‐significant	—	—
Amyloid beta 1–42	A*β*1‐42	CSF	Jakobsson [39]	**Positive**	Trail making test letter sequencing (TMT‐3)	*r* = 0.241, *p* = 0.027
			Rolstad et al. [64]	Non‐significant	—	—
Amyloid beta 42/Amyloid beta 40 ratio	A*β*42/40 ratio	CSF	Jakobsson [39]	Non‐significant	—	—
			Rolstad et al. [64]	**Inverse**	Visuospatial functions	*β* = −0.52, *p* < 0.005
				**Inverse**	Memory	*β* = −0.30, *p* = 0.01
				**Inverse**	Attention/speed	*β* = −0.07, *p* = 0.01
				**Inverse**	Verbal functions	*β* = −0.18, *p* = 0.04
Amyloid beta 42/Amyloid beta 38 ratio	A*β*42/38 ratio	CSF	Jakobsson [39]	Non‐significant	—	—
			Rolstad et al. [64]	**Positive**	Memory	*β* = 0.27, *p* = 0.01
				**Positive**	Attention/speed	*β* = 0.06, *p* = 0.02
				**Positive**	Visuospatial functions	*β* = 0.48, *p* = 0.04
Amyloid beta 38	A*β*38	CSF	Jakobsson [39]	Non‐significant	—	—
Amyloid beta 40	A*β*40	CSF	Jakobsson [39]	**Positive**	Trail making test letter sequencing (TMT‐3)	*r* = 0.328, *p* = 0.002
Amyloid beta 42	A*β*42	CSF	Jakobsson [39]	Non‐significant	—	—
Soluble amyloid precursor protein alfa	sAPP‐α	CSF	Jakobsson [39]	**Positive**	Trail making test letter sequencing (TMT‐3)	*r* = 0.319, *p* = 0.003
			Rolstad et al. [64]	**Inverse**	Attention/speed	*β* = −0.27, *p* = 0.03
Soluble amyloid precursor protein beta	sAPP‐*β*	CSF	Jakobsson [39]	**Positive**	Trail making test letter sequencing (TMT‐3)	*r* = 0.314, *p* = 0.004

f. Inflammatory/immune markers (*n* = 59)						
Biomarker	Biomarker abbreviation	Type of biofluid	Study	Relationship with cognitive functioning	Cognitive outcome	Effect size and standard error/95% CI^a^
			First author (year)	**Positive =** higher biomarker level related to better cognition; **Inverse =** higher biomarker level related to worse cognition; **Non‐significant =** relationship not statistically significant; **Unclear =** unclear direction of effect or data not shown	Reported only for significant relationships	Reported only for significant relationships
Interleukin‐1α	IL‐1α	Plasma	Strawbridge et al. [52]	Non‐significant	—	—
Interleukin‐1*β*	IL‐1*β*	Plasma	Poletti et al. [53]	**Inverse**	Speed of information processing	“Significantly increased likelihood of poor cognitive performance”
				**Inverse**	Verbal fluency	“Significantly increased likelihood of poor cognitive performance”
Interleukin‐1 receptor antagonist	IL‐1RA	Plasma	Lotrich et al. [43]	**Inverse**	Global cognitive function (21 tests)	B = −0.57, SE = 0.28, *p* = 0.048
			Poletti et al. [53]	Non‐significant	—	—
Interleukin‐2	IL‐2	Plasma	Barbosa et al. [65]	Non‐significant	—	—
			Poletti et al. [53]	Non‐significant	—	—
Interleukin‐4	IL‐4	Plasma	Barbosa et al. [65]	Non‐significant	—	—
			Poletti et al. [53]	Non‐significant	—	—
Interleukin‐5	IL‐5	Plasma	Poletti et al. [53]	Non‐significant	—	—
Interleukin‐6	IL‐6	Plasma	Barbosa et al. [65]	**Inverse**	Total BAC‐A z‐score	B = −0.935, *β* = −0.462, *p* = 0.0004
			Poletti et al. [53]	**Inverse**	Executive function	“Significantly increased likelihood of poor cognitive performance”
			Strawbridge et al. [52]	Non‐significant	—	—
		Serum	Civil Arslan et al. [66]	Non‐significant	—	—
			Mora et al. [50]	Non‐significant	—	—
			Garés‐Caballer et al. [37]	**Positive**	Verbal fluency (at 1 year FU)	Predictive model with IL‐6, PCR‐us, SOD, HGB, HCT explained 49.8% of variance (*R* ^2^) of verbal fluency. IL‐6: *β* = 0.51, 95% CI (0.02 to 0.24), *p* < 0.05
		‘Blood samples’	King et al. [44]	Unclear	—	—
Soluble interleukin‐6 receptor	sIL‐6R	Serum	Huang et al. [67]	Non‐significant	—	—
			Huang et al. [68]	**Inverse**	Word list II recall	BD‐I subgroup: *B* = −2.258, SE = 1.042, *p* = 0.032
Interleukin‐7	IL‐7	Plasma	Poletti et al. [53]	Non‐significant	—	—
			Strawbridge et al. [52]	**Positive**	Cognitive impairment group (dichotomous)	Multivariable: OR = 0.018, 95% CI (0.001–0.369), *p* = 0.005
				**Positive**	Global cognitive performance	Multivariable: *β* = 0.381, 95% CI (0.181–1.322), *p* = 0.003
Interleukin‐8	IL‐8	Plasma	Liou et al. [41]	**Inverse**	WCST ‐ total number of errors	*r* = 0.11, *p* < 0.01
				**Inverse**	WCST ‐ number of completed categories	*r* = −0.13, *p* < 0.01
				**Inverse**	WCST ‐ conceptual level responses	*r* = −0.10, *p* < 0.05
			Poletti et al. [53]	Non‐significant	—	—
			Strawbridge et al. [52]	Non‐significant	—	—
Interleukin‐9	IL‐9	Plasma	Poletti et al. [53]	Non‐significant	—	—
Interleukin‐10/human cytokine synthesis inhibitory factor	IL‐10/CSIF	Plasma	Barbosa et al. [65]	Non‐significant	—	—
			Poletti et al. [53]	Non‐significant	—	—
			Strawbridge et al. [52]	Non‐significant	—	—
		Serum	Mora et al. [50]	Non‐significant	—	—
			Garés‐Caballer et al. [37]	Non‐significant	—	—
Interleukin‐12	IL‐12	Plasma	Poletti et al. [53]	Non‐significant	—	—
			Strawbridge et al. [52]	Non‐significant	—	—
Interleukin‐13	IL‐13	Plasma	Poletti et al. [53]	Non‐significant	—	—
Interleukin‐15	IL‐15	Plasma	Poletti et al. [53]	Non‐significant	—	—
			Strawbridge et al. [52]	Non‐significant	—	—
Interleukin‐16	IL‐16	Plasma	Strawbridge et al. [52]	**Positive**	Global cognitive performance	Univariate: *r* = 0.297, *p* = 0.050
Interleukin‐17A	IL‐17/IL‐17A	Plasma	Barbosa et al. [65]	Non‐significant	—	—
			Poletti et al. [53]	**Positive**	Verbal fluency	“significantly increased likelihood of good cognitive performance”
			Strawbridge et al. [52]	Non‐significant	—	—
Interleukin‐18	IL‐18	Serum	Civil Arslan et al. [66]	**Positive**	WCST nr of completed categories	*r* = 0.288, *p* = 0.05
				**Positive**	WCST perseverative responses	*r* = −0.264, *p* = 0.05
				**Positive**	WCST nr of perseverative errors	*r* = −0.279, *p* = 0.01
				**Positive**	Immediate recall	*r* = 0.307, *p* = 0.01
				**Positive**	Delayed recall	*r* = 0.321, *p* = 0.01
				**Positive**	Learning scores	*r* = 0.297, *p* = 0.05
				**Positive**	Stroop interference	*r* = −0.235, *p* < 0.05
Interferon‐γ	IFN‐γ	Plasma	Barbosa et al. [65]	Non‐significant	—	—
			Poletti et al. [53]	Non‐significant	—	—
			Strawbridge et al. [52]	Non‐significant	—	—
Interferon‐γ‐induced protein‐10	IP‐10/CXCL10	Plasma	Poletti et al. [53]	**Inverse**	Working memory	Wald = 6.29, B = 0.005, SE = 0.002, *p* = 0.012
				**Inverse**	Verbal fluency	Wald = 5.85, B = 0.005, SE = 0.002, *p* = 0.015
				**Inverse**	Psychomotor coordination	Wald = 7.45, B = 0.006, SE = 0.002, *p* = 0.006
				**Inverse**	Speed of information processing	Wald = 4.57, B = 0.006, SE = 0.003, *p* = 0.033
			Strawbridge et al. [52]	Non‐significant	—	—
Tumor necrosis factor‐alfa	TNF‐α	Plasma	Barbosa et al. [65]	Non‐significant	—	—
			Liou et al. [41]	**Inverse**	WCST ‐ total number error	*r* = 0.09, *p* < 0.05
				**Inverse**	WCST ‐ number of completed categories	*r* = −0.10, *p* < 0.05
				**Inverse**	WCST ‐ conceptual level	*r* = −0.09, *p* < 0.05
				**Inverse**	WMS‐III ‐ visual immediate index	*r* = −0.10, *p* < 0.05
			Mansur et al. [33]	Unclear	—	—
			Poletti et al. [53]	Non‐significant	—	—
			Strawbridge et al. [52]	Non‐significant	—	—
			Zazula et al. [69]	Non‐significant	—	—
		Serum	Mora et al. [50]	Non‐significant	—	—
			Millett et al. [70]	**Inverse**	Stroop test	*r* = −0.2, *p* = 0.005
				**Inverse**	WCST	*r* = −0.2, *p* = 0.03
			Garés‐Caballer et al. [37]	Unclear	—	—
		‘Blood samples’	King et al. [44]	Unclear	—	—
Soluble tumor necrosis factor receptor‐1	sTNF‐αR1	Plasma	Barbosa et al. [65]	Non‐significant	—	—
			Mansur et al. [33]	Unclear	—	—
			Zazula et al. [69]	Non‐significant	—	—
		Serum	Huang et al. [67]	Non‐significant	—	—
			Huang et al. [68]	**Inverse**	Word list II recall	BD‐I subgroup: *B* = −2.178; SE = 1.010; *p* = 0.033
			Millett et al. [70]	Non‐significant	—	—
Soluble tumor necrosis factor receptor‐2	sTNF‐αR2	Plasma	Barbosa et al. [65]	Non‐significant	—	—
			Mansur et al. [33]	Unclear	—	—
			Zazula et al. [69]	**Inverse**	Groton maze learning test (GML)	*r* = 0.54, *p* = 0.002; multivariable: *p* = 0.035
				**inverse**	Two back task (TWOB)	*r* = −0.49, *p* = 0.005; multivariable: *p* = 0.034
		Serum	Millett et al. [70]	**Inverse**	Stroop test	*r* = −0.2, *p* = 0.005
Tumor necrosis factor‐beta/Lymphotoxin‐alpha	TNF‐*β*/LT‐α	Plasma	Strawbridge et al. [52]	Non‐significant	—	—
Transforming growth factor‐*β*1	TGF‐*β*1	Plasma	Liou et al. [41]	Non‐significant	—	—
C‐reactive protein^b^	CRP	Serum	Huang et al. [67]	Non‐significant	—	—
			Huang et al. [68]	**Inverse**	Word list II recall	BD‐I subgroup: *B* = −2.286, SE = 1.041, *p* = 0.030
			Millett et al. [71]	**Inverse**	MCCB speed of processing	*F*(1) = 6.4, *p* = 0.012
				**Inverse**	MCCB reasoning and problem solving	*F*(1) = 6.8, *p* = 0.01
				**Inverse**	Stroop test (executive functioning)	*F*(1) = 4.46, *p* = 0.036
				**Inverse**	Reading mind in the eyes (theory of mind)	*F*(1) = 19.9, *p* < 0.001
				**Inverse**	WCST (executive functioning)	*F*(1) = 6.98, *p* = 0.009
				**Inverse**	MATRICS composite score	BD‐I subgroup: *R* = −0.199, *p* = 0.008
			Sanchez‐Autet et al. [72]	**Inverse**	SCIP total raw score	Female subgroup: *r* = −0.270, *p* = 0.002; Adjusted: B = −0.222, *p* = 0.019
				**Inverse**	Delayed verbal learning	Female subgroup: *r* = −0.198, *p* = 0.026
				**Inverse**	Immediate verbal learning	Female subgroup: *r* = −0.208, *p* = 0.020
				**Inverse**	Verbal fluency	Female subgroup: *r* = −0.329, *p* < 0.001
			Tanaka et al. [59]	Non‐significant	—	—
			Peters et al. [73]	**Inverse**	Affective Go/No GO: negative D prime	Multivariable model for logCRP: B = −0.382, SE = 0.161, *p* = 0.019
				**Inverse**	Negative commissions response times	Multivariable model for logCRP: B = −54.21, SE = 19.293, *p* = 0.006
				**Inverse**	Positive commissions response times	Multivariable model for logCRP: B = −51.155, SE = 20.572, *p* = 0.015
				**Inverse**	Negative hits response times	Multivariable model for logCRP: B = −46.677, SE = 14.138, *p* = 0.001; Stepwise regression = B = −0.002, SE = 0.001, *p* = 0.014
		Plasma	Liou et al. [41]	Unclear	—	—
			Strawbridge [52]	Non‐significant	—	—
		‘Blood samples’	King et al. [44]	Unclear	—	—
High sensitivity C‐reactive protein^b^	hsCRP	Serum	Dickerson et al. [74]	**Inverse**	> 90% total RBANS (dichotomous)	OR 4.32, CI (1.29–14.52), *p* = 0.018
				**Inverse**	> 75% total RBANS (dichotomous)	OR 3.07, CI (1.12–8.44), *p* = 0.030
				**Inverse**	> 90% delayed memory (dichotomous)	OR 3.96, CI (1.16–13.4), *p* = 0.028
				**Inverse**	total RBANS	t = −2.48, *p* = 0.015
				**Inverse**	RBANS immediate memory	t = −2.16, *p* = 0.033
				**Inverse**	RBANS attention	t = −2.18,*p* = 0.032
				**Inverse**	RBANS language	t = −2.13, *p* = 0.036
				**Inverse**	TMT‐A	t = −2.39, *p* = 0.019
Ultrasensitive C‐reactive protein^b^	PCR‐us	Serum	Garés‐Caballer et al. [37]	**Inverse**	Processing speed (at 1 year FU)	Predictive model with PCR‐us, GSH, SOD explained 29.9% of variance (*R* ^2^) of processing speed. PCR‐us: *β* = −0.34, 95% CI (−0.26 to 0.00), *p* < 0.05
				**Inverse**	Verbal fluency (at 1 year FU)	Predictive model with IL‐6, PCR‐us, SOD, HGB, HCT explained 49.8% of variance (*R* ^2^) of verbal fluency. PCR‐us: *β* = −0.53, 95% CI (−0.30 to −0.03), *p* < 0.05
soluble cluster of differentiation 14	sCD14	Serum	Tanaka et al. [59]	Non‐significant	—	—
		CSF	Rolstad et al. [64]	Non‐significant	—	—
Tissue inhibitor of metalloproteinase‐1	TIMP1	CSF	Rolstad et al. [64]	Non‐significant	—	—
Tissue inhibitor of metalloproteinase‐2	TIMP2	CSF	Rolstad et al. [64]	Unclear	—	—
Matrix metallopeptidase 9	MMP9	Serum	Reininghaus et al. [75]	**Inverse**	d2 test of attention	*r* = −0.287, *p* = 0.018
		Plasma	Knöchel et al. [49]	Non‐significant	—	—
Intercellular adhesion molecule 1	ICAM‐1	Plasma	Strawbridge et al. [52]	Non‐significant	—	—
Soluble intercellular adhesion molecule 1	sICAM‐1	Serum	Reininghaus et al. [75]	Non‐significant	—	—
Vascular cell adhesion molecule‐1	VCAM‐1	Plasma	Strawbridge et al. [52]	Non‐significant	—	—
Monocyte chemoattractant protein‐1	MCP‐1/CCL2	CSF	Rolstad et al. [76]	Non‐significant	—	—
		Plasma	Poletti et al. [53]	**Inverse**	Verbal memory	Wald = 5.40, B = 0.04, SE = 0.02, *p* = 0.020
			Strawbridge et al. [52]	Non‐significant	—	—
Macrophage inflammatory protein‐1α	MIP‐1α/CCL3	Plasma	Poletti et al. [53]	Non‐significant	—	—
			Strawbridge et al. [52]	Non‐significant	—	—
Macrophage inflammatory protein‐1*β*	MIP‐1*β*/CCL4	Plasma	Poletti et al. [53]	**Inverse**	Working memory	Wald = 7.75, B = 0.08, SE = 0.02, *p* = 0.005
					Executive functions	Wald = 4.40, B = 0.05, SE = 0.02, *p* = 0.035
			Strawbridge et al. [52]	Non‐significant	—	—
Regulated upon Activation, Normal T Cell Expressed and Presumably Secreted	RANTES/CCL5	Plasma	Poletti et al. [53]	**Inverse**	Working memory	Wald = 7.2, B = 0.0007, SE = 0.0002, *p* = 0.007
Eotaxin‐1	CCL11	Plasma	Poletti et al. [53]	Non‐significant	—	—
			Strawbridge et al. [52]	Non‐significant	—	—
Eotaxin‐3	CCL26	Plasma	Strawbridge et al. [52]	Non‐significant	—	—
Thymus and activation‐regulated chemokine	TARC/CCL17	Plasma	Strawbridge et al. [52]	Non‐significant	—	—
Monocyte chemoattractant protein‐4	MCP‐4/CCL13	Plasma	Strawbridge et al. [52]	Non‐significant	—	—
Chitinase‐3‐like‐1 protein	YKL‐40	CSF	Rolstad et al. [76]	**Inverse**	Executive functions	B = −0.99, *p* < 0.0005; Bootstrapped: CI (0.000015 to 0.000003), *p* = 0.006
Serum amyloid‐A	SAA	Plasma	Strawbridge et al. [52]	Non‐significant	—	—
Alpha 1 antitrypsin	A1AT	Plasma	Knöchel et al. [49]	Non‐significant	—	—
Complement protein (components): 3, C4A, C9, C1q, Factor B, Ficolin‐3, C1‐inhibitor/C1 esterase inhibitor	CO3, CO4A, CO9, C1QC, CFAB, FCN3, IC1	Plasma	Knöchel et al. [49]	Non‐significant	—	—
Ceruloplasmin	CERU	Plasma	Knöchel et al. [49]	Non‐significant	—	—
Haptoglobin	HPT	Plasma	Knöchel et al. [49]	Non‐significant	—	—
Haptoglobin related protein	HPTR	Plasma	Knöchel et al. [49]	Non‐significant	—	—
Histidine‐rich glycoprotein	HRG	Plasma	Knöchel et al. [49]	Non‐significant	—	—
Pigment epithelium‐derived factor	PEDF	Plasma	Knöchel et al. [49]	Non‐significant	—	—
Transferrin	TRFE	Plasma	Knöchel et al. [49]	Non‐significant	—	—

g. Serostatus to infectious agents (*n* = 11)						
Biomarker	Biomarker abbreviation	Type of biofluid	Study	Relationship with cognitive functioning	Cognitive outcome	Effect size and standard error/95% CI^a^
			First author (year)	**Positive =** higher biomarker level related to better cognition; **Inverse =** higher biomarker level related to worse cognition; **Non‐significant =** relationship not statistically significant; **Unclear =** unclear direction of effect or data not shown	Reported only for significant relationships	Reported only for significant relationships
Herpes simplex virus type 1 IgG^b^	HSV‐1 IgG	Serum	Dickerson et al. [77]	**Inverse**	RBANS total score	Multivariate: *F* = 13.7, *p* < 0.001
				**Inverse**	RBANS Immediate Memory	*F* = 12.1, *p* = <.0007
				**Inverse**	RBANS Visuospatial/constructional	*F* = 8.3, *p* < 0.005
				**Inverse**	RBANS Attention	*F* = 10.0, *p* < 0.002
			Dickerson et al. [78]	**Inverse**	RBANS total score	*F*(2, 104) = 16.3, *p* < 0.0001
				**Inverse**	≤ 20% total RBANS (dichotomous)	22.2% relative risk of being in the lowest quintile of RBANS total score: 95% CI (3.2 to 155.0), *p* < 0.002
			Gerber et al. [79]	**Inverse**	Letter Number Sequencing Test (LNST)	Univariate: *F*(2, 104) = 10.4, *p* < 0.002; Multivariate: *F*(2,104) = 5.4, *p* < 0.022
				**Inverse**	Verbal subscore	Overall Wilks λ = 0.778, *F* = 7.995, *p* = 0.009
				**Inverse**	Attention subscore	Overall Wilks λ = 0.678, *F* = 4.514, *p* = 0.046
			Tanaka et al. [59]	Non‐significant	—	—
Herpes simplex virus type 2 IgG	HSV‐2 IgG	Serum	Dickerson et al. [77]	Non‐significant	—	—
			Gerber et al. [79]	Unclear	—	—
			Tanaka et al. [59]	Non‐significant	—	—
Cytomegalovirus IgG	CMV IgG	Serum	Dickerson et al. [77]	Non‐significant	—	—
			Gerber et al. [79]	Non‐significant	—	—
			Tanaka et al. [59]	**Inverse**	BACS total score	*β* = −1.069, *p* = 0.019; Adjusted: *β* = −0.950, *p* = 0.044
Cytomegalovirus IgM	CMV IgM	Serum	Tanaka et al. [59]	Non‐significant	—	—
Epstein–Barr virus IgG	EBV IgG	Serum	Dickerson et al. [77]	Non‐significant	—	—
Human Herpes virus 6/Roseola/exanthem subitum/sixth disease IgG	HHV‐6 IgG	Serum	Dickerson et al. [77]	Non‐significant	—	—
			Gerber et al. [79]	Unclear	—	—
Human Herpes virus 3/varicella zoster virus/chickenpox/shingles	HHV‐3/VZV IgG	Serum	Dickerson et al. [77]	Non‐significant	—	—
Toxoplasma gondii IgG	T gondii IgG	Serum	Dickerson et al. [77]	Non‐significant	—	—
			Gerber et al. [79]	Unclear	—	—
			Tanaka et al. [59]	Non‐significant	—	—
Toxoplasma gondii IgM	T gondii IgM	Serum	Dickerson et al. [80]	**Inverse**	RBANS total	B = −3.84; 95% CI (−7.41 to −0.28), *p* = 0.035
				**Inverse**	RBANS delayed memory	B = −4.79; 95% CI (−9.35 to −0.23), *p* = 0.040
				**Inverse**	RBANS visuospatial/constructional	B = −6.59; 95% CI (−10.49 to −2.70), *p* = 0.001
			Tanaka et al. [59]	Non‐significant	—	—
Influenza B IgG		Serum	Gerber et al. [79]	unclear	—	—
Immunoglobulin heavy constant *μ*	IGHM	Plasma	Knöchel et al. [49]	Non‐significant	—	—

h. Amino acids, vitamins, and minerals (*n* = 7)						
Biomarker	Biomarker abbreviation	Type of biofluid	Study	Relationship with cognitive functioning	Cognitive outcome	Effect size and standard error/95% CI^a^
			First author (year)	**Positive =** higher biomarker level related to better cognition; **Inverse =** higher biomarker level related to worse cognition; **Non‐significant =** relationship not statistically significant; **Unclear =** unclear direction of effect or data not shown	Reported only for significant relationships	Reported only for significant relationships
Homocysteine^b^	Hcy	Plasma	Dittmann et al. [81]	**Inverse**	RBANS Immediate Memory	B = −3.282, SE = 1.248, *F* = 2.87, *p* = 0.024
				**Inverse**	TMT‐B	B = 5.679, SE = 2.607, *F* = 3.97, *p* = 0.004
			Dittmann et al. [82]	**Inverse**	Verbal learning	*β* = −0.35, *p* = 0.002, estimated difference = −0.171 to −0.040
				**Inverse**	Delayed memory	*β* = −0.24, *p* = 0.030, estimated difference = −0.118 to −0.006
				**Inverse**	Executive function	*β* = −0.28, *p* = 0.011, estimated difference = −0.141 to −0.019
		Serum	Aydemir et al. [47]	Non‐significant	—	—
			Chen et al. [42]	**Inverse**	delayed recall	B = −0.15, SE = 0.06; *β* = −0.28, *p* = 0.018
				**Inverse**	CTT‐1	B = 2.54, SE = 0.99; *β* = 0.28, *p* = 0.013
				**Inverse**	Verbal fluency	B = −0.20, SE = 0.10; *β* = −0.25, *p* = 0.041
				**Inverse**	DSST	B = −0.78, SE = 0.34; *β* = −0.23, *p* = 0.026
			Doganavsargil‐Baysal et al. [83]	**Positive** (non‐significant after Bonferroni)	RAVLT max learning	*r* = −0.334, *p* = 0.009 (non‐significant after Bonferroni correction)
			Na et al. [84]	**Inverse**	TMT‐A	*r* = 0.332, *p* = 0.036
				**Inverse**	TMT‐interference	*r* = 0.389, *p* = 0.030
				**Inverse**	TMT‐B	*r* = 0.408, *p* = 0.007
				**Inverse**	Stroop C test correct number	*r* = −0.603, *p* = < 0.001
				**Inverse**	SCWT correct number	*r* = −0.577, *p* = < 0.001
			Osher et al. [85]	**Inverse**	WCST categories completed	High Hcy group significantly worse cognition: *F* = 9.11, *p* = 0.004
				**Inverse**	WCST perseverative errors	High Hcy group significantly worse cognition: *F* = 6.52, *p* = 0.020
			Permoda‐Osip et al. [86]	**Inverse**	Stroop test part B‐ number of errors	Male subgroup: *r* = 0.43, *p* = 0.020
				**Inverse**	WCST total errors	Male subgroup: *r* = 0.56, *p* = 0.003
				**Inverse**	WCST non‐perseverative errors	Male subgroup: *r* = 0.54, *p* = 0.004
				**Inverse**	Stroop test part B ‐ time	Female BD‐II subgroup: *r* = 0.28, *p* = 0.014
			Sanchez‐Autet et al. [72]	Non‐significant	—	—
Methionine		Serum	Doganavsargil‐Baysal et al. [83]	**Positive** (non‐significant after Bonferroni)	RAVLT long‐term memory error score	*r* = −0.288, *p* = 0.026 (non‐significant after Bonferroni correction)
Folic acid/folate/vitamin B9	vit B9	Serum	Aydemir et al. [47]	Non‐significant	—	—
			Bell et al. [40]	Non‐significant	—	—
			Chen et al. [42]	Non‐significant	—	—
			Doganavsargil‐Baysal et al. [83]	Unclear	—	—
			Na et al. [84]	Non‐significant	—	—
			Osher et al. [85]	Non‐significant	—	—
			Permoda‐Osip et al. [86]	**Inverse**	Stroop test part B ‐ number of errors	Total sample: *r* = 0.20, *p* = 0.016; male subgroup: *r* = 0.44, *p* = 0.018
Cobalamin/vitamin B12	vit B12	Serum	Aydemir et al. [47]	Non‐significant	—	—
			Bell et al. [40]	Non‐significant	—	—
			Chen et al. [42]	Unclear	—	—
			Doganavsargil‐Baysal et al. [83]	**Positive** (non‐significant after Bonferroni)	Cancellation test	*r* = −0.294, *p* = 0.023 (non‐significant after Bonferroni correction)
				**Positive** (non‐significant after Bonferroni)	TMT‐A	*r* = −0.322, *p* = 0.012 (non‐significant after Bonferroni correction)
				**Positive** (non‐significant after Bonferroni)	Stroop test	*r* = −0.261, *p* = 0.044 (non‐significant after Bonferroni correction)
				**Positive** (non‐significant after Bonferroni)	RAVLT reaching criteria	*r* = −0.301, *p* = 0.021 (non‐significant after Bonferroni correction)
				**Positive** (non‐significant after Bonferroni)	RAVLT immediate recall	*r* = 0.271, *p* = 0.037 (non‐significant after Bonferroni correction)
			Osher et al. [85]	Non‐significant	—	—
			Permoda‐Osip et al. [86]	**Inverse**	Stroop test part A ‐ number of errors	Female BD‐II subgroup: *r* = 0.38, *p* = 0.001
25‐Hydroxyvitamin D/vitamin D	25(OH)D/vit D	Plasma	Van Rheenen et al. [87]	Non‐significant	—	—
		Serum	Chen et al. [63]	Younger age: **inverse**, older age: **positive**	Composite score	Younger age subgroup: *β* = −0.344, 95% CI (−0.671 to −0.016), *p* = 0.040. older age subgroup: *β* = 0.347, 95% CI (0.054–0.640), *p* = 0.020
				Younger age: **inverse**, older age: **positive**	Verbal fluency	Younger age subgroup: *β* = −0.230, 95% CI (−0.347 to −0.113), *p* < 0.001. older age subgroup: *β* = 0.252, 95% CI (0.114–0.390), *p* < 0.001
				**Inverse**	Processing speed	Younger age subgroup: *β* = −0.409, 95% CI(−0.677 to −0.141), *p* = 0.003
Retinol‐binding protein 4	RET4	Plasma	Knöchel et al. [49]	Non‐significant	—	—
zinc		Serum	Jonsson et al. [88]	Non‐significant	—	—

i. Metabolic factors (*n* = 23)						
Biomarker	Biomarker abbreviation	Type of biofluid	Study	Relationship with cognitive functioning	Cognitive outcome	Effect size and standard error/95% CI^a^
			First author (year)	**Positive =** higher biomarker level related to better cognition; **Inverse =** higher biomarker level related to worse cognition; **Non‐significant =** relationship not statistically significant; **Unclear =** unclear direction of effect or data not shown	Reported only for significant relationships	Reported only for significant relationships
High‐density lipoprotein cholesterol	HDL	Serum	Hui et al. [89]	**Positive**	RBANS total score	*β* = 8.63, t = 3.00, *p* = 0.006
				**Positive**	Immediate memory	*β* = 6.92, t = 2.14, *p* = 0.04
				**Positive**	Language	*β* = 8.35, t = 2.09, *p* = 0.04
		Plasma	Salvi et al. [90]	**Positive** (non‐significant after adjustment)	Composite verbal memory	*β* = 0.20, t(91) = 2.13, *p* = 0.04; adjusted: *F*(1,82) = 3.52, *p* = 0.06.
Total cholesterol		Plasma	Salvi et al. [90]	Non‐significant	—	—
Fasting plasma glucose	FPG	Plasma	Li et al. [45]	Unclear	—	—
			Salvi et al. [90]	Unclear	—	—
Fasting basal insulin		Plasma	Li et al. [45]	Unclear	—	—
			Salvi et al. [90]	Unclear	—	—
Leptin		Plasma	Mansur et al. [33]	**Inverse**	DSST (total number of symbols) at baseline	RR = 0.951, *p* = 0.017
				**Inverse**	RAVLT (immediate recall) at baseline	RR = 0.932, *p* = 0.024
				**Inverse**	RAVLT (delayed recall) at baseline	RR = 0.983, *p* = 0.01
Oxidative damage, measured by the levels of lipid peroxidation using the thiobarbituric acid reactive substances (TBARS) method	TBARS	Serum	Mora et al. [50]	Non‐significant	—	—
Triglycerides	TGC	Plasma	Van Rheenen et al. [91]	**Inverse**	TMT‐B (cognitive flexibility)	*r* = 0.613, *p* = 0.002; adjusted: *F*(1,21) = 12.65, *p* = 0.002
			Salvi et al. [90]	**Inverse** (non‐significant after adjustment)	Composite verbal memory	*β* = −0.21, t(92) = −2.02, *p* < 0.05; adjusted: *F*(1,82) = 0.07, *p* = 0.79
Apolipoproteins: A1, A2, A4, B, C1, C2, C3, C4, D, E, F, H, L1, M, O, J (Clusterin)	APOA1, APOA2, APOA4, APOB, APOC1, APOC2, APOC3, APOC4, APOD, APOE, APOF, APOH, APOL1, APOM, APOO, CLUS	Plasma	Knöchel et al. [49]	APOC3: **inverse** all others: Non‐significant	TMT‐B	APOC3: *r* = −0.49, *p* = 0.008
phosphatidylinositol‐glycan‐specific phospholipase D	PHLD	Plasma	Knöchel et al. [49]	Non‐significant	—	—

j. Hemogram, coagulation, and fibrinolysis markers (*n* = 18)						
Biomarker	Biomarker abbreviation	Type of biofluid	Study	Relationship with cognitive functioning	Cognitive outcome	Effect size and standard error/95% CI^a^
			First author (year)	**Positive =** higher biomarker level related to better cognition; **Inverse =** higher biomarker level related to worse cognition; **Non‐significant =** relationship not statistically significant; **Unclear =** unclear direction of effect or data not shown	Reported only for significant relationships	Reported only for significant relationships
Total white blood cells	Total WBC	Serum	Garés‐Caballer et al. [37]	**Inverse**	Executive domain (at 1 year FU)	Predictive model with multiple WBC biomarkers, explained 48% of variance (*R* ^2^) of executive domain. Total WBC: *β* = −9.41, 95% CI (−7.61 to −2.06), *p* < 0.001.
White blood cells—Neutrophils	WLC‐N	Serum	Garés‐Caballer et al. [37]	**Positive**	Working memory (at 1 year FU)	Predictive model with multiple WBC biomarkers, explained 54.4% of variance (*R* ^2^) of working memory. WBC‐N: *β* = 3.28, 95% CI (0.39 to 1.15), *p* < 0.0001.
White blood cells—Absolute neutrophils	WBC‐AN	Serum	Garés‐Caballer et al. [37]	**Positive**	Executive domain (at 1 year FU)	Predictive model with multiple WBC biomarkers, explained 48% of variance (*R* ^2^) of executive domain. WBC‐AN: *β* = 10.63, 95% CI (2.90 to 9.55), *p* < 0.001.
White blood cells—Lymphocytes	WBC‐L	Serum	Garés‐Caballer et al. [37]	**Positive**	Working memory (at 1 year FU)	Predictive model with multiple WBC biomarkers, explained 54.4% of variance (*R* ^2^) of working memory. WBC‐L: *β* = 2.28, 95% CI (0.10 to 1.03), *p* < 0.05.
White blood cells—Absolute lymphocytes	WBC‐AL	Serum	Garés‐Caballer et al. [37]	**Positive**	Executive domain (at 1 year FU)	Predictive model with multiple WBC biomarkers, explained 48% of variance (*R* ^2^) of executive domain. WBC‐AL: *β* = 3.09, 95% CI (3.51 to 10.12), *p* < 0.0001.
				**Positive**	Working memory (at 1 year FU)	Predictive model with multiple WBC biomarkers, explained 54.4% of variance (*R* ^2^) of working memory. WBC‐AL: *β* = 0.83, 95% CI (−0.10 to 6.49), *p* < 0.05.
White blood cells—Monocytes	WBC‐M	serum	Garés‐Caballer et al. [37]	**Positive**	Executive domain (at 1 year FU)	Predictive model with multiple WBC biomarkers, explained 48% of variance (*R* ^2^) of executive domain. WBC‐M: *β* = 2.15, 95% CI (0.36 to 2.34), *p* < 0.01.
				**Positive**	Working memory (at 1 year FU)	Predictive model with multiple WBC biomarkers, explained 54.4% of variance (*R* ^2^) of working memory. WBC‐M: *β* = 1.38, 95% CI (0.43 to 2.59), *p* < 0.01.
White blood cells—Absolute monocytes	WBC‐AM	serum	Garés‐Caballer et al. [37]	**Inverse**	Executive domain (at 1 year FU)	Predictive model with multiple WBC biomarkers, explained 48% of variance (*R* ^2^) of executive domain. WBC‐AM: *β* = −2.24, 95% CI (23.08 to −2.07), *p* < 0.05.
				**Inverse**	Working memory (at 1 year FU)	Predictive model with multiple WBC biomarkers, explained 54.4% of variance (*R* ^2^) of working memory. WBC‐AM: *β* = −1.31, 95% CI (−24.9 to −0.62), *p* < 0.05.
Red blood cells	RBC	serum	Garés‐Caballer et al. [37]	Non‐significant	—	—
Hemoglobin	HGB	Serum	Garés‐Caballer et al. [37]	**Positive**	Cognitive flexibility (at 1 year FU)	Predictive model with HCT and HGB explained 26.4% of variance (*R* ^2^) of cognitive flexibility. HGB: *β* = 1.53, 95% CI (0.54 to 3.14), *p* < 0.01.
				**Positive**	Verbal fluency (at 1 year FU)	Predictive model with IL‐6, PCR‐us, SOD, HGB, HCT explained 49.8% of variance (*R* ^2^) of verbal fluency. HGB: *β* = 1.17, 95% CI (0.14 to 1.46), *p* < 0.05.
Hematocrit	HCT	Serum	Garés‐Caballer et al. [37]	**Inverse**	Cognitive flexibility (at 1 year FU)	Predictive model with HCT and HGB explained 26.4% of variance (*R* ^2^) of cognitive flexibility. HCT: *β* = −1.29, 95% CI (−0.98 to −0.08), *p* < 0.05.
				**Inverse**	Verbal fluency (at 1 year FU)	Predictive model with IL‐6, PCR‐us, SOD, HGB, HCT explained 49.8% of variance (*R* ^2^) of verbal fluency. HCT: *β* = −0.91, 95% CI (−0.44 to 0.01), *p* < 0.05.
Blood platelets	PLT	Serum	Garés‐Caballer et al. [37]	Non‐significant	—	—
Neutrophil–lymphocyte ratio	NLR	Serum	Sağlam Aykut et al. [92]	**Inverse**	Stroop interference	*r* = −0.383, *p* < 0.05
Platelet–lymphocyte ratio	PLR	Serum	Sağlam Aykut et al. [92]	Non‐significant	—	—
Alpha 2 antiplasmin/plasmin inhibitor	A2AP	Plasma	Knöchel et al. [49]	Non‐significant	—	—
Alpha 2 macroglobulin	A2MG	Plasma	Knöchel et al. [49]	Non‐significant	—	—
Coagulation factor VIII B/clotting factor	F13B	Plasma	Knöchel et al. [49]	Non‐significant	—	—
Plasma kallikrein	KLKB1	Plasma	Knöchel et al. [49]	Non‐significant	—	—
Plasminogen	PLMN	Plasma	Knöchel et al. [49]	Non‐significant	—	—

^a^ In case of multiple models (e.g., unadjusted, adjusted for some variables, adjusted for many variables), only the results of the most adjusted model were reported.

^b^ This biomarker is one of the three biofluid biomarkers most consistently linked to cognition in BD, see also Table 2 for details.

The majority of 60 studies investigated multiple biomarkers within one study. Most of the 184 biomarkers were measured in plasma (*n* = 96) or serum (*n* = 64), but CSF (*n* = 21), spot urine (*n* = 2), whole blood (*n* = 1), and saliva (*n* = 1) were also investigated.

Cognitive measurements in the studies were heterogeneous and ranged from one test (domain‐specific) to extensive multi‐domain neuropsychological testing (Appendix S5, Table S1). Across studies, cognitive outcomes therefore ranged from unadjusted, raw, subitem test scores to composite scores for global cognitive functioning based on many standardized tests. Full test batteries that were often used were the Repeatable Battery for the Assessment of Neuropsychological Status (RBANS), Cambridge Neuropsychological Test Automated Battery (CANTAB), the MATRICS Consensus Cognitive Battery (MCCB), the Brief Assessment of Cognition in Schizophrenia (BACS), and the Brief Assessment of Cognition in Affective Disorders (BAC‐A). Almost all studies used continuous outcomes for cognitive functioning. Strawbridge et al. used a dichotomous outcome for “cognitive impairment” alongside a continuous outcome for global cognitive performance [52]. Poletti et al. created dichotomous outcomes (poor versus good cognitive performance) for seven cognitive domains based on BACS scores [53].

### 3.3 Methodological Quality Assessment

The scoring for the quality assessment and sample sizes of each study can be found in Appendix S5, Table S2. Some articles had overlapping study samples, but each article was evaluated separately because often different statistical analyses were used and quality of reporting varied per article. Out of 60 articles, the methodological quality was considered good in 4, fair in 27, and poor in 29 articles. Out of the four articles with good quality, three articles had overlapping samples and mostly overlapping authors (Dickerson et al. [77, 78, 80]).

Many of the included studies used small sample sizes and performed bivariate correlation analyses without any adjustment for confounders or correction for multiple testing, increasing the risk of false positive results (type I error) and false negative findings (type II error) [93]. In 46 out of 60 studies, some form of correction was performed, mostly controlling for age, sex, education level, but sometimes also for bipolar subtype, mood symptoms, and physical health indicators (e.g., smoking, alcohol use, BMI). None of the included studies controlled for recurrence of episodes, suicidality, and/or comorbid physical diseases (Appendix S5, Table S2).

Twenty studies (33%) had sample sizes smaller than 50 BD patients and 17 studies (28%) larger than 100 BD patients. Often, studies measured a large set of biomarkers in relation to a large set of cognitive outcomes. As an example, Jakobsson et al. performed correlation analyses between ten biomarkers and nine cognitive outcomes, which resulted in 90 correlation analyses [39]. Knöchel et al. used three cognitive tests as outcomes, but investigated 42 plasma proteins and thus performed 126 bivariate correlation analyses [49]. Another point of concern was that studies often used single neuropsychological test scores as cognitive outcomes. These specific, raw, single (subitem) scores were not standardized based on age, education level and/or sex by comparison to a healthy control group or normative data. Overall, the risks of multiple testing, publication bias, and reporting bias were high, making the available evidence preliminary and of limited quality.

### 3.4 Biofluid Biomarkers in Relation to Cognition in BD

This systematic review identified 184 different (combinations of) biofluid biomarkers that were measured in relation to cognition in BD. We organized these into ten biomarker categories. An overview of all studied biomarkers and the reported relationship with cognitive functioning in BD is shown in Table 1a–j. In this review, a *positive* relationship means that a higher biomarker level was significantly associated with better cognitive functioning, whereas an *inverse* relationship means that a higher biomarker level was significantly associated with worse cognitive functioning. Most of the significant relationships between biomarkers and cognitive outcomes were inverse, i.e., a higher biomarker level was associated with worse cognitive functioning. See Table 1a–j for the full names of the abbreviated biomarkers described in the text below. See Table 2 for a summary table of findings for the biomarkers with most available evidence (i.e., a biomarker analyzed by four or more studies).

**TABLE 2:** Overview of biofluid biomarkers investigated by four or more studies.

Biomarker	Biomarker abbreviation	Biomarker group	Function	Number of unique BD patients involved^b^	Total number of studies	Number of studies reporting a significant relationship with cognition in BD	Relationship with cognitive functioning	Affected cognitive domains	Study and study quality ^c^
				Total no. of BD patients (range of number of patients per study sample)		No. (% of the total number of studies)	**Positive =** higher biomarker level related to better cognition; **Inverse =** higher biomarker level related to worse cognition; **Non‐significant =** relationship not statistically significant; **Unclear =** unclear direction of effect or data not shown	Reported only for significant relationships	Reported only for significant relationships
**(ultrasensitive/high sensitivity) C‐reactive protein** ^**a**^	**CRP/hsCRP/PCR‐us**	Inflammatory/immune markers	Type I acute phase protein produced by the liver substance the liver produces in response to inflammation. Regulated by pro‐inflammatory cytokines. A high level of CRP is a sensitive marker of (early) infection.	**1552** (13–791)	11 (9 unique samples)	6 (55%) (5 unique samples)	**Inverse:** 6 studies; Non‐significant: 3 studies; Unclear: 2 studies	Cognitive composite score (MATRICS, SCIP, RBANS), attention, speed of processing, reasoning and problem solving, executive functioning, theory of mind, verbal fluency, verbal learning and memory, cognitive‐affective processing	Poor: Garés‐Caballer et al. [37]; Fair: Huang et al. [68], Millett et al. [71], Peters et al. [73], Dickerson et al. [74]; Good: Sanchez‐Autet et al. [72]
Tumor necrosis factor alfa	TNF‐α	Inflammatory/immune markers	An inflammatory cytokine produced by macrophages/monocytes during acute inflammation and is responsible for a diverse range of signaling events within cells, leading to necrosis or apoptosis.	**1380** (13–791)	10	2 (20%)	**Inverse:** 2 studies; Non‐significant: 5 studies; Unclear: 3 studies	Executive function, visual immediate memory	Fair: Liou et al. [41], Millett et al. [70]
**Homocysteine** ^**a**^	**Hcy**	Amino acids, vitamins, and minerals	An amino acid with many bodily functions. Homocysteine is related to vitamin B12 deficiency. In high amounts, homocysteine is a marker for cardiovascular disease. Elevated levels have also been linked to dementia, stroke, and osteoporosis.	**777** (51–224)	9 (8 unique samples)	6 (67%) (5 unique samples)	**Inverse:** 6 studies; Non‐significant: 3 studies	cognitive composite score (RBANS), verbal learning and memory, executive function, attention, speed of processing, verbal fluency, psychomotor speed	Poor: Chen et al. [42]; Na et al. [84]; Osher et al. [85]; Permoda‐Osip et al. [86]. Fair: Dittmann et al. [81], Dittmann et al. [82]
Folic acid/folate/vitamin B9	vit B9	Amino acids, vitamins, and minerals	A water‐soluble B vitamin. It plays an essential role in the conversion of homocysteine into methionine. Folate is necessary for the synthesis of nucleic acids (DNA and RNA), the metabolism of amino acids, the production of healthy red blood cells, and proper cell division in periods of rapid growth, such as pregnancy and fetal development.	**487** (8–116)	7	1 (14%)	**Inverse:** 1 study; Non‐significant: 5 studies; Unclear: 1 study	Executive function	Poor: Permoda‐Osip et al. [86]
Interleukin‐6	IL‐6	Inflammatory**/**immune markers	IL‐6 is a pro‐inflammatory cytokine secreted in response to tissue injury and is primarily produced by macrophages. IL‐6 are sensitive markers of (early) infection. IL‐6 regulates CRP.	**292** (13–84)	7	3 (43%)	**Inverse:** 2 studies; **Positive:** 1 study; Non‐significant: 3 studies; Unclear: 1 study	Presence of cognitive impairment (dichotomous), cognitive composite score (BAC‐A), executive function, verbal fluency	*Inverse:* Poor: Poletti et al. [53]; Fair: Barbosa et al. [65] *Positive:* Poor: Garés‐Caballer et al. [37]
Cobalamin**/**vitamin B12	vit B12	Amino acids. Vitamins, and minerals	A water‐soluble B vitamin. Together with folate, vitamin B12 is necessary for formation of red blood cells and DNA, and the metabolism of fatty acids and amino acids. It is vital for a normal function of the nervous system as it plays a role in myelin synthesis.	**387** (8–116)	6	1 (17%)	**Inverse:** 1 study; Non‐significant: 4 studies; Unclear: 1 study	Executive function, verbal learning and memory, attention, psychomotor speed	Poor: Permoda‐Osip et al. [86]
Soluble tumor necrosis factor receptor‐1	sTNF‐αR1	Inflammatory**/**immune markers	A free circulating form of the membrane‐bound TNF receptor (mTNFR1). The soluble receptor competes with the membrane‐bound TNF receptor, leading to decreased availability of circulating TNFα.	**347** (31–219)	6 (4 unique samples)	1 (17%)	**Inverse:** 1 study; Non‐significant: 4 studies; Unclear: 1 study	Verbal learning and memory	Fair: Huang et al. [68]
Interleukin‐10/human cytokine synthesis inhibitory factor	IL‐10**/**CSIF	Inflammatory**/**immune markers	A anti‐inflammatory cytokine that inhibits lipopolysaccharide (LPS) and the secretion of the pro‐inflammatory cytokines, including TNFα and IFNγ.	**266** (20–84)	5	0 (0%)	Non‐significant: 5 studies	—	—
Cortisol		Neuropeptides and hormones	A glucocorticoid hormone produced in the adrenal cortex and part of the hypothalamic–pituitary–adrenal (HPA) axis. Cortisol is a major stress hormone that is secreted during both physical and psychological stress. Prolonged exposure to stress activates the HPA axis, leading to an elevated secretion of cortisol, which affects many bodily tissues, including the brain.	**220** (32–65)	5 (4 unique samples)	2 (40%) (2 unique samples)	**Inverse:** 1 study; **Positive:** 1 study; Non‐significant: 3 studies	Verbal fluency, verbal learning and memory, attention, executive function, token motor	*Inverse:* Poor: Tournikioti et al. [61] *Positive:* Poor: Thompson et al. [62]
**Herpes simplex virus type 1 IgG** ^**a**^	**HSV‐1 IgG**	Serostatus to infectious agents	Antibodies for infection with the herpes simplex virus type 1.	**179** (30–117)	4 (3 unique samples)	3 (75%)	**Inverse:** 3 studies; Non‐significant: 1 study	Cognitive composite score (RBANS), (working) memory, visuospatial/constructional, attention	Poor: Gerber et al. [79]; Good: Dickerson et al. [77]; Dickerson et al. [78]
Soluble tumor necrosis factor receptor‐2	sTNF‐αR2	Inflammatory/immune markers	A free circulating form of the membrane‐bound TNF receptor (mTNFR2). The soluble receptor competes with the membrane‐bound TNF receptor, leading to decreased availability of circulating TNFα.	**330** (20–219)	4	2 (50%)	**Inverse:** 2 studies; Non‐significant: 1 study; Unclear: 1 study	Executive function, working memory	Fair: Zazula et al. [69]; Millett et al. [70]
Brain‐derived neurotrophic factor	BDNF	Growth factors	A protein molecule that plays a crucial role in learning, memory, neuroplasticity (the ability to form new neural connections and pathways), and adult neurogenesis (the ability to grow new neurons in adults whose brains have reached full maturity). BDNF promotes proliferation, regeneration and survival of neurons.	**942** (23–791)	4	2 (50%)	**Positive:** 2 studies; Non‐significant: 1 study; Unclear: 1 study	Verbal memory, executive function, attention	Fair: Mora et al. [50]; Liou et al. [41]

^a^ Based on the total number of studies per biomarker and the percentage of studies reporting significant findings in a consistent direction, this biomarker was selected as one of the three biofluid biomarkers most consistently linked to cognition in BD (HSV‐1 IgG, CRP, and Hcy). IL‐6 was not selected because of inconsistent directions of effect across studies.

^b^ In case of two studies with overlapping samples, only the largest number of patients was used for this calculation.

^c^ See Supplemental Table S2 for details on the risk of bias assessment.

#### 3.4.1 Oxidative Stress Markers

This review identified five studies that together investigated fourteen oxidative stress markers [34, 37, 47, 48, 49]. Most markers were measured by one single study.

GSH, SOD, and NO were analyzed by two studies, but these showed mixed results. Tozoglu et al. identified significant correlations with cognition for TAC, ADMA, NO, and SDMA, but the quality of evidence for these findings was very low as these correlations were unadjusted and the outcomes were non‐standardized raw subitem test scores [48]. For TAC and ADMA, the reported relationship with cognition was *positive*, whereas for NO and SDMA the relationship was *inverse*. Garés‐Caballer et al. performed a prospective, 1‐year follow‐up cohort study and created predictive models with combinations of various biomarkers at baseline as predictors and cognitive domain scores at 1‐year FU as outcomes [37]. In this study, GSH and SOD were positively related to processing speed [37], but the study by Aydemir et al. did not identify significant relationships for GSH, SOD, or NO [47]. Non‐significant relationships were observed for MDA, NT‐4, ROS, mROS, 8‐oxo‐Guo, 8‐oxo‐dG, ANT3, and HEP2.

#### 3.4.2 Growth Factors

Thirteen growth factors were examined by seven studies [34, 41, 50, 51, 52, 53, 54].

BDNF was examined by four studies, of which two reported significant associations with cognition. Mora et al. [50] and Liou et al. [41] both described a *positive* association: higher BDNF levels were related to better cognition, with the domains of executive functioning and verbal memory [50] and with specific items of the Continuous Performance Test [41], respectively. Chou et al. also studied BDNF using six neuropsychological tests with a total of 48 subitem test scores [51]. Of all these, BDNF was significantly correlated with cognition in only two out of 48 analyses, but the direction of effect was not clearly stated. Strawbridge et al. did not observe a significant relationship between BDNF and cognition [52].

The results for other growth factors were mixed. The basic fibroblast growth factor (bFGF) was significantly related to cognition in two studies, but the reported direction of effect was opposite [52, 53]. Strawbridge et al. reported significant *positive* associations for PlGf and VEGF‐C with outcome global cognitive performance as well as outcome cognitive impairment [52]. Omileke et al. reported a significant *positive* correlation with FGF21 and letter fluency and motor speed [54]. No significant relationships were reported for EPO, Flt‐1, Tie‐2, PDGF‐BB, G‐CSF, GM‐CSF, VEGF, and VEGF‐D.

#### 3.4.3 Neurotransmitters

Four studies measured fourteen neurotransmitters or related compounds in relation to cognition in BD [35, 44, 55, 56]. Some significant relationships were identified, but these were not consistent across studies. KYNA was significantly associated with global cognitive functioning as well as BACS‐SC (processing speed) by Hebbrecht et al. [35], but Paribello et al. and Platzer et al. did not find significant associations with KYNA [55, 56]. Higher 5‐HTP was significantly associated with worse performance on the BAC‐A color naming subtask, but only for BD subgroups with lifetime suicide attempts or ideations [55]. Platzer et al. found that the 3‐HK/KYNA ratio was inversely related and KYN/3‐HK ratio positively related to the California Verbal Learning Test (CVLT), but only in BD males [56]. No significant associations were noted for Glu, 5‐HT, TRP, QA, KYN, 3‐HK, 5‐HTP/TRP ratio, KYN/KYNA ratio, KYN/TRP*1000 ratio, or the QA/KYNA ratio.

#### 3.4.4 Neuropeptides and Hormones

Regarding the biomarker category of neuropeptides and hormones, fourteen different compounds were investigated by eleven studies [32, 36, 42, 46, 49, 57, 58, 59, 60, 61, 62].

Cortisol was studied by five studies, of which two reported significant relationships, but with opposite directions of effect. A higher cortisol serum level was significantly associated with *worse* performance on the paired associative learning test (PAL) for visuospatial functioning [61]. However, Thompson et al. found that higher cortisol levels in saliva were associated with *better* cognitive performance on the Stroop, the Tower of London, and COWAT [62]. Lee et al. performed an RCT in which all patients received open label valproate [38]. At baseline, cortisol and cognition were not significantly associated [38]. In addition, Tournikioti et al. reported a significant association between cortisol/DHEA‐S ratio and stockings of Cambridge test for executive function [60]. None of the other investigated neuropeptides or hormones (α‐MSH, β‐endorphin, neurotensin, oxytocin, substance P, pentraxin‐3, arginine vasopressin, CBG, DHEA, pregnenolone, T3) were significantly associated with cognition.

#### 3.4.5 Neurodegenerative Markers

This review identified three studies that together explored eleven (combinations of) neurodegenerative biomarkers in relation to cognition in BD patients [39, 63, 64], but the results were inconsistent.

Regarding neurofilament light chain (NfL), two studies described significant *inverse* relationships with cognition in BD: with composite cognitive score, verbal fluency, and processing speed [63], with memory, and with verbal functions [64]. However, Chen et al. studied NfL in serum [63], whereas Rolstad et al. measured NfL in CSF [64]. Chen et al. also identified an opposite, *positive* relationship between NfL and verbal fluency for older patients [63].

Soluble amyloid precursor protein alfa (sAPP‐α) was significantly associated with cognition in two studies: *positively* with trail making test (TMT) by Jakobsson et al. [39] but *inversely* with attention/speed by Rolstad et al. [64]. Regarding T‐Tau, two studies also described significant relationships, but the direction of effect was opposite. Inconsistent findings were also reported for Amyloid beta 42/40 ratio (Aβ42/40 ratio) and Amyloid beta 42/38 ratio (Aβ42/38 ratio): The study by Rolstad et al. reported significant relationships [64], but the study by Jakobsson et al. did not [39].

#### 3.4.6 Inflammatory/Immune Markers

Of all the biomarkers included in this review, by far most fell into the inflammatory/immune markers category. In total, 59 different inflammatory biomarkers, among which 17 interleukins, were examined across 21 studies [33, 37, 41, 43, 44, 50, 52, 53, 59, 65, 66, 67, 68, 69, 70, 71, 72, 73, 74, 75, 76]. The inflammatory markers that were assessed most often were (high/ultra‐sensitive) C reactive protein (CRP/hsCRP/PCR‐us, eleven studies), tumor necrosis factor alpha (TNF‐α, ten studies), interleukin‐6 (IL‐6, seven studies), soluble tumor necrosis factor receptor‐1 (sTNF‐αR1, six studies), interleukin‐10 (IL‐10, five studies), and soluble tumor necrosis factor receptor‐2 (sTNF‐αR2, four studies).

Out of the 17 interleukins examined, most studies reported on IL‐6, but results were inconsistent. In plasma, Barbosa et al. [65] and Poletti et al. [53] found significant *inverse* relationships (higher IL‐6, worse total BAC‐A z‐score and executive functions, respectively). In serum, however, Garés‐Caballer et al. [37] noted a *positive* relationship between IL‐6 and verbal fluency. Moreover, Civil Arslan et al., Mora et al., King et al., and Strawbridge et al. could not detect significant relationships for IL‐6 [44, 50, 52, 66].

For CRP, a relatively large amount of evidence was available: significant *inverse* relationships with cognition were reported by six out of eleven studies. Notably, all significant findings for CRP were observed in serum, not in plasma. Across studies, higher CRP levels were associated with worse performance on 25 diverse cognitive outcomes (see Table 1): from very specific single subitem scores such as the negative hits response times of the CANTAB affective Go/no‐go task [73] to cognitive composite scores based on several cognitive domains such as the MATRICS composite score [71]. In the study by Sanchez‐Autet et al., significant correlations for CRP were only found for a female subgroup [72]. Huang et al. [68] and Millett et al. [71] both reported that the observed associations of CRP with cognition were significant for BD‐I, but not BD‐II patients. Five studies did not find significant relationships between CRP and cognition in BD.

Regarding TNF‐α, only two out of ten studies reported a significant *inverse* relationship: one in plasma [41] and one in serum [70]. Higher TNF‐α was associated with worse performance on the WCST, Wechsler Memory Scale III (WMS‐III) visual immediate index subtest, and Stroop test [41, 70]. Soluble tumor necrosis factor receptor‐2 (sTNF‐αR2) was found to be significantly inversely related to single cognitive test scores in two out of four studies, both measured in plasma [69] and serum [70].

Considering other inflammatory biomarkers, the following were significantly associated with cognitive outcomes in BD, but each of these biomarkers only by one single study: *inverse* relationships for IL‐1β [53], interleukin‐1 receptor antagonist (IL‐1RA [43]), soluble interleukin‐6 receptor (sIL‐6R [68]), IL‐8 [41], soluble tumor necrosis factor receptor‐1 (sTNF‐αR1 [68]), matrix metallopeptidase 9 (MMP9 [75]), monocyte chemoattractant protein‐1 (MCP‐1/CCL2 [53]), macrophage inflammatory protein‐1β (MIP‐1β/CCL4 [53]), RANTES (CCL5 [53]), and chitinase‐3‐like‐1 protein (YKL‐40 [76]), and *positive* relationships for IL‐7 [52], IL‐16 [52], IL‐17A [53], and IL‐18 [66]. Of note, IL‐10 was not related to cognition in BD in any of five studies.

#### 3.4.7 Serostatus to Infectious Agents

This review identified five studies that investigated eleven antibodies in relation to cognition in BD [59, 77, 78, 79, 80], measuring the serostatus to various infectious agents.

Most evidence was available for herpes simplex virus type 1 (HSV‐1 IgG); three out of four studies reported *inverse* relationships with cognition: with RBANS total score [77, 78], RBANS domain scores [77], the Letter Number Sequencing Test [78], and verbal and attention subscores [79]. Also, higher CMV IgG was associated with worse BACS total score [59] and higher Toxoplasma gondii IgM with worse RBANS total score, RBANS delayed memory, and RBANS visuospatial/constructional [80]. However, other studies could not find significant relationships for the latter two biomarkers [59, 77, 79]. Non‐significant relationships were described for HSV‐2 IgG, CMV IgM, EBV IgG, HHV‐6 IgG, HHV‐3/VZV IgG, T gondii IgG, Influenza B IgG, and IGHM.

#### 3.4.8 Amino Acids, Vitamins, and Minerals

A total of fourteen studies were included that together studied seven different amino acids, vitamins, or minerals [40, 42, 47, 49, 63, 72, 81, 82, 83, 84, 85, 86, 87, 88].

By far most evidence in this category was available for the amino acid homocysteine (Hcy); nine studies were available, of which six reported significant *inverse* relationships with cognition. Across studies, Hcy was significantly *inversely* related to 21 diverse cognitive outcomes (see Table 1): from very specific single subitem scores such as the number of perseverative errors on the WCST [85] to cognitive composite scores for several cognitive domains such as verbal fluency [42]. Permoda‐Osip et al. also identified four *inverse* relationships, but only in male or female subgroups [86]. One study initially described a significant correlation between Hcy and RAVLT, but after adjustment or Bonferroni correction significance disappeared [83]. Two studies reported non‐significant relationships for Hcy [47, 72].

We identified some evidence that both folate/folic acid and vitamin B12 are *not* related to cognitive functioning in BD. Folate was researched by seven studies, but only one study found a significant *inverse* relationship with cognition and this was only with a very specific subitem score (number of errors on the Stroop test part B [86]). Vitamin B12 was researched by six studies, of which only one study reported a significant *inverse* relationship with a very specific subitem score (number of errors on the Stroop test part A [86]). However, the absence of significance may also be due to underpowered studies.

Regarding vitamin D, Chen et al. identified significant relationships with a cognitive composite score, verbal fluency, and processing speed, but the direction of the effect was *inverse* for younger patients and *positive* for older aged BD patients [63]. In contrast, the study by Van Rheenen et al. found no significant association between vitamin D and global cognition in patients with BD [87]. Methionine, RET4, and zinc were not significantly associated with cognition.

#### 3.4.9 Metabolic Factors

This review identified seven studies that investigated a total of 23 metabolic biomarkers [33, 45, 49, 50, 89, 90, 91].

A higher HDL was associated with a better RBANS total score, better immediate memory and language scores in the study by Hui et al. [89]. Also Salvi et al. found a *positive* association between HDL and a composite score of verbal memory, but this association became non‐significant after adjustment [90].

Regarding leptin, an *inverse* association with three cognitive subtests was identified by Mansur et al. [33]. A higher level of triglycerides was related to worse cognition by Van Rheenen et al. [91] and Salvi et al. [90], but the association became non‐significant after adjustment in the latter study.

Knöchel et al. analyzed 42 plasma proteins, in relation to three cognitive tests [49]. Out of all of these analyses, only ApoC3 was *inversely* related to a single cognitive test score (TMT‐B test) [49]. Non‐significant relationships were observed for all other Apo proteins, as well as for clusterin, total cholesterol, FPG, fasting basal insulin, TBARS, and PHLD.

#### 3.4.10 Hemogram, Coagulation and Fibrinolysis Markers

Three studies examined biomarkers from the hemogram, coagulation and fibrinolysis system in relation to cognition in BD [37, 49, 92].

As mentioned earlier, Garés‐Caballer et al. created predictive models with combinations of biomarkers, so the biomarkers were not studied separately in relation to cognition [37]. A predictive model with hemoglobin (HGB), hematocrit (HCT), but also IL‐6, PCR‐us, and SOD explained 49.8% of variance (R2) of verbal fluency. A predictive model with HCT and HGB explained 26.4% of variance of cognitive flexibility. Predictive models with multiple white blood cell (WBC) biomarkers explained 48% of variance of executive domain and 54.4% of variance of working memory [37]. However, the direction of effects differed between biomarkers (*inverse* for total WBC, WBC‐AM, and HCT, but *positive* for WBC‐N, WBC‐AN, WBC‐L, WBC‐AL, WBC‐M, HGB), which makes these results difficult to interpret [37].

Sağlam Aykut et al. found a significant correlation between the neutrophil–lymphocyte ratio (NLR) and Stroop interference, but these analyses were unadjusted [92]. Non‐significant relationships were reported for red blood cells, platelets, PLR, A2AP, A2MG, F13B, KLKB1, and PLMN.

## 4 Discussion

### 4.1 Main Findings

Cognitive impairment can be a major clinical challenge for BD patients and has considerable impact on daily functioning, with accumulated burden as patients age. Identifying biological factors that are associated with cognition and easily measured from biofluids may help to identify patients at risk of cognitive impairment and provide adequate monitoring and personalized treatment across the lifespan. This systematic review identified 60 articles, reporting on 184 different (combinations of) biofluid biomarkers that have been researched in relation to cognitive functioning in BD. We organized these biomarkers into ten categories: oxidative stress markers (*n* = 14); growth factors (*n* = 13); neurotransmitters (*n* = 14); neuropeptides and hormones (*n* = 14); neurodegenerative markers (*n* = 11); inflammatory/immune markers (*n* = 59); serostatus to infectious agents (*n* = 11); amino acids, vitamins, and minerals (*n* = 7); metabolic factors (*n* = 23); hemogram, coagulation, and fibrinolysis markers (*n* = 18). Due to small sample sizes, a high risk of error associated with multiple testing, and thus publication and reporting bias, combined with small effect sizes in most studies, the presented evidence is of relatively low quality and should therefore be interpreted with caution.

Most biomarkers were assessed by a single study, but twelve biomarkers were examined in four or more studies (Table 2). Based on the total number of studies per biomarker and the percentage of studies reporting significant findings in a consistent direction, this review revealed preliminary evidence for *inverse* associations between cognitive functioning in BD and (1) HSV‐1 IgG, (2) CRP, and (3) Hcy; for these biomarkers, higher levels were associated with worse cognition. These three biomarkers were related to a broad range of cognitive outcomes, ranging from very specific single subitem scores to cognitive composite scores based on several cognitive domains (Table 2). The only four articles with quality rating “Good” described significant *inverse* associations for (1) HSV‐1 IgG [77, 78], (2) Toxoplasma Gondii IgM [80], and (3) CRP [72]. Of note, out of these four articles with good quality, three articles had overlapping samples and mostly overlapping authors (Dickerson et al. [77, 78, 80]). Taken together, our findings suggest that previous infections, current inflammation, and/or physical or psychological stress may decrease cognitive functioning in BD.

### 4.2 Interpretation and Mechanisms

#### 4.2.1 CRP

Our finding that CRP is related to cognitive functioning in BD is in line with previous reviews [27, 28, 29]. However, a recent review by Morrens et al., who performed a large systematic review and meta‐analysis on the relationship between immune markers and cognitive impairment in mood and psychotic disorders, reported more pessimistically on the available evidence [94]. Due to wider, transdiagnostic inclusion criteria, Morrens et al. included more studies than are listed in the current review for the respective biomarkers. Although Morrens et al. also reported a (weak) association for CRP and cognitive impairment, they concluded overall that “there is no reliable evidence of a meaningful association between blood‐based immune markers and global or domain specific cognitive performance”; not across diagnoses and neither within the BD diagnostic subgroup. In our review, the good quality study by Sanchez‐Autet et al. showed small to medium effect sizes (*r* = 0.2–0.3), but only in female BD patients [72].

In healthy adults, baseline CRP levels tend to increase slightly with age and higher levels are associated with a higher risk of chronic, aging‐related diseases, including dementia [95]. CRP not only reflects systemic (low‐grade) inflammation, but possibly also accelerates biological aging (*inflammageing*). Although inflammation is normally a protective response that facilitates the healing process, chronic inflammation interferes with the maintenance and repair of all bodily tissues, leading to accumulation of tissue damage [95].

#### 4.2.2 Homocysteine

To our knowledge, this is the first systematic review combining the evidence on homocysteine and cognition in BD. In the articles that reported standardized effect sizes (Table 1), effect sizes ranged from small to medium (*r* = 0.2–0.6) but mainly involved single neuropsychological tests as outcomes. Dittmann et al., who performed a study of “Fair” quality with multiple cognitive domains as outcomes, found small to medium effect sizes (*beta* = −0.24–0.35) for outcomes verbal learning, delayed memory, and executive function [82].

Hcy is a cardiovascular risk factor and promoter of oxidative stress and has been associated with progression from cognitive impairment to dementia [96]. There are several ways in which Hcy could be related to cognitive impairment. Hypertension (as a result of physical or psychological stress) increases Hcy levels [97]. In patients with cognitive impairment induced by hypertension, Hcy levels are also increased [98]. In addition, high Hcy may induce indirect neurotoxicity, which could lead to the death of hippocampal neurons [84]. Last, Hcy is a marker for vitamin B12 deficiency, which in turn can also cause cognitive problems, especially in older people [99].

#### 4.2.3 HSV‐1 IgG

We identified four studies that studied the relationship between HSV‐1 IgG and cognition in BD patients, of which three reported significant *inverse* associations [77, 78, 79]. However, effect sizes were difficult to interpret in all these studies as these were not standardized. The good quality study by Dickerson et al. 2004 found a highly significant difference of 9.1 in total RBANS score between BD (HSV‐1 positive 77.5 and HSV‐1 negative 86.6) [77]. A recent study estimated the minimum clinically important difference (MCID) for the RBANS total score minimally 8, which suggests that Dickerson et al. 2004 found a small, but meaningful clinical difference [100].

According to the WHO, about 67% of the world population under the age of 50 had HSV‐1 in 2012 [101]. Many of those infected never develop symptoms. In the past, chronic or latent HSV infections were considered to be relatively benign and were believed to be only associated with cognitive impairment in case the virus caused acute encephalitis [102]. However, recent literature has identified associations between HSV and cognitive impairment in patients with schizophrenia as well as in healthy individuals without a history of encephalitis. Also, HSV‐1 has been identified as a causative agent in the pathogenesis of sporadic Alzheimer's disease (AD) through neuronal production and accumulation of amyloid beta (Aβ), hyperphosphorylation of tau proteins, dysregulation of calcium homeostasis, and impaired autophagy [103].

### 4.3 General Mechanisms

Taken together, the three biomarkers that were most consistently related to cognition in BD (HSV‐1 IgG, CRP, and Hcy) all play a role in inflammation and/or presence of increased physical or psychological stress. At first sight, this seems in line with the “neuroprogression hypothesis” [104]. This hypothesis postulates that each affective episode in BD is neurotoxic, causing chronic (low grade) inflammation and anatomical changes, suggesting that multiple mood episodes eventually result in progressive cognitive impairment and treatment resistance [105]. However, there is currently much debate on the neuroprogression hypothesis [106], as recent longitudinal studies have reported that the decrease of cognitive function observed in BD patients is similar to the decrease in healthy controls [10, 107, 108]. An alternative explanation for the observed link between the number of mood episodes and cognitive impairment could be that cognitive impairment increases the risk of recurrences and poorer clinical course in BD [109]. Another possibility is that physical or psychological stress, for example from infections or life events, can result in chronic low‐grade inflammation, which in itself can trigger an affective episode [110]. This would imply that the course of BD does not necessarily have to be progressive and that a healthy lifestyle without physical diseases and psychological stress may reduce the risk of recurrences and progression of cognitive impairment.

CRP and Hcy are closely intertwined and possibly have synergistic effects on cardiovascular and cognitive outcomes [111]. For example, in healthy older adults at risk of cognitive decline, co‐existence of both elevated CRP and homocysteine was associated with the strongest cognitive decline throughout 5‐year follow‐up [112]. In addition, both CRP and Hcy can be influenced by mood symptoms. In the current review, significant associations between CRP, Hcy and cognitive function were observed both in studies with euthymic patients as well as in studies including patients with mood symptoms. Recently, a systematic review was published that investigated the relationship between biomarkers and cognition in BD, but specifically in different mood states of BD [113]. This review identified only two relevant studies, both of which did not find significant correlations between the biological markers and cognition. Regarding BD versus HC studies, a systematic review of observational studies reported that CRP was only elevated during mood episodes in BD [114]. A meta‐analysis on Hcy in BD concluded that Hcy levels are increased in BD patients compared to healthy controls, especially during episodes of mania [115]. In two‐month isolated mice, depressive‐like behaviors correlated with an increase of Hcy [116]. Another RCT suggested that there seems to be an inflammatory biotype of bipolar depression, which may be more susceptible to anhedonia and cognitive impairment [117]. More research is needed to unravel this knot between these biomarkers, psychiatric symptoms, and cognitive functioning.

Taken together, we hypothesize that the pathophysiology of cognitive impairment in BD is multifactorial. A vulnerability for cognitive impairment could be the result of social or psychological factors, illness severity, but also the physical health status of a BD patient. The latter may be represented by several aberrant biological marker levels. For example, vitamin deficiencies (Hcy), cardiovascular risk (Hcy), and/or presence of (low‐grade) inflammation (CRP, HSV‐1 IgG) are all risk factors for cognitive impairment, both in the general population as well as in BD patients. The presence of mood symptoms may worsen the physical condition of the patient further, which can alter these biomarker levels as well.

### 4.4 Strengths and Limitations

This systematic review is the first to provide a broad, but detailed overview of the complete spectrum of biofluid biomarkers in relation to cognition in BD. Previous reviews only focused on one biofluid biomarker or a biomarker category. Another strength was that this review combined all available evidence including studies with non‐significant findings, which reduces publication bias to some extent.

An important limitation of this review was that we did not perform a meta‐analysis or meta‐regression due to the heterogeneity of the data. The identified effect‐size measures (Table 1) were heterogeneous and often not standardized, which makes the effect sizes difficult to compare across studies. Therefore, our analysis focused on the number of studies per biomarker reporting *statistical* significance in a consistent direction. In doing so, it remains unclear if the observed effects on cognition are also *clinically* relevant. Of note, if a study reported at least one significant relationship for a biomarker, we did not list the non‐significant results of that biomarker with *other* cognitive outcomes *within* that particular study. For example, Chou et al. (2012) reported correlations of plasma BDNF with 48 (!) separate cognitive outcomes, of which only two were significant and were entered in Table 1 of this review [51].

Another limitation was that we used strict inclusion criteria due to the wide scope of the review, forcing us to exclude certain relevant studies. For example, we excluded 13 studies that were relevant, but included children, adolescents or patients from 16 or 17 years of age (Figure 1). Furthermore, we could not make definitive conclusions on causality as most studies in this review were cross‐sectional. This review did not focus on BD versus HC comparisons, as was done by a previous review [118]. Thus, we do not know whether the observed relationships between biomarkers and cognition are specific to BD (i.e., BD‐specific cognitive impairment) or may be linked to cognitive impairment in general (i.e., not BD‐specific). A recent systematic review [118] found significant overlap between biomarkers identified for BD *in general* and those associated with *cognition* in BD.

Considering the limitations of individual studies included in this review, most had small sample sizes and performed multiple testing, increasing the risk of false positive results (type I error) and false negative findings (type II error), and thus reporting and publication bias. Observed effect sizes were often small and based on unadjusted bivariate correlation analyses, so the results need to be carefully interpreted. Last, most analyses did not take important confounders into account, such as psychotropic medication use [119], physical health factors including smoking and BMI, sleep, or duration of illness.

### 4.5 Implications for Future Research

Despite decades of research, the pathophysiology of BD‐related cognitive impairment is still unclear. This impedes risk assessment for the individual BD patient and the development of personalized treatment strategies. This is problematic since cognitive impairment significantly decreases personal, functional, occupational, and social functioning [8, 9]. It is therefore essential that more research is conducted in the research area of cognitive impairment in BD.

Our hope is that in future, a multi‐dimensional comprehensive “risk score” can be developed for BD‐related cognitive impairment by integrating multiple data types, including physical status, psychiatric characteristics, (psychotropic) medication load, psychological, functional, and social factors, and ecological momentary assessments. Physical status could be represented by objective biomarkers (blood/CSF/neuroimaging) [120]. The current systematic review suggests that the biofluid biomarkers HSV‐1 IgG, CRP, and Hcy may be promising as part of such a comprehensive risk score, but the evidence is preliminary.

Due to the current risk of reporting and publication bias (see Section 3.3), future studies should replicate findings in much larger samples, which could be ensured through collaborative efforts across research centres. After this, performing a meta‐analysis or meta‐regression is essential to draw definite conclusions. In this way, the evidence can be weighted not only based on the percentage of studies reporting positive results, but also based on the magnitude of the association and total number of patients investigated across studies for a specific marker. For the biomarkers identified as “promising” by this review (HSV‐1 IgG, CRP, and Hcy), future studies also need to investigate the longitudinal course of biomarker levels in both young and older individuals with BD.

In addition, future studies should take into account possible confounding variables, such as age, sex, clinical features (e.g., bipolar subtype, mood symptoms, recurrence of episodes, suicidality), psychotropic medication use, and physical health (e.g., smoking, alcohol use, BMI, comorbid physical diseases). Perhaps, statistical analyses that deal with complexity (“everything influences everything”) such as Bayesian network analysis can be valuable in this regard. Another approach could be to investigate biomarkers for cognitive impairment across related psychiatric or physical illnesses where cognitive impairment is common (e.g., schizophrenia, ADHD, but also COVID‐19, or HIV), in order to examine whether the identified biomarkers are specific for BD‐related cognitive impairment or associated with cognitive impairment in general. Future studies could also differentiate between broad and specific cognitive domains as study outcomes.

## 5 Conclusion

This systematic review identified 184 different biofluid biomarkers that were measured in relation to cognitive functioning in BD. Of those, there was some preliminary evidence for a significant relationship between (1) HSV‐1 IgG, (2) CRP, (3) Hcy, and cognition. These biomarkers play a role in a state of (chronic low‐grade) inflammation and/or presence of increased physical or psychological stress. This indicates that a poor physical health status, which can be represented by biofluid biomarkers, may be one piece in the multifactorial puzzle that is BD‐related cognitive impairment. A key limitation was the significant publication and reporting biases, as well as small sample sizes in many of the included studies, which likely explains the inconsistent and negative findings. There is a need for larger scale studies through collaborative efforts across research centres to ensure large enough samples for robust findings.

## Funding

The authors have nothing to report.

## Conflicts of Interest

K.E.B. receives honoraria from Merck, Suven Life Sciences, and Alto Neuroscience (advisory boards) and from Breakthrough Discoveries for thriving with Bipolar Disorder (BD^2^) as Scientific Director for the Integrated Network. L.V.K. has within the recent 3 years been a consultant for Lundbeck and Teva. R.S.M. has received research grant support from CIHR/GACD/National Natural Science Foundation of China (NSFC) and the Milken Institute; speaker/consultation fees from Lundbeck, Janssen, Alkermes, Neumora Therapeutics, Boehringer Ingelheim, Sage, Biogen, Mitsubishi Tanabe, Purdue, Pfizer, Otsuka, Takeda, Neurocrine, Neurawell, Sunovion, Bausch Health, Axsome, Novo Nordisk, Kris, Sanofi, Eisai, Intra‐Cellular, NewBridge Pharmaceuticals, Viatris, Abbvie, Bristol Myers Squibb (BMS) Teva, Adhere Tech, GH Research, Autobahn Theapeutics and Atai Life Sciences. I.J.T. has served as consultant for Boehringer Ingelheim Canada. T.E.V.R. was supported by an Al and Val Rosenstrauss Fellowship form The Rebecca L Cooper Medical Research Foundation. E.V. has received grants and served as a consultant, advisor, or CME speaker for the following entities: AB‐Biotics, AbbVie, Adamed, Angelini, Biogen, Biohaven, Boehringer‐Ingelheim, Celon Pharma, Compass, Dainippon Sumitomo Pharma, Ethypharm, Ferrer, Gedeon Richter, GH Research, Glaxo‐Smith Kline, HMNC, Idorsia, Johnson & Johnson, Lundbeck, Medincell, Merck, Neuraxpharm, Newron, Novartis, Orion Corporation, Organon, Otsuka, Roche, Rovi, Sage, Sanofi‐Aventis, Sunovion, Takeda, Teva, and Viatris, outside the submitted work. L.N.Y. reports consultant/speaker fees from Alkermes, Allergan (currently Abbvie), Sumitomo Pharma, Gedeon Richter, Intracellular Therapies, Merck, Otsuka, Sanofi, and Sunovion, and grants from Allergan (now AbbVie), CIHR, and Sumitomo Pharma, outside the submitted work, over the last 3 years. A.H.Y. has the following to declare: Employed by King's College London; Honorary Consultant South London and Maudsley NHS Foundation Trust (NHS UK). Editor of Journal of Psychopharmacology and Deputy Editor, BJPsych Open. Paid lectures and advisory boards for the following companies with drugs used in affective and related disorders: Flow Neuroscience, Novartis, Roche, Janssen, Takeda, Noema pharma, Compass, Astrazenaca, Boehringer Ingelheim, Eli Lilly, LivaNova, Lundbeck, Sunovion, Servier, Livanova, Janssen, Allegan, Bionomics, Sumitomo Dainippon Pharma, Sage, Neurocentrx. Principal Investigator in the Restore‐Life VNS registry study funded by LivaNova. Principal Investigator on ESKETINTRD3004: “An Open‐label, Long‐term, Safety and Efficacy Study of Intranasal Esketamine in Treatment‐resistant Depression”. Principal Investigator on “The Effects of Psilocybin on Cognitive Function in Healthy Participants”. Principal Investigator on “The Safety and Efficacy of Psilocybin in Participants with Treatment‐Resistant Depression (P‐TRD)”. Principal Investigator on “A Double‐Blind, Randomized, Parallel‐Group Study with Quetiapine Extended Release as Comparator to Evaluate the Efficacy and Safety of Seltorexant 20 mg as Adjunctive Therapy to Antidepressants in Adult and Elderly Patients with Major Depressive Disorder with Insomnia Symptoms Who Have Responded Inadequately to Antidepressant Therapy” (Janssen). Principal Investigator on “An Open‐label, Long‐term, Safety and Efficacy Study of Aticaprant as Adjunctive Therapy in Adult and Elderly Participants with Major Depressive Disorder (MDD)” (Janssen). Principal Investigator on “A Randomized, Double‐blind, Multicentre, Parallel‐group, Placebo‐controlled Study to Evaluate the Efficacy, Safety, and Tolerability of Aticaprant 10 mg as Adjunctive Therapy in Adult Participants with Major Depressive Disorder (MDD) with Moderate‐to‐severe Anhedonia and Inadequate Response to Current Antidepressant Therapy”. Principal Investigator on “A Study of Disease Characteristics and Real‐life Standard of Care Effectiveness in Patients with Major Depressive Disorder (MDD) With Anhedonia and Inadequate Response to Current Antidepressant Therapy Including an SSRI or SNR” (Janssen). UK Chief Investigator for Compass; COMP006 and COMP007 studies. UK Chief Investigator for Novartis MDD study MIJ821A12201. Grant funding (past and present): NIMH (USA); CIHR (Canada); NARSAD (USA); Stanley Medical Research Institute (USA); MRC (UK); Wellcome Trust (UK); Royal College of Physicians (Edin); BMA (UK); UBC‐VGH Foundation (Canada); WEDC (Canada); CCS Depression Research Fund (Canada); MSFHR (Canada); NIHR (UK). Janssen (UK) EU Horizon 2020. No shareholdings in pharmaceutical companies. K.W.M. has received honoraria from Lundbeck, Gedeon Richter, and Angelini in the past 3 years. All other authors do not declare conflicts of interest.

## Supporting information


**Appendix S1:** Search Profile.

**Appendix S2:** Checklist Title/Abstract Screening.

**Appendix S3:** Instructions Full‐Text Screening.

**Appendix S4:** Appraisal Criteria For Risk Of Bias Assessment.

**Appendix S5, Table S1:** Study characteristics of all studies on biofluid biomarkers in relation to cognitive function in individuals with bipolar disorder (*n* = 60).
**Appendix S5, Table S2:** Risk of bias assessment for all of the studies included in this review (*n* = 60).

## Data Availability

The data that support the findings of this study are available from the corresponding author upon reasonable request.
