# Supplementary material for: Biofluid Biomarkers of Cognitive Functioning in Bipolar Disorder: A Systematic Review by the Targeting Cognition and Older‐Age Bipolar Disorder ISBD Task Forces
Source: Bipolar Disord. 2026 Jul 1;28(5):e70109. doi: 10.1111/bdi.70109 (PMC13324234; doi:10.1111/bdi.70109)
Supplement: Supplementary file 1 — Appendix S1: Search Profile. [file BDI-28-0-s003.docx]

**SUPPLEMENTARY MATERIAL
SEARCH PROFILE**

**Preliminary search: 05-11-2021
First definitive search: 29-12-2021
Update search: 04-07-2023**

**Total:**Pubmed (not MEDLINE) = **332 hits**EMBASE = **1399 hits**

PsycInfo = **1105 hits
Total = 2836 hits**

Duplicates removed = 712 hits
**Total after all duplicates removed = 2124 hits**

---------------------------------------------------------------------------------------------------------------------------------------
**PUBMED**

|  | Domain:patients with BD | Determinant: biomarkers | Outcome: cognitive functioning | NOT Medline | Combined | Removal of duplicates |  |
| --- | --- | --- | --- | --- | --- | --- | --- |
| MeSH | #1 | #2 | #3 |  | **#4** |  | **177 hits** |
| Ti/Ab | #5 | #6 | #7 |  | #8 |  | 895 hits |
| Ti/Ab –  NOT MEDLINE [SB] | #5 | #6 | #7 | #9 | **#10** |  | **155 hits** |
| Removal of duplicates |  |  |  |  |  | **#4 OR #10** | **332 hits** |

**Search lines
#1.** **Domain – MesH Terms**(“Bipolar Disorder”[MeSH Terms] OR “Mania”[MeSH Terms] OR “Bipolar and Related Disorders”[MeSH Terms] OR “Cyclothymic Disorder”[MesH Terms])
45,592 hits

**#2. Determinant – MesH Terms**(“Biomarkers”[MeSH Terms])
880,587 hits

**#3**. **Outcome – MesH Terms**(“Neurocognitive Disorders”[MesH Terms] OR “Cognition Disorders”[MesH Terms] OR "Cognitive Dysfunction"[MesH Terms] OR “Dementia”[MeSH Terms] OR “Frontotemporal Dementia”[MeSH Terms] OR “Dementia, Vascular”[MeSH Terms] OR “Dementia, Multi-infarct”[MeSH Terms] OR “Alzheimer Disease”[MeSH Terms] OR “Lewy Body Disease”[MeSH Terms] OR “Memory Disorders”[MeSH Terms] OR “Intellectual Disability”[Mesh Terms] OR “Perceptual Disorders”[MesH Terms] OR “Communication Disorders”[Mesh Terms] OR “Learning Disabilities”[MesH Terms] OR “Language Disorders”[Mesh Terms] OR “Cognition”[Mesh Terms] OR “Attention”[MesH Terms] OR “Memory”[MeSH Terms] OR “Learning”[MesH Terms] OR “Executive Function”[MeSH Terms] OR “Spatial Learning”[MeSH Terms] OR “Social Cognition”[MeSH Terms] OR “Social Perception”[MesH Terms] OR “Verbal Learning”[MeSH Terms] OR “Problem Solving”[MeSH Terms] OR “Psychomotor performance”[MeSH Terms] OR “Motor Skills”[MeSH Terms] OR “Mental Status and Dementia Tests”[MeSH Terms] OR “Neuropsychological Tests”[MeSH] OR “Intelligence Tests”[MesH Terms] OR “Memory and Learning Tests”[MeSH Terms] OR “Language Tests”[MeSH Terms] OR “Mental Navigation Tests”[MeSH Terms] OR “Stroop Test”[MeSH Terms] OR “Trail Making Test”[MeSH Terms] OR “Wisconsin Card Sorting Test”[MeSH Terms] OR “Maze Learning”[MeSH Terms] OR “Wechsler Memory Scale”[MeSH Terms] OR “Word Association Tests”[MesH Terms])
1,231,723 hits

**#4. Combined Domain & Determinant & Outcome (MesH Terms)**(#1 **AND** #2 **AND** #3)
177 hits

**#5. Domain – Title/abstract**(“bipolar”[Title/Abstract] OR “affective psychosis”[Title/Abstract] OR “manic”[Title/Abstract] OR “mania”[Title/Abstract] OR “manic-depressive”[Title/Abstract] OR “manic-depression”[Title/Abstract] OR “hypomania”[Title/Abstract] OR “hypomanic”[Title/Abstract] OR “(hypo)mania”[Title/Abstract] OR “(hypo)manic”[Title/Abstract] OR “manic-psychotic”[Title/Abstract] OR
“manic-psychosis”[Title/Abstract] OR “rapid cycling”[Title/Abstract])
86,267 hits

**#6. Determinant – Title/abstract**(“biomarker”[Title/Abstract] OR “marker”[Title/Abstract] OR “biosignature”[Title/Abstract] OR “CSF”[Title/Abstract] OR “cerebrospinal fluid”[Title/Abstract] OR “serum”[Title/Abstract] OR “plasma”[Title/Abstract] OR “urine”[Title/Abstract] OR “Surrogate Endpoint”[Title/Abstract] OR “Surrogate Endpoints”[Title/Abstract] OR “Surrogate End Point” [Title/Abstract] OR “Surrogate End Points”[Title/Abstract])
2,865,623 hits

**#7**. **Outcome – Title/Abstract**(“cognition”[Title/Abstract] OR “cognitive”[Title/Abstract] OR “neurocognition”[Title/Abstract] OR “neurocognitive”[Title/Abstract] OR “neuropsychological”[Title/Abstract] OR “attention”[Title/Abstract] OR “memory”[Title/Abstract] OR “verbal fluency”[Title/Abstract] OR “executive function”[Title/Abstract] OR “executive functioning”[Title/Abstract] OR “speed of processing”[Title/Abstract] OR “processing speed”[Title/Abstract] OR “visuoconstruction”[Title/Abstract] OR “visuoconstructive”[Title/Abstract] OR “visuospatial”[Title/Abstract] OR “motor speed”[Title/Abstract] OR “MMSE”[Title/Abstract] OR “Camcog”[Title/Abstract] OR “MOCA”[Title/Abstract] OR “dementia”[Title/Abstract])
1,316,347 hits

**#8. Combined Domain & Determinant & Outcome – Title/Abstract**(#5 **AND** #6 **AND** #7)
895 hits

**#9. All articles in Medline
(all of the journals available in MEDLINE can also be found in Embase)**(MEDLINE[SB])
30,577,685 hits

**#10. Combined Domain & Determinant & Outcome – Title/abstract - NOT MEDLINE**(#8 **NOT** #9)
155 hits

**#11 Removal of duplicates by both strategies:
Combined search with MesH Terms OR Combined search with Title/abstract - NOT MEDLINE**(#4 **OR** #10)
332 hits

**UPDATE SEARCH 04-07-2023:

#12 Publication date 2021 to present
(("2021/01/01"[Date - Publication] : "3000"[Date - Publication]) AND #11)
74 hits**

**EMBASE**

|  | Domain: patients with BD | Determinant: biomarkers | Outcome: cognitive functioning | Combined | Limits Human & English language & Article | Removal of duplicates |  |
| --- | --- | --- | --- | --- | --- | --- | --- |
| Map Term to Subject Heading / Explode (using Emtree terms) | #1 | #2 | #3 | **#4** |  |  | **742 hits** |
| Title/Abstract/Keywords | #5 | #6 | #7 | **#8** |  |  | 1796 hits |
| Title/Abstract/Keywords With Limits | #5 | #6 | #7 | #8 | **#9** |  | **707 hits** |
| Removal of duplicates |  |  |  |  |  | **#4 OR #9** | **1399 hits** |

**Search lines**

**#1.** **Domain – Emtree Terms**

exp bipolar disorder/ or exp mania/ or exp bipolar depression/ or exp bipolar i disorder/ or exp bipolar ii disorder/ or exp bipolar mania/ or exp cyclothymia/ or exp "mixed mania and depression"/ or exp rapid cycling bipolar disorder/
90,938 hits

**#2. Determinant – Emtree Terms**exp biological marker/
447,130 hits

**#3**. **Outcome – Emtree Terms**exp cognition/ or exp cognitive defect/ or exp dementia assessment/ or exp cognition assessment/ or exp neuropsychological test/
3,454,691 hits

**#4. Combined Domain & Determinant & Outcome (Emtree Terms)**((exp bipolar disorder/ or exp mania/ or exp bipolar depression/ or exp bipolar i disorder/ or exp bipolar ii disorder/ or exp bipolar mania/ or exp cyclothymia/ or exp "mixed mania and depression"/ or exp rapid cycling bipolar disorder/) **and** (exp biological marker/) **and** (exp cognition/ or exp cognitive defect/ or exp dementia assessment/ or exp cognition assessment/ or exp neuropsychological test/))
742 hits

**#5. Domain – Title/abstract/keywords**(bipolar or affective psychosis or manic or mania or manic-depressive or manic-depression or hypomania or hypomanic or manic-psychotic or manic-psychosis or rapid cycling).ti,ab,kf.
124,563 hits

**#6. Determinant – Title/abstract/keywords**(biomarker or marker or biosignature or CSF or cerebrospinal fluid or serum or plasma or urine or surrogate endpoint or surrogate endpoints or surrogate end point or surrogate end points).ti,ab,kf.
3,814,021 hits

**#7**. **Outcome – Title/abstract/keywords**(cognition or cognitive or neurocognition or neurocognitive or neuropsychological or attention or memory or verbal fluency or executive function or executive functioning or speed of processing or processing speed or visuoconstruction or visuoconstructive or visuospatial or motor speed or MMSE or Camcog or MOCA or dementia).ti,ab,kf.
1,733,830 hits

**#8. Combined Domain & Determinant & Outcome – Title/abstract/keywords**

((bipolar or affective psychosis or manic or mania or manic-depressive or manic-depression or hypomania or hypomanic or manic-psychotic or manic-psychosis or rapid cycling) **and** (biomarker or marker or biosignature or CSF or cerebrospinal fluid or serum or plasma or urine or surrogate endpoint or surrogate endpoints or surrogate end point or surrogate end points) **and** (cognition or cognitive or neurocognition or neurocognitive or neuropsychological or attention or memory or verbal fluency or executive function or executive functioning or speed of processing or processing speed or visuoconstruction or visuoconstructive or visuospatial or motor speed or MMSE or Camcog or MOCA or dementia)).ti,ab,kf.
1796 hits

**#9. Search #8 with Embase Limits: only humans, only English language and only articles**

limit 8 to (human and English language and article)
707 hits

**#10 Removal of duplicates by both strategies:
Combined Emtree search OR Combined search with Title/abstract/keywords**(4 or 9) **1399** hits

**UPDATE SEARCH 04-07-2023:
Publication year 2021 to current**
limit 10 to yr="2021 -Current"
**349 hits**

**APA PSYCINFO**

|  | Domain:patients with BD | Determinant: biomarkers | Outcome: cognitive functioning | Combined | Removal of duplicates |  |
| --- | --- | --- | --- | --- | --- | --- |
| Psychological Index Terms | #1 | #2 | #3 | **#4** |  | 54 hits |
| TI/AB/KW | #5 | #6 | #7 | **#8** |  | 1095 hits |
| Removal of duplicates |  |  |  |  | **#4 OR #8** | **1105** **hits** |

**Search lines
#1.** **Domain – Psychological Index Terms**(DE “Bipolar Disorder” OR DE “Bipolar I Disorder” OR DE “Bipolar II Disorder” OR DE “Cyclothymic Disorder” OR DE “Mania”)
40,862 hits

**#2. Determinant – Psychological Index Terms**(DE “Biological Markers”)
18,066 hits

**#3**. **Outcome – Psychological Index Terms**(DE “Cognitive Ability” OR DE “Cognitive Impairment” OR DE “Cognitive Assessment” OR DE “Neuropsychological Assessment” OR DE “Neurocognitive Disorders” OR DE “Cognitive Processes” OR DE “Cognition” OR DE “Concentration” OR DE “Attention” OR DE “Memory” OR DE “Learning” OR DE “Executive Function” OR DE “Cognitive Flexibility” OR DE “Neurocognitive Disorders” OR DE “Dementia” OR DE “Social Cognition” OR DE “Perception” OR DE “Cognitive Control” OR DE “Intelligence Quotient” OR DE “Language Disorders” OR DE “Learning Disorders” OR DE "Memory and Learning Measures” OR DE “Perceptual Motor Processes” OR DE “Perceptual Measures”)

592,455 hits

**#4. Combined Domain & Determinant & Outcome (Psychological Index Terms)**
(DE “Bipolar Disorder” OR DE “Bipolar I Disorder” OR DE “Bipolar II Disorder” OR DE “Cyclothymic Disorder” OR DE “Mania”) **AND** (DE “Biological Markers”) **AND** (DE “Cognitive Ability” OR DE “Cognitive Impairment” OR DE “Cognitive Assessment” OR DE “Neuropsychological Assessment” OR DE “Neurocognitive Disorders” OR DE “Cognitive Processes” OR DE “Cognition” OR DE “Concentration” OR DE “Attention” OR DE “Memory” OR DE “Learning” OR DE “Executive Function” OR DE “Cognitive Flexibility” OR DE “Neurocognitive Disorders” OR DE “Dementia” OR DE “Social Cognition” OR DE “Perception” OR DE “Cognitive Control” OR DE “Intelligence Quotient” OR DE “Language Disorders” OR DE “Learning Disorders” OR DE “Memory and Learning Measures” OR DE “Perceptual Motor Processes” OR DE ”Perceptual Measures”)

54 hits

**#5. Domain – Title/abstract/keywords
TI** ( bipolar or “affective psychosis” or manic or mania or manic-depressive or manic-depression or hypomania or hypomanic or (hypo)mania or (hypo)manic or manic-psychotic or “manic-psychosis” or “rapid cycling” ) **OR AB** ( bipolar or “affective psychosis” or manic or mania or manic-depressive or manic-depression or hypomania or hypomanic or (hypo)mania or (hypo)manic or manic-psychotic or “manic-psychosis” or “rapid cycling” ) **OR KW** ( bipolar or “affective psychosis” or manic or mania or manic-depressive or manic-depression or hypomania or hypomanic or (hypo)mania or (hypo)manic or manic-psychotic or “manic-psychosis” or “rapid cycling” )
57,786 hits

**#6. Determinant – Title/abstract/keywords
TI** ( biomarker or marker or biosignature or CSF or “cerebrospinal fluid” or serum or plasma or urine or “surrogate endpoint” or “surrogate endpoints” or “surrogate end point” or “surrogate end points” ) **OR AB** ( biomarker or marker or biosignature or CSF or “cerebrospinal fluid” or serum or plasma or urine or “surrogate endpoint” or “surrogate endpoints” or “surrogate end point” or “surrogate end points” ) **OR KW** ( biomarker or marker or biosignature or CSF or “cerebrospinal fluid” or serum or plasma or urine or “surrogate endpoint” or “surrogate endpoints” or “surrogate end point” or “surrogate end points” )
146,816 hits

**#7**. **Outcome – Title/abstract/keywords
TI** ( cogniti* or neurocogniti* or neuropsychological or attention or memory or “verbal fluency” or “executive function” or “executive functioning” or “speed of processing” or “processing speed” or visuoconstruction or visuoconstructive or visuospatial or “motor speed” or MMSE or camcog or MOCA or dementia ) **OR AB** ( cogniti* or neurocogniti* or neuropsychological or attention or memory or “verbal fluency” or “executive function” or “executive functioning” or “speed of processing” or “processing speed” or visuoconstruction or visuoconstructive or visuospatial or “motor speed” or MMSE or camcog or MOCA or dementia ) **OR KW** ( cogniti* or neurocogniti* or neuropsychological or attention or memory or “verbal fluency” or “executive function” or “executive functioning” or “speed of processing” or “processing speed” or visuoconstruction or visuoconstructive or visuospatial or “motor speed” or MMSE or camcog or MOCA or dementia )
1,007,061 hits

**#8. Combined Domain & Determinant & Outcome – Title/abstract/keywords**( **TI** ( bipolar or “affective psychosis” or manic or mania or manic-depressive or manic-depression or hypomania or hypomanic or (hypo)mania or (hypo)manic or manic-psychotic or “manic-psychosis” or “rapid cycling” ) **OR AB** ( bipolar or “affective psychosis” or manic or mania or manic-depressive or manic-depression or hypomania or hypomanic or (hypo)mania or (hypo)manic or manic-psychotic or “manic-psychosis” or “rapid cycling” ) **OR KW** ( bipolar or “affective psychosis” or manic or mania or manic-depressive or manic-depression or hypomania or hypomanic or (hypo)mania or (hypo)manic or manic-psychotic or “manic-psychosis” or “rapid cycling” ) ) **AND** ( **TI** (biomarker or marker or biosignature or CSF or “cerebrospinal fluid” or serum or plasma or urine or “surrogate endpoint” or “surrogate endpoints” or “surrogate end point” or “surrogate end points”) **OR AB** (biomarker or marker or biosignature or CSF or “cerebrospinal fluid” or serum or plasma or urine or “surrogate endpoint” or “surrogate endpoints” or “surrogate end point” or “surrogate end points”) **OR KW** (biomarker or marker or biosignature or CSF or “cerebrospinal fluid” or serum or plasma or urine or “surrogate endpoint” or “surrogate endpoints” or “surrogate end point” or “surrogate end points”) ) **AND** ( **TI** ( cogniti* or neurocogniti* or neuropsychological or attention or memory or “verbal fluency” or “executive function” or “executive functioning” or “speed of processing” or “processing speed” or visuoconstruction or visuoconstructive or visuospatial or “motor speed” or MMSE or camcog or MOCA or dementia ) **OR AB** ( cogniti* or neurocogniti* or neuropsychological or attention or memory or “verbal fluency” or “executive function” or “executive functioning” or “speed of processing” or “processing speed” or visuoconstruction or visuoconstructive or visuospatial or “motor speed” or MMSE or camcog or MOCA or dementia ) **OR KW** ( cogniti* or neurocogniti* or neuropsychological or attention or memory or “verbal fluency” or “executive function” or “executive functioning” or “speed of processing” or “processing speed” or visuoconstruction or visuoconstructive or visuospatial or “motor speed” or MMSE or camcog or MOCA or dementia ) )

1095 hits

**#9 Removal of duplicates by both strategies:
Combined search with Psychological Index Terms OR Combined search with TI/AB/KW**(S4 OR S8) 
1105 hits

**UPDATE SEARCH 04-07-2023:
Limiters** - Publication Year: 2021-2023
**168 hits**
