# Supplementary material for: Biofluid Biomarkers of Cognitive Functioning in Bipolar Disorder: A Systematic Review by the Targeting Cognition and Older‐Age Bipolar Disorder ISBD Task Forces
Source: Bipolar Disord. 2026 Jul 1;28(5):e70109. doi: 10.1111/bdi.70109 (PMC13324234; doi:10.1111/bdi.70109)
Supplement: Supplementary file 2 — Appendix S2: Checklist Title/Abstract Screening. [file BDI-28-0-s002.docx]

**CHECKLIST TITLE/ABSTRACT SCREENING**

**GENERAL**Inclusion:
All original research study designs (cross-sectional, case-control, cohort, randomized trials, quasi-experimental, etc.)
Exclusion:
1. If no original research article (review, systematic review, editorial, letter, etc) **🡪 Exclude**

2. If animal study (and no humans also involved!) **🡪 Exclude**

3. If in vitro/laboratory studies (and no humans also involved!) **🡪 Exclude**4. Unpublished other literature sources (e.g. Msc thesis, dissertation) **🡪 Exclude

DOMAIN**Inclusion:
-individuals with diagnosis of bipolar disorder (BD-I, BD-II, BD-NOS)
-mean age of patients ≥18 yrs
-all symptom states are valid (so euthymic and symptomatic, outpatient and inpatients)
Exclusion:
1. If no BD patients at all **🡪 Exclude**BUT: if mixed sample (BD + other diagnoses) **🡪 Include for now, in full text we will check if there is separate reporting for the BD group**

2. If only BD children or adolescents <18 yrs **🡪 Exclude**BUT: if mixed sample (children + adults) **🡪 Include for now, in full text we will check if there is separate reporting for the adult group**3. Serial case study with <5 BD patients **🡪 Exclude**
**DETERMINANTS**Inclusion:
Measurement of at least one biofluid biomarker
Exclusion:
1. No biofluid biomarker measured at all in the study **🡪 Exclude**(all kinds of biofluids, so include saliva, plasma, serum, urine, cerebrospinal fluid/CSF, etc)

2. Only DNA/RNA/ genetic marker assessed **🡪 Exclude**
(e.g. polymorphisms such as APO-E or BDNF alleles, mRNA in blood, candidate genes, chromosome regions, GWAS studies or other genetic analyses)

3. Only structural or functional imaging biomarkers marker assessed **🡪 Exclude**(e.g. neuroanatomical markers, fMRI, CT, PET, SPECT, EEG, measurement of cerebral blood flow, diffusion tensor imaging, etc.)
3. Only medication or add-on measured in biofluid measured **🡪 Exclude**(e.g. lithium/ valproate in serum, ketamine/ other drug in serum)
Thus: biomarker should be naturally present in the biofluid!

4. Studies that only investigate a biomarker in bipolar disorder vs. healthy controls without any cognitive outcome **🡪 Exclude**

NB. We exclude articles that focus on the relationship between specific diseases or health conditions and cognition in BD; for example, we include studies that measured triiodothyronine (T3) and glucose as biomarkers, but exclude studies on hyper- or hypothyroidism and diabetes in relation to cognition.

**OUTCOME**Inclusion:
- include studies that assess a cognitive screener such as FAB, MMSE. MocA, CamCog.
- include studies that assess a continuous outcome for cognitive performance
(e.g. composite score for a cognitive domain, a neuropsychological test score, a composite score for overall cognitive functioning an average Z-score or g-score from a full extensive neurocognitive assessment)
- include studies that assess a dichotomous outcome for cognition
(e.g. different groups of BD patients categorized by cognitive functioning: BD-no cognitive impairment, BD-MCI, BD-severe neurocognitive disorder/dementia)

🡪 sometimes, the abstract only mentions something vague like “biomarker X was (not) related to cognition”. If there is any suspicion that any cognitive measure has been assessed **🡪 Include for now, in full text we will check thoroughly which cognitive measure is assessed.**

Exclusion:
1. No measurement of cognition in abstract at all **🡪 Exclude**

2. Studies that only measure IQ, general intelligence, or general intellectual ability **🡪 Exclude**

**PLEASE NOTE!**-In the full text screening, we will check much more thoroughly if all of the above criteria are met.
-In addition, we will check if the article assesses the relationship between the biofluid biomarker and the cognitive measure.

So: if the abstract states that any biofluid biomarker is assessed + any cognitive measure + in BD patients… **🡪 Include, even if you actually think that the design is probably not correct!**
(e.g. in an RCT, there can still be an analysis between the biofluid biomarkers and cognition in BD patients at baseline somewhere in the supplementary data!)
