# Supplementary material for: Biofluid Biomarkers of Cognitive Functioning in Bipolar Disorder: A Systematic Review by the Targeting Cognition and Older‐Age Bipolar Disorder ISBD Task Forces
Source: Bipolar Disord. 2026 Jul 1;28(5):e70109. doi: 10.1111/bdi.70109 (PMC13324234; doi:10.1111/bdi.70109)
Supplement: Supplementary file 3 — Appendix S3: Instructions Full‐Text Screening. [file BDI-28-0-s001.docx]

**INSTRUCTIONS FULL-TEXT SCREENING**
 **C. DOMAIN**Inclusion:
-individuals with diagnosis of bipolar disorder (BD-I, BD-II, BD-NOS)
-age of patients ≥18 years old
-all symptom states are valid (so euthymic and symptomatic, outpatient and inpatients)
Exclusion:
1. If no BD patients at all **🡪 Exclude**
2. If serial case study with <5 BD patients **🡪 Exclude**

3. If combined sample with both BD children or adolescents <18 yrs and adults >18 yrs:
check if there is separate reporting for the adult >18 yrs BD group
**🡪 if no separate reporting for the adult >18 yrs BD group 🡪 Exclude**
4. If mixed sample (BD + other diagnoses): check if there is separate reporting for the BD group **🡪 if no separate reporting for the BD group 🡪 Exclude**

**D. DETERMINANTS**Inclusion:
Measurement of at least one biofluid biomarker
(all kinds of biofluids, so include saliva, plasma, serum, urine, cerebrospinal fluid/CSF, etc)
Exclusion:
1. No biofluid biomarker measured at all in the study **🡪 Exclude**
2. Biomarker should be naturally present in the biofluid!
So, if only medication or add-on measured in biofluid measured (e.g. lithium/ valproate in serum, ketamine/ other drug in serum) **🡪 this is no biomarker! 🡪 Exclude**3. The only biomarker or biomarkers assessed are DNA/RNA/ genetic marker(s)**🡪 Exclude**
(e.g. polymorphisms such as APO-E or BDNF alleles, mRNA in blood, candidate genes, chromosome regions, GWAS studies or other genetic analyses)

4. The only biomarker or biomarkers assessed are structural or functional imaging biomarkers marker(s)**🡪 Exclude**(e.g. neuroanatomical markers, fMRI, CT, PET, SPECT, EEG, measurement of cerebral blood flow, diffusion tensor imaging, etc.)

NB. We exclude articles that focus on the relationship between specific diseases or health conditions and cognition in BD; for example, we include studies that measured triiodothyronine (T3) and glucose as biomarkers, but exclude studies on hyper- or hypothyroidism and diabetes in relation to cognition.

**E. OUTCOME**Inclusion:
- include studies that assess an objective **continuous outcome** for cognitive performance
(e.g. composite score for a cognitive domain, a neuropsychological test score, a composite score for overall cognitive functioning an average Z-score or g-score from a full extensive neurocognitive assessment)
- include studies that assess an objective **dichotomous outcome** for cognition
(e.g. different groups of BD patients categorized by cognitive functioning:
BD-no cognitive impairment, BD-MCI, BD-dementia)

Exclusion:
1. No objective measurement of cognition at all (e.g. self-report questionnaire) **🡪 Exclude**

2. Studies that ONLY measure IQ, general intelligence, or general intellectual ability
**🡪 Exclude**3. Studies that ONLY assess an objective **cognitive screener** such as FAB, MMSE, MocA, CamCog, no cognitive test. **🡪 Exclude**

4. Studies that measure cognition, but cognitive function is altered the cognitive outcome in some way (e.g. RCT with medication, cognition is only measured at the end of the treatment period). **🡪 Exclude**
(RCTs that measure the baseline relationship between biomarker & cognition are allowed.) **F. STATISTICS**Inclusion:
-The research article should have measured a direct association between the biomarker and the cognitive outcome: the biomarker is a predictor variable, covariate or adjusted for in the statistical models with outcome of objectively measured cognitive functioning or cognitive impairment.
-If the study uses a dichotomous outcome of cognitive impairment, or dementia: a comparator or control groups is necessary. For example, biomarker level is compared between BD patients without cognitive dysfunction vs. BD-MCI vs. BD-dementia.
-If the study uses a continuous outcome for neurocognitive functioning (e.g. neuropsychological test score or Z-score), the study does not have to include a comparator or control group.

**LOOK THOROUGHLY THROUGH THE FULL TEXT, ALSO THROUGH THE SUPPLEMENTAL DATA! SOMETIMES A RELATIONSHIP IS REPORTED IN THE TEXT BUT NOT IN THE TABLES.**(e.g. sometimes in an RCT, there can still be a baseline analysis between the biofluid biomarkers and cognition in BD patients! These are often listed in the supplementary data!)

Exclusion examples:
1. Studies that only investigate a biomarker in bipolar disorder vs. healthy controls without any cognitive outcome **🡪 Exclude**

2. If a relationship between biomarker and cognition is measured, but only for a mixed sample without separate reporting
🡪 **Exclude, main reason for exclusion is mixed sample without separate reporting.**
