# Supplementary material for: Biofluid Biomarkers of Cognitive Functioning in Bipolar Disorder: A Systematic Review by the Targeting Cognition and Older‐Age Bipolar Disorder ISBD Task Forces
Source: Bipolar Disord. 2026 Jul 1;28(5):e70109. doi: 10.1111/bdi.70109 (PMC13324234; doi:10.1111/bdi.70109)
Supplement: Supplementary file 4 — Appendix S4: Appraisal Criteria For Risk Of Bias Assessment. [file BDI-28-0-s005.docx]

**APPRAISAL CRITERIA FOR RISK OF BIAS ASSESSMENT**

The criteria were adapted from The Joanna Briggs Institute Critical Appraisal tools for use in JBI Systematic Reviews; Checklist for Analytical Cross Sectional Studies
<http://joannabriggs.org/research/critical-appraisal-tools.html><https://jbi.global/sites/default/files/2019-05/JBI_Critical_Appraisal-Checklist_for_Analytical_Cross_Sectional_Studies2017_0.pdf>
Each criterium was scored on presence of ‘good quality’(Yes) or not (No).

**1. Were the criteria for inclusion in the sample clearly defined? (selection bias)**
The authors should provide clear inclusion and exclusion criteria that they developed prior to recruitment of the study participants. The inclusion/exclusion criteria should be specified (e.g., risk, stage of disease progression) with sufficient detail and all the necessary information critical to the study.
**→ Good quality if:
inclusion and exclusion criteria are clearly specified**

**2. Were the study subjects and the setting described in detail? (selection bias)**The study sample should be described in sufficient detail so that other researchers can determine if it is comparable to the population of interest to them. The authors should provide a clear description of the population from which the study participants were selected or recruited, including demographics, location, and time period.
**→ Good quality if:
there is a description of the patient group and setting (age, education level, duration of illness, use of psychotropic medication, inpatient/outpatients, current mood symptoms)**
**3. Was the exposure measured in a valid and reliable way? (information bias)**The study should clearly describe the method of measurement of exposure. Assessing validity requires that a 'gold standard' is available to which the measure can be compared. The validity of exposure measurement usually relates to whether a current measure is appropriate or whether a measure of past exposure is needed. Reliability refers to the processes included in an epidemiological study to check repeatability of measurements of the exposures. These usually include intra-observer reliability and interobserver reliability.
**→ Good quality if:
-biomarkers were measured according to a standardized study protocol. If the biomarker data were drawn from the electronic patient file, this is insufficient!
-the biomarker should preferably be measured at the same day (or at least in the same week) as the neurocognitive assessment**
**4. Were objective, standard criteria used for measurement of the condition? (selection bias)**It is useful to determine if patients were included in the study based on either a specified diagnosis or definition. This is more likely to decrease the risk of bias. Characteristics are another useful approach to matching groups, and studies that did not use specified diagnostic methods or definitions should provide evidence on matching by key characteristics.
→ **Good quality if:
bipolar disorder was diagnosed with a structured diagnostic method (e.g. SCID/ MINI). Expert clinical opinion alone is insufficient.**
**5. Were confounding factors identified? (Not Applicable)**
Confounding has occurred where the estimated intervention exposure effect is biased by the presence of some difference between the comparison groups (apart from the exposure investigated/of interest). Typical confounders include baseline characteristics, prognostic factors, or concomitant exposures (e.g. smoking). A confounder is a difference between the comparison groups and it influences the direction of the study results. A high quality study at the level of cohort design will identify the potential confounders and measure them (where possible). This is difficult for studies where behavioral, attitudinal or lifestyle factors may impact on the results.
**→ This criterion is not applicable for our studies, as the analyses of interest are WITHIN the BD group. So it is not necessary to have an intervention or comparison group. Also, most studies are cross-sectional and do not have a cohort design.

6. Were strategies to deal with confounding factors stated? (Confounding)**
Strategies to deal with effects of confounding factors may be dealt with within the study design or in data analysis. By matching or stratifying sampling of participants, effects of confounding factors can be adjusted for. When dealing with adjustment in data analysis, assess the statistics used in the study. Most will be some form of multivariate regression analysis to account for the confounding factors measured.
**→ Good quality if:
the study describes strategies to deal with confounders.**

**7. Were the outcomes measured in a valid and reliable way? (Information bias)**
Read the methods section of the paper. If for e.g. lung cancer is assessed based on existing definitions or diagnostic criteria, then the answer to this question is likely to be yes. If lung cancer is assessed using observer reported, or self-reported scales, the risk of over- or underreporting is increased, and objectivity is compromised. Importantly, determine if the measurement tools used were validated instruments as this has a significant impact on outcome assessment validity. Having established the objectivity of the outcome measurement (e.g. lung cancer) instrument, it’s important to establish how the measurement was conducted. Were those involved in collecting data trained or educated in the use of the instrument/s? (e.g. radiographers). If there was more than one data collector, were they similar in terms of level of education, clinical or research experience, or level of responsibility in the piece of research being appraised?
**→ Good quality if:**
**-the study used several validated neuropsychological tests (at least three)
-the study assessed several cognitive domains (at least two)
-at least two NP (subitem) scores per cognitive domain. Several scores within one test are allowed (e.g. RAVLT total learning and RAVLT delayed recall)
-the neuropsychological test scores were standardized (either compared to HC data or to normative data, controlling for age/education level/sex).
-If no standardization: comparison of cognitive data between BD and HC group.**

**8. Was appropriate statistical analysis used? (Confounding/ Information bias)**
As with any consideration of statistical analysis, consideration should be given to whether there was a more appropriate alternate statistical method that could have been used. The methods section should be detailed enough for reviewers to identify which analytical techniques were used (in particular, regression or stratification) and how specific confounders were measured. For studies utilizing regression analysis, it is useful to identify if the study identified which variables were included and how they related to the outcome. If stratification was the analytical approach used, were the strata of analysis defined by the specified variables? Additionally, it is also important to assess the appropriateness of the analytical strategy in terms of the assumptions associated with the approach as differing methods of analysis are based on differing assumptions about the data and how it will respond.
**→ Good quality if:
statistical analysis was adjusted for confounders (e.g. multiple regression, ANCOVA, MANCOVA) AND cognitive measure was used as the outcome AND biomarker as the determinant
- insufficient quality in case of simple, bivariate correlation without correction for multiple testing**

**EXTRA:
9. Does the sample size seem sufficient? (Reporting and Publication bias)**

**→ Good quality if:
-sample size ≥100 BD patients**


**CONCLUSION OVERALL QUALITY APPRAISAL:
-in case of 0x ‘No’ 🡪 GOOD quality
-in case of 1x or 2x ‘No’ 🡪 FAIR quality
-in case of 3x ‘No’ or more → POOR quality
-in case of doubt: discuss in research team**
