# Supplementary material for: Biofluid Biomarkers of Cognitive Functioning in Bipolar Disorder: A Systematic Review by the Targeting Cognition and Older‐Age Bipolar Disorder ISBD Task Forces
Source: Bipolar Disord. 2026 Jul 1;28(5):e70109. doi: 10.1111/bdi.70109 (PMC13324234; doi:10.1111/bdi.70109)
Supplement: Supplementary file 5 — Appendix S5, Table S1: Study characteristics of all studies on biofluid biomarkers in relation to cognitive function in individuals with bipolar disorder (n = 60). Appendix S5, Table S2: Risk of bias assessment for all of the studies included in this review (n = 60). [file BDI-28-0-s004.zip › bdi70109-sup-0005-TableS1.docx]

**SUPPLEMENTAL DATA**

**TABLE S1. Study characteristics of all studies on biofluid biomarkers in relation to cognitive function in individuals with bipolar disorder (n=60)**

| **Study** First author (year)  *journal* | **Study design** | **Sample**  n for BD  % BD-I/ BD-II  (important inclusion criteria, inpatients/ outpatients, mood state)  n for HC (matching criteria) | **Gender in BD group**  (% if reported) | **Age in BD group** in years, M (SD) | **Education level** **in BD group**  in years,  M (SD) | **Duration of illness**  in years, M (SD) | **Psychotropic medication use**  (% if reported) | **Type of biofluid and biomarker(s)** (See Table 2 for the full names of the abbreviated biomarkers) | **Cognitive outcome measures**  (Only cognitive domains and neuropsychological tests are stated. Additional IQ measures and cognitive screeners (e.g. MMSE, MocA) are not mentioned. Cognitive domains are exactly as mentioned in each study, so sometimes the same neuropsychological test will fall under different cognitive domains across studies.) | **Statistical analyses**  (e.g. regression, correlation) | **Correction**  (e.g. for multiple comparisons or covariates) | **Study Quality** (conclusion from risk of bias assessment) |
| --- | --- | --- | --- | --- | --- | --- | --- | --- | --- | --- | --- | --- |
| **Aydemir et al. (2014)** *Revista Brasileira de Psiquiatria/* *Brazilian Journal of Psychiatry* | cross-sectional | **51 BD** 64.7% BD-I 35.3% BD-II  (in remission for >= 6 months)  **50 HC** | 24 F 27 M | 40.8 (SD 11.5) | 12.1  (SD 3.6) | 14.1 (SD 9.1) | Yes (100%) | Serum: -SOD  -NO -NT-4 -Hcy -folic acid  -vit B12  Plasma: -MDA  Whole blood: -GSH | ***Attention:** -Continuous Performance Test ***Executive functioning & working memory:** -Wisconsin Card Sorting Test  -Stroop Test  -Trail Making Test B  ***Processing speed:** -Trail Making Test A ***Verbal learning & memory:**  -Rey Auditory Verbal Learning Test -Serial Digit Learning Test ***Visuospatial function:** -Judgment of Line Orientation Test | Partial correlation | -duration of illness  -number of  episodes | **POOR** |
| **Barbosa et al. (2018)** *Journal of Psychiatric Research* | cross-sectional | **20 BD-I** (outpatients, in remission)   **25 HC** (age + gender matched) | 15 F 5 M | 43.80 (SD 10.87) | 12.40 (SD 3.39) | 20.15 (SD 12.13) | Yes | Plasma: -IL-2 -IL-4 -IL-6 -IL-10 -IFN-γ -TNFα -IL-17A -sTNF-αR1 -sTNF-αR2 | Global cognitive performance index, based on 8 tests from the Brief Assessment of Cognition in Affective Disorders (BAC-A): -Affective processing test  -List Learning  -Digit Sequencing task  -Token Motor task  -Verbal fluency, category instances  -Controlled Oral Word Association test (COWAT)  -Tower of London test  -Symbol coding | -Pearson’s correlation  -Multiple regression analysis with backward elimination procedure  (outcome BAC-A global performance, predictors duration of disease, years of study, MMSE, and IL6 (variables p < 0.05 on univariate analyses) + change in cognitive measurement (?). | final multiple regression  model with IL-6: -MMSE -years of study (-illness duration?) | **FAIR** |
| **Bell et al. (1990)** *Journal of Geriatric Psychiatry and Neurology* | Cross-sectional analysis, retro- spective study | **8 BD** (geriatric inpatients with psychotic depression) | 2 M 6 F | 77.0 (SD 7.4) | -12.5% (n=1)  < high school -37.5% (n=3) high school -37.5% (n=3) college -12.5% (n=1) post-college | ? | ? | Serum: -vit B12 -folate | -Wechsler Memory Scale (WMS) (Mental Control, immediate and delayed Logical Memory, Visual Reproduction, Associate Learning subtests) -Boston Naming Test (BNT) -Controlled Oral Word Association (COWAT: F,A,S). | -Pearson correlation  coefficients | Correction for multiple comparisons:  significance level of p<0.01. | **POOR** |
| **Chen et al. (2019)**  *International Journal of Geriatric Psychiatry* | cross-sectional | **95 BD-I**  (outpatients, aged >= 55 yrs)  subgroups n=50 LOBD: (age of onset >=40 yrs) and n=45 EOBD | 51 M 44 F | EOBD: 62.3 (SD 3.7)  LOBD: 63.8 (SD 5.9) | EOBD: 11.3 (SD 4.1)  LOBD: 9.9 (SD 4.1) | EOBD: 38.4  (SD 9.1)  LOBD: 11.3 (SD 8.1) | Yes | Serum: -Hcy -vit B12 -folate -T3 | -total immediate recall, delayed recall, and recognition from word list subtest of WMS‐III -CTT‐1 completion time and CTT‐2 completion time from CTTs -block design score from the WAIS‐III -verbal fluency score from the fruit naming test -digit symbol substitution test score from WAIS‐III | multiple regression analyses with stepwise procedure, variables with p<0.1 in preliminary analyses were selected.  Main analysis between EOBD/ LOBD and cognition | -age -gender - years of education  -duration of illness -10‐year Framingham cardiovascular risk scores -Hcy -T3 (biomarkers as covariates!) | **POOR** |
| **Chen et al. (2022)**  *The British Journal of Psychiatry Open (BJPsych Open)* | cross-sectional | **100 BD-I** (euthymic outpatients from a tertiary psychiatric  hospital)  subgroups  age 20-45 and 45-65 years | 48 M 52 F | 47.49  (SD 11.71) | age 20-45, vit D normal: N=16: 13.63 (2.35) age 20-45, vit D deficient: N=27: 14.00 (2.17) age 45-65, vit D normal: N=12 13.17 (3.43) age 45-65, vit D deficient N=45 12.37 (3.51) | ?  (age of onset: 24.78 years, SD 9.79) | Yes (100%) | Serum: -25(OH)D -NfL | Brief Assessment of Cognition in Affective Disorders (BAC-A): ***affective memory**  (Affective Auditory Verbal Learning Test) ***emotional inhibition:** (Emotional Stroop Task) ***working memory**  (Digit Sequencing Task) ***motor speed** (Token Motor Task) ***verbal fluency**  (Category Instances and Controlled Oral Word Association Test) ***processing speed** (Symbol Coding) ***verbal memory** (List Learning) ***executive function** (Tower of London)  **⇒ composite score** | -General linear models  -interaction plot representing the interaction effect of vitamin D and NfL  levels on cognition in each age group (20-45 yrs and 46-65 yrs) | *Model 1: age, gender, vitamin D level, NfL level and interaction of vitamin D and NfLs. *Model 2: age, gender, defined daily dose of total psychotropic medications, years of education, number  hospital admissions, level of physical activity determined with the International Physical Activity Questionnaire, vitamin D level, NfL level and interaction of vitamin D and NfLs. | **FAIR** |
| **Chou et al. (2012)** *Journal of Affective Disorders* | cross-sectional | **23 BD-I** (at least 1 manic episode in past 2 yrs + currently stable treatment + euthymic, MADRS<10 and YMRS<7)   **33 HC** (age matched) | 6 M 17 F | 36.5 (SD 8.9) | 13.1 (SD 2.7) | 86.5 months (SD 56.2) | Yes  (but no medication on test day. Past use of anti-depressants, if any, had been discontinued for at least 1 year) | Plasma: BDNF | ***Attention:** Test for Attentional Performance (TAP): -GO/NO-GO task: 4 subscores -divided attention task: 8 subscores ***Memory**  -WMS-III: Word Lists Test (verbal memory function): 9 subscores -WMS-III: Face Test (visual memory function): 7 subscores ***Executive function:** -Color Trails Test (CTT): 10 subscores -Wisconsin Card Sorting Test (WCST): 10 subscores | partial correlations | age, gender, education | **FAIR** |
| **Civil Arslan et al. (2017)** *Türk Psikiyatri Dergisi/* *Turkish Journal of Psychiatry* | cross-sectional | **36 BD-I**  (outpatients, euthymic for 6 months)  **38 HC** (age, sex, and education level matched) | 22 F (61.1%) 14 M (38.9%) | 38 (SD 11.80) | 10.88 (SD 3.63) | ? | Yes  Lithium n=27 (75%)  Anticonvulsant n=17 (47.2%)  Antipsychotic n=31 (86.1%) | Serum: -IL-6 -IL-18 | -Wisconsin Card Sorting Test (WCST) -Stroop BSRG (Basic Sciences Research Group): a combination of the original Stroop test (Stroop 1935) and the Victoria Form (Spreen and Strauss 1998). -Rey Auditory Verbal Learning Test (RAVLT): learning (RAVLT 1-5), recognition, instant recall (RAVLT 1), and delayed recall (RAVLT-7). | -Pearson’s correlation -Spearman’s correlation | No | **POOR** |
| **Dickerson et al. (2004)** *Biological Psychiatry*  **sample overlap with Dickerson et al. (2006, 2013, 2014)** | cross-sectional | **117 BD**  87 BD-I (74.4%) 29 BD-II (24.8%) 1 BD-NOS (1%)  (outpatients) **100 HC** | 35 M (30%), 82 F (70%) | 41.4 (SD 12.2) range  18–64 | 14.3  (SD 2.9) | 21.6  (SD  12.5) | Yes (98%) | Serum: IgG antibodies for six herpesviruses: HSV-1 HSV-2 CMV EBV HHV-6  VZV | Repeatable Battery for the Assessment of Neuropsychological Status (RBANS) with 12 subtests for five index scores + total score ***Immediate Memory** -List Learning -Story Memory ***Visuospatial/Constructional** -Figure Copy -Line Orientation ***Language** -Picture Naming -Semantic Fluency ***Attention** -Digit Span -Coding ***Delayed Memory** -List Recall -Story Recall -Figure Recall -List Recognition **⇒ total score** | *univariate ANOVA: comparison RBANS for BD patients with and without seropositivity for each virus   *MANCOVA for HSV-1  *for HSV-1: multivariate forward stepwise  linear discriminant function analysis | *ANOVA: p level = .008  *final multivariate ANCOVA model: -years of education -PANSS Negative Symptom  score -current treatment with an  antipsychotic | **GOOD** |
| **Dickerson et al. (2006)**  *Bipolar Disorders*  **sample overlap with Dickerson et al. (2004, 2013, 2014)** | cross-sectional | **107 BD**  (outpatients)  **95 HC** | 33 M (31%) 91 F (85%) | 41.7 (SD 12.2). | 14.3 (SD 2.9) | 21.7  (SD 12.7) | Yes (96%) | serum: HSV-1 IgG | -Repeatable Battery for the Assessment of Neuropsychological status (RBANS, Form A):  total score + 5 index scores Immediate Memory, Visuospatial/ Constructional Abilities, Language, Attention, Delayed Memory. -WAIS-III Letter-Number Sequencing Subtest (LNST) | *MANCOVA with correction for multiple comparisons (p=0.01)  *maximum likelihood multinomial  logistic regression analyses to compute the  relative risk of various combinations of COMT  genotype and HSV-1 seropositivity for quintile  RBANS total score. | *MANCOVA: age, gender, race, maternal education,  paternal education, age of onset, type of bipolar disorder,  psychotic features, YMRS, HDRS, current usage of lithium,  anticonvulsants or antipsychotics, or antibodies to  other human herpesviruses including HSV- 2, CMV, EBV, varicella zoster  virus, and HHV- 6. *multinomial regression: educational status and BPRS score | **GOOD** |
| **Dickerson et al. (2013)**  *Journal of Affective*  *Disorders*  **sample overlap with Dickerson et al. (2004, 2006, 2014)** | cross-sectional | **107 BD** n=93 (87%) BD-I n=14 (13%) BD-II  n=85 (79%)  inpatients  n=22 (21%) outpatients  **210 HC** | 31 M 76 F | 36.3 (SD 13.4) | 13.1 (SD 2.4) | ? | Yes | serum: hsCRP | -Repeatable Battery for the Assessment of Neuropsychological status (RBANS, Form A):  total score + 5 index scores Immediate Memory, Visuospatial/ Constructional Abilities, Language, Attention, Delayed Memory. -WAIS-III Information Subtest -WAIS-III Letter-Number Sequencing Subtest (LNST) -Trail Making Test A (TMT-A) | *Logistic regression for odds of low RBANS score (<=70 = -2 SD) for High CRP group: >90th% or >75th% of level HC  *Linear regression | -age -gender -race -maternal education -cigarette smoking status -BMI -HAM-D -YMRS -seropositivity HSV-1 | **FAIR** |
| **Dickerson et al. (2014)  sample overlap with Dickerson et al. (2004, 2006, 2013)** | cross-sectional | **347 BD** (also 408 with schizophrenia but separate reporting)  **352 HC** | 101 M (29.1%) 246 F (70.9%) | 34.7 (SD 12.8) | 13.9 (SD 2.5) | 16.2 (SD 12.8) | Yes | Serum: Toxoplasma gondii IgM | Repeatable Battery for the Assessment of Neuropsychological Status (RBANS) with 12 subtests for five index scores + total score ***Immediate Memory** -List Learning -Story Memory ***Visuospatial/Constructional** -Figure Copy -Line Orientation ***Language** -Picture Naming -Semantic Fluency ***Attention** -Digit Span -Coding ***Delayed Memory** -List Recall -Story Recall -Figure Recall -List Recognition | *Linear regression  main analyses with Toxo IgM  *Logistic regression for the odds of low RBANS score (<=82 = <25 percentile of HC) for high Toxo IgM group (>50% or >75% of level HC | Linear regression: age, sex, race, maternal education, and participant  education  logistic regression: also seropositivity HSV-1 IgG and Toxo IgG | **GOOD** |
| **Dittmann et al. (2007)**  *Bipolar Disorders* **Sample overlap with Dittmann et al. 2008** | cross-sectional | **55 BD**  39 BD-I (71%) 13 BD-II (24%) 3 schizo-affective disorder, bipolar type* (5%) (outpatients, euthymic for at least 1 month)  **17 HC** | 29 F (52.7%) | 42.3 (SD 12.8) | 11.7 (SD 1.7) | 16.7 (SD 10.6) | Yes (91%) n=5 (9%) no medication | Plasma: Hcy | -Repeatable Battery for the Assessment of  Neuropsychological Status (RBANS): total score + 5 index scores Immediate Memory, Visuospatial/ Constructional Abilities, Language, Attention, Delayed Memory.  -Trail Making Test (TMT) -WAIS-III subtest Letter–Number Sequencing Test (LNST)  -HAWIE-R (German version WAIS-R) Information Subtest | *Spearman’s correlations  *Linear regression | linear regression:  -age -gender -nr.  of previous episodes -HAM-D -nr. of  psychotropic medications | **FAIR** |
| **Dittmann et al. (2008)** *Journal of Clinical Psychiatry*  **Sample overlap with Dittmann et al. 2007** | cross-sectional | **74 BD** n=51 BD-I (69%) n=23 (31%) BD-II (outpatients, euthymic)  **42 HC** | 50 F  24 M | 42.52 (SD 12.23), range 19-70 | 11.64 (SD 1.71) | 16.7  (SD 10.6), range 1-43 | Yes (93%) n=5 no medication | Plasma: Hcy | ***Information processing speed:** -TMT-A -RBANS Coding ***Working memory** -WAIS-III Letter-Number Sequencing Subtest (LNST) -RBANS Digit Span ***Verbal learning:** -RBANS List Learning -RBANS Story Memroy ***Visuospatial/constructional abilities:** -RBANS Figure Copy -RBANS Line Orientation ***Delayed memory:** -RBANS List Recall -RBANS List Recognition -RBANS Story Recall -RBANS Figure Recall ***Executive functions:** -TMT-B -RBANS Semantic Fluency | *Pearson’s correlations  *Stepwise hierarchical regression | Stepwise hierarchical regression:  -age -gender -nr. of episodes, HAM-D -YMRS -time benig euthymic -nr. of medications -current use of antipsychotics -lifetime psychotic symptoms | **FAIR** |
| **Doganavsargil-Baysal et al. (2013)** *Türk Psikiyatri Dergisi/* *Turkish Journal of Psychiatry* | cross-sectional | **60 BD-I** (euthymic)  **20 HC** (matched for age, gender, and education  level) | ? | ? | ? | 12.13 (SD 8.90) | Yes (98%) n=1 (1.7%) no medication | Serum: -Hcy  -vit B12  -methionine  -folate | -Wisconsin card sorting test (WCST)  -Rey’s auditory verbal learning test (RAVLT)  -Cancellation test (CT)  -Trail making test (TMT) A+B -Stroop test | -Pearson’s correlation  -Spearman’s correlation  -Logistic regression analysis | Bonferroni correction | **POOR** |
| **Garés-Caballer et al. (2022)** *Frontiers in Neurology* | longitudinal 1-year follow-up cohort study | **42 BD at baseline** (out of total sample of n=165  including 30 SZ, 35 MDD, 30 DM type 2, 28 HC)  (BD were clinically stable)  **29 BD at** **1-year FU** (out of total sample of n=125) | 21 F (50%) 21 M (50%) | 50.0 (SD 9.5) | 11.6 (SD 4.4) | 23.4  (SD 11.5) | Yes?  “The participants’ treatment as usual was continued  without no specific interventions” | Serum: -IL-6 -IL-10 -TNF-a -PCR-us  -GSH -ROS -mROS -SOD  *Hemogram: -RBC -HGB -HCT -PLT -WBC -N -AN -L -AL -M -AM | ***cognitive flexibility:**  -Stroop Color and Word Test (SCWT)  -Color/Word Subtest  -Wisconsin Card Sorting Test Categories Completed and Perseverative Errors scored ***verbal fluency:** -Verbal Fluency Tasks Semantic and Phonemic Forms ***working memory:** -Trail Making Test (TMT) Part B -Wechsler Adult Intelligence Scale Third Edition (WAIS-III) Digit Span-B Subtest ***processing speed: -**Finger Tapping Test -WAIS-III Digit Symbol Coding Subtest -SCWT Color and Word Subtests -TMT Part A  Executive domain (ED) was calculated by averaging the four executive functions’ total scores. | linear regression analysis with a predictive models (biomarkers at baseline, executive function at 1-year FU)  first univariate analyses, then multivariate predictive models with a selection of biomarkers (max 5) with most statistical significance | max. five variables were included in each predictive model (so each model included different biomarkers, but no adjustment for confounders) | **POOR** |
| **Gerber et al. (2012)** replication study for Dickerson et al. (2006)  *Progress in Neuro-Psychopharmacology & Biological Psychiatry* | cross-sectional | **30 BD**  (outpatients, two thirds was BD-II, euthymic  <7 HRSD-21, <7 YMRS)  **20 HC** matched for gender, age, education | 60.0% F | 42.6 (SD ?) | 11.8 (SD ?) | 16.2  (SD 11.9) | Yes (90%) | Serum: HSV-1,  HSV-2, CMV, HHV-6, influenza-B, and T. gondii | -Repeatable Battery for the Assessment of Neuropsychological Status, Form A (RBANS) total score + 5 index scores Immediate Memory, Visuospatial/ Constructional Abilities, Language, Attention, Delayed Memory. -Trail Making Test (TMT A&B)  -WAIS-III Letter-Number Sequencing Task (LNST -WAIS-III general information subtest | ANOVA Covariance analyses  Stepwise discriminant function analysis | Unclear. “The association of HSV-1 antibodies with  cognitive impairment proved to be independent of clinical and  demographic determinants” However, separate Pearson correlations between clinical and demographic determinants and cognitive outcomes were performed. | **POOR** |
| **Hebbrecht et al. (2022)** *Neuropsychobiology* | Longitudinal study | **67 BD** 42 BD-I 23 BD-II 2 schizoaffective disorder* (n=35 with depression at baseline (BD-D), n=32 with (hypo)mania at baseline (BD-M)  T0: baseline T1: 4 months  T2: 8 months  **29 HC** (matched for age and sex) | 39 “Gender” (unclear which) | BD-D; 43.7 (SD9.7)  BD-M: 42.4±12.7 | ? | BD-D: 20.6 (10.2)  BD-M:  14.3 (11.8) | Yes (91%)  n=6 (9%) no medication | Plasma: -3-HK  -QA -KYNA -3-HK/KYNA ratio | ISBD-BANC, 5 subtasks: -Y-BACS (processing speed) -CPT-IP (vigilance/ sustained attention) -HVLT-R (verbal learning & memory) -LNS (working memory) -D-KEFS-CWIT (executive function: response inhibition)  Cognition was measured at three test moments. | *Stepwise linear mixed models with outcome global cognitive task score. *Univariate analyses with outcomes separate NP tests  The association  between cognition and KYNs was investigated by combining the data of the three test moments. | -subject ID as random effect -group (BD-D, BD-M, HC) -moment -significant group ×  moment interactions  -sex -age -smoking -BMI -years of education | **POOR** |
| **Hidese et al. (2023)**  *Neuropsychopharmacology Reports* | cross-sectional | **115 BD** 38 BD-I, 77 BD-II  (also 149 schizophrenia & 186 unremitted MDD but separate reporting)  **350 HC (**matched for sex and Japanese ethnicity) | 54 M, 61 F | 40.8 (SD 11.3) | 15.1 (SD 2.6) | 11.2 (SD 7.8) | Yes, (84.3%, n=97)  n=80/69.6% use of mood stabilizers | Plasma: -α-MSH -β-endorphin -neurotensin -oxytocin -substance P  (these 5 neuropeptides were also measured in CSF but this was not reliable) | ***General cognitive function:** Japanese version of the Brief Assessment of Cognition in Schizophrenia (BACS):  BACS verbal memory  BACS working memory  BACS motor speed  BACS verbal fluency  BACS attention  BACS executive function ***Memory function** Japanese version of the Wechsler Memory Scale-Revised (WMS-R): WMS-R verbal memory  WMS-R visual memory  WMS-R general memory  WMS-R attention/ concentration  WMS-R delayed recall | Pearson's partial correlation coefficient | age,  sex, BMI, education level, current smoking status, and psychotropic  medication use  Bonferroni corrections for multiple testing of the five plasma neuropeptide levels  (p < 0.05/5 = 0.01). | **FAIR** |
| **Huang et al. (2021)** *Journal of Psychiatric Research* **sample overlap with Huang 2022?** | cross-sectional | **77 BD-I**  (outpatients)  Subgroups: n=21 BD with suicidal ideation (SI) and n=56 without SI (SI= ≥1 on item 10  of MADRS)  **61 HC** (matched for age and sex) | 47 F (61%) | BD without SI: 36.9  (SD 11.3) BD with SI:  33.4  (SD 15.1) | BD without SI: 13.3 (SD 3.1)  BD with SI: 13.2  (SD 2.6) | ? | Yes (96%) n=3/3.9% no medication | serum: -sIL-6R -CRP -sTNF-αR1 | -2-back task (working memory): correct, error, omission -Go/No-Go task: correct, error, omission | Linear regression,  main analyses are SI on biomarkers as outcomes, adjusted for go-no go task or 2-back task | age, sex, education, history of suicide attempts, total MADRS  score, total YMRS score + adjusted for go-no go task or 2-back task | **POOR** |
| **Huang et al. (2022)** *Psychiatry and Clinical Neurosciences* **sample overlap with Huang 2021?** | cross-sectional | **70 BD** 37 BD-I,  33 BD-II  (also 25 **MDD**) **54 HC** (age-, sex-matched) | BD-I: 19 F (51.4%)  BD-II: 20 F (60.6%) | BD-I 34.9 (8.2)  BD-II 31.5 (8.5) | BD-I 14.1 (2.8)  BD-II 14.9 (2.0) | not collected | Yes | serum: -sIL-6R -CRP -sTNF-αR1 | ***Learning and memory:** -Word List Memory Task (WLMT) ***Executive function:** -Wisconsin Card Sorting Task (WCST) ***Working memory** Test of Attentional Performance (TAP) 2-back task ***Sustained attention and inhibitory control:** -TAP Go/No-Go task ***Divided attention (visual/auditory):** -TAP subtest for divided attention | Linear regression models (GLM) using the complete group (BD, MDD, HC), but including interaction for diagnostic groups | age, sex, education,  BMI, YMRS total scores, MADRS total scores, psychotropic medication  use, and diagnostic group | **FAIR** |
| **Hui et al. (2019)** *International Journal of Bipolar Disorders* | cross-sectional | **37 BD** no info on BD subtype  **37 HC** (matched for gender and age) | 15 M, 22 F | 29.78 (SD 10.05) | 10.14 (SD 2.98) | 8.96 (SD 8.5) | 89.2% (n=4 10.8 % no mood stabilizer) | serum: HDL | RBANS (Form A): total score + 5 index scores: immediate memory, visuospatial/ constructional domain, language, attention, delayed memory. | *Pearson’s product moment correlation  *Stepwise multiple regression | Stepwise multiple regression:  -age -gender -education -BMI  -age of onset -duration of illness -nr. of episodes -nr. of hospitalizations -medication dosage -clinical sypmtoms (HAMD, BRMS) | **FAIR** |
| **Jakobsson et al. (2013)** *Neuropsychopharmacology* | cross-sectional | **139 BD** 64 BD-I (46.0%), 53 BD-II (38.1%),  1 cyclothymia (0.7%), 2 schizoaffective disorder manic type* (1.4%),  19 BD-NOS (13.7%)  **71 HC** | 55 M (39.6%) | median 36, IQR 28-50 | ? | ? | Yes | CSF: -T-Tau -P-Tau -Aβ1-42 -Aβ38 -Aβ40 -Aβ42  -sAPP-α  -sAPP-β -Aβ42/40 ratio -Aβ42/38 ratio | WAIS-III: 4 index scores: comprehension, perceptual organization, working memory, processing speed.  -TMT: visual scanning (TMT-1), number sequencing (TMT-2), letter sequencing (TMT-3), number-letter switching (TMT-4), and motor speed (TMT-5). | *Spearman’s correlations | correction for multiple testing (False Discovery Rate) | **POOR** |
| **Jonsson et al. (2022)**  *Brain and Behavior* | cross-sectional | **121 BD** (69 BD-1, 40 BD-II, 12 BD-NOS)  (all euthymic) **30 HC** (matched) | 49 M, 72 F | 46.38 (SD 1.25) | ? | ? | Yes, lithium 58%, antipsychotics 28%, antidepressants 38%, sedatives 32%, anti-epileptics 26% | serum: zinc | Delis–Kaplan Executive Function System (D-KEFS):  - the Color-Word Interference Test (CWIT), measuring the ability to inhibit automatic verbal responses - The Verbal Fluency Test (VFT), providing information about language skills and verbal processing ability, as well as problem-solving and inhibition,  - The Trail Making Test (TMT),  measuring cognitive flexibility, visual attention, and motor speed. | PCA analysis to create a single executive functioning score based on 52 cognitive subtests to create PC1 and PC2  correlation  analysis with PC1 and PC2 as dependent variables and serum zinc levels as independent variable  partial correlation  analyses | partial correlation: adjusted for IQ | **FAIR** |
| **King et al. (2019)** *Frontiers in Psychiatry* | cross-sectional | **15** **BD-II (of which 13 with available blood samples)** (12 euthymic, 3 depressed)  **13 HC**  **(of which 10 with available blood samples)** | ? | ? | ? | ? | Yes (33%) (n=10/67% were medication-free) | “Blood samples”: -Glu -CRP -IL-6 -TNFα | ***executive functioning:** -IntraDimensional ExtraDimensional Shift of the Cambridge  Neuropsychological Test Automated Battery -TMT-B (executive function) ***psychomotor processing speed:** -WAIS Digit Symbol Substitution Test (DST) (processing  speed) ***verbal learning & memory:** -Rey Auditory Verbal Learning Test (RAVLT) (verbal learning & memory) | Pearson product- moment correlations | correction for multiple comparisons (False Discovery Rate) | **POOR** |
| **Knöchel et al. (2017)** *European Archives of Psychiatry and Clinical Neuroscience* | cross-sectional | **25 BD-I** (euthymic)  (also 29 schizophrenia, but separate reporting) **93 HC** (matched for age, gender, years of education) | 6 F 19 M | 37.79 (SD 10.40) | 15.19 (SD 2.37) | 8.860 (SD 5.47) | Yes (100%) | 42 plasma proteins (among others: proteins for lipid transport, complement system, acute-phase proteins, immunological) see Table 2 | ***psychomotor speed** -Trail Making Test A (TMT-A) ***executive functioning** -TMT-B | Bivariate correlation analyses using Pearson  product–moment or Spearman rank correlation coefficients (only for those proteins with significant group comparisons of BD+SZ vs. HC) | Bonferroni correction | **POOR** |
| **Lee et al. (2017)** *Progress in Neuro-Psychopharmacology & Biological Psychiatry*  **Same sample as Lee et al. (2018)** | cross-sectional | **32 BD-II**  (from acute ward and outpatient clinics, newly diagnosed, never medication, hypomania was defined as 2-day minimum of having symptoms)  **30 HC** | 11 M 21 F | 37.9 (SD 10.8) | 13.9 (SD 2.2) | newly diagnosed patients  (age of onset: mean 13.8 SD 4) | No  At baseline, none had a previous history of mood stabilizers or antipsychotics. | Serum: -DHEA -DHEA-S -pregnenolone | Brief Assessment of Cognition in Affective  Disorders (BACA): -verbal memory (list learning) -working memory (digit sequencing) -processing speed (verbal fluency; token motor task; symbol coding) -reasoning and problem solving (tower of London [TOL]) -affective interference (emotional distractibility and affective memory) -emotional disinhibition | MANCOVA + post-hoc for BD-II group and short duration hypomania group | age, gender, years of education  (diagnosis of BP-II) | **FAIR** |
| **Lee et al. (2018)** *Neuropsychiatric Disease and Treatment* **Same sample as Lee et al. (2017)** | RCT (open label valproate) | **32 BD-II**  (from acute ward and outpatient clinics, newly diagnosed, never medication, hypomania was defined as 2-day minimum of having symptoms)  **30 HC** | 11 M 21 F | 37.9 (SD 10.8) | 13.9 (SD 2.2) | newly diagnosed patients  (age of onset: mean 13.8 SD 4) | No (at baseline)  At baseline, none had a previous history of mood stabilizers or antipsychotics.  During follow-up, all 32 patients (100%) received open-label valproate 1500mg, 28/32 used fluoxetine  10/32 used risperdal. | Serum:  cortisol | Brief Assessment of Cognition in Affective Disorders (BACA) seven subtests, including Verbal Memory (List Learning), Working Memory (Digit Sequencing), Processing Speed (Verbal Fluency; Token Motor Task; Symbol Coding), Reasoning and Problem Solving (Tower of London [TOL]), and tests of affective interference (emotional distractibility and affective memory) and emotional disinhibition, which are then summed up as affective composite scores. | Pearson correlation for cortisol and cognition at baseline | - | **POOR** |
| **Li et al. (2015)**  *Psychoneuroendocrinology* | cross-sectional | **27 BD-I** (non-diabetic, stable)  (also n=27 unaffected siblings)  **30 HC** (matched for age, gender, ethnicity) | 15 M 12 F | 39.7 (SD 9.8) | n=13 ≤12 years n=11 12—16 years n=3  > 16 years of education | 13.4 (SD 9.3) | No (clinically stable off medications or stopped medications for at least five days before the enrollment) | Plasma  -FPG -fasting insulin -HOMA-IR =  FPG × fasting insulin/405 | ***Executive functions:** -Wisconsin card sorting test (WCST): % errors, % conceptual level responses, Categories completed | Pearson’s correlation | No | **POOR** |
| **Liou et al. (2023)** *Psychoneuroendocrinology* | cross-sectional | **641 BD and 150 BD + AUD (alcohol use disorder)**  (outpatients, majority in affective episode (BD group: 9.3% euthymic, BD+AUD group: 12.5% euthymic)  **185 HC** | 315 M, 326 F | BD: 32.9 (SD 12.4)  BD+AUD: 38.3 (SD 10.5) | BD: 12.1 (SD 3.2)  BD+AUD: 13.8 (SD 3.2) | ? | Yes (100%) All BD patients were treated with valproate. | Plasma:  -TNF-α -CRP -IL-8 -TGF-β1 -BDNF | -Wisconsin card sorting test (WCST) -continuous performance test (CPT) -Wechsler memory scale - third edition (WMS-III) | Pearson correlation  Linear regression | regression: age, sex, education, HDRS, YMRS | **FAIR** |
| **Lotrich et al. 2014** *International Journal of Geriatric Psychiatry* | cross-sectional | **21 BD**  (inpatients and outpatients, age ≥50 years, euthymia for at least 4 weeks, HDRS<10, YMRS<10) **26 HC** (matched for age, education, gender, and  cardiovascular burden) | 61.9% F | 64.8 (SD 9.1) | 15.0 (SD 2.4) | ? | ? | Serum:  -IL-1RA | Four factor analysis-derived z-scores and a global z-score, based on 21 neuropsychological tests. 1. language;  2. delayed memory 3. visuomotor ability 4. information processing speed/executive function.  A global z-score was determined on the basis of all 21 individual tests. | Spearman  correlations   Variables that were correlated  with IL-1RA (p ≤ 0.10) were considered for  multiple linear regressions | In separate analyses (HC+BD): covarying for BDNF, BMI, IL-6, BD diagnosis | **POOR** |
| **Mansur et al. (2020)** *Psychoneuroendocrinology* | RCT (infliximab) | **60 BD**   (outpatients, MADRS <22, YMRS <12) | 46 F 14 M | 45.6 (SD 10.9) | high school 11  college 41  graduate 6 | ? | yes | Plasma:  leptin  TNF-α  sTNF-αR1  sTNF-αR2 | -Digit Symbol Substitution Test (DSST)  -Rey Auditory verbal learning test (RAVLT) (immediate recall, delayed recall) | generalized estimating equations (GEE) at baseline | age, gender, BMI, use tobacco and MADRS score | **POOR** |
| **Millett et al. (2020)** *Brain, Behavior, and Immunity*  **sample overlap with Millett et al. 2021 and Peters et al. 2022** | cross-sectional | **219 BD** 176 BD-I  (euthymic outpatients)  **52 HC** | 103 F 116 M | 43.6 (SD 11.9) | 14.2 (SD 2.5) | 21.9 (SD 11) | yes (77.6%) | Serum:  TNF-α  sTNF-αR1  sTNF-αR2 | ***executive function:**  -Controlled Oral Word Association Test (COWAT)  -Stroop  -Wisconsin Card sorting Test (WCST)  MATRICS consensus cognitive battery (MCCB) | -pearson correlation  (Structural equation model) | age and sex | **FAIR** |
| **Millett et al. (2021)**  *Molecular Psychiatry* **sample overlap with Millett et al. 2020 and Peters et al. 2022** | cross-sectional | **222 BD** 179 BD-I  (80.3%)  (euthymic outpatients)  **52 HC** | 105 F (47.3%) 117 M | 44  (SD 11.9) | 14.4 (SD 3.4) | 22 (SD 11.5) | Yes (78%)  49 (22%) not on medication nr of medications: 1.7 (SD 1.3) | Serum:  CRP | six out of seven of the MATRICS consensus cognitive battery (MCCB) domains: Speed of Processing, Attention and Vigilance, Working Memory, Verbal Learning, Visual Learning, Reasoning and Problem-solving,  and Social Cognition. The MCCB verbal learning test (the Hopkins Verbal Learning Test) was replaced with the more difficult California Verbal Learning Test (CVLT). → MATRICS composite score  ***executive function:** -Stroop color and word test  -Controlled Oral Word Association Task  (COWAT) -Wisconsin Card Sorting Task (WCST) ***theory of mind:**  Reading Mind in the Eyes task | *MANCOVA to compare groups with high (≥5 mg/L) and normal CRP (<5 mg/L) on cognitive tests; in total group and BD only group  *Pearson correlations  and then r-to-z transformation analyses | omnibus testing approach to control against false positives (Type-I  error rate)  Partial correlations: controlled for age, sex and education. | **FAIR** |
| **Miskowiak et al. (2023)** *Journal of Psychiatric Research* | longitudinal study | **60 BD** (newly diagnosed patients, in remission)  T0 baseline,  T1 during affective episode T2 remission of affective episode T3 after 1 yr **HC** age and gender matched | 28 F, 32 M | median 32 (Q1-Q3 1-4) | median 14 (Q1-Q3 12-16) | newly diagnosed patients | Yes, 48.3% lithium, 41.7% antipsychotics, 48.3% anticonvulsants, 8.3% benzodiazepines, 1.7% antidepressants | CSF: -EPO -8-oxo-Guo -8-oxo-dG  Urine: -8-oxo-Guo -8-oxo-dG | -SCIP (Screen of Cognitive Impairment in  Psychiatry) -Rey Auditory Verbal  Learning Test (RAVLT) -Repeatable Battery for the Assessment of  Neuropsychological Status (RBANS) -Digit Span -Wechsler’s Adult Intelligence Scale, 3rd edition  (WAIS-III) digit-letter substitution test - verbal fluency tests with letters S and D  - Trail Making Test (TMT) part A and B - Rapid Visual Processing Test (RVIP) from the Cambridge Neuropsychological Test Automated Battery (CANTAB)  Cognition was measured at T0, T2 and T3. | exploratory analysis: linear mixed model with HC and BD separately | exploratory analysis: adjusted for multiple testing with method of Benjamini  & Hochberg. fixed effects 1) age, sex 2) 1+ years of education, smoking, alcohol | **FAIR** |
| **Mora et al. (2019)** *European Psychiatry* | cross-sectional | **84 BD**  (euthymic outpatients)  **49 HC** | 40F  44 M | 44.4 (12.4) | 14.4 (3.4) | 17.4 (11.8) | yes (98%) | Serum:  -BDNF  **-**IL-6 -TNFα -IL-10 -TBARS | ***executive functions:**  -TMT B  -FAS  -Digit span backward  -number categories WCST  ***inhibition**  -Stroop inhibition  -persverative errors WCST  -perseverative errors CPT  ***attention**  -Stroop interference  -Digit span forward  -CPT detectability  ***processing speed**  -TMT A  -CPT hit reaction time  ***verbal memory**  -CVLT: first trial, total, immediate recall, delayed recall, recognition  ***visual memory**  -RCFT immediate recall  -RCFT delayed recall | MANOVA, multiple regression | age, premorbid IQ, BMI, neurobiological variables | **FAIR** |
| **Na et al. (2020)**  *Iran Red Crescent Medical Journal* | cross-sectional | **100 BD-I**  (in-and outpatients)  **70 HC** | 50 F 50 M | 31.2 (SD 10.7) | 10.2  (SD 3.6) | 2.3 | ? | Serum:  -Hcy  -folic acid | -TMT A/B  -Stroop | Spearman correlation | no | **POOR** |
| **Omileke et al. (2019)** *Neuropsychopharmacology Reports* | cross-sectional | **26 BD**  recruitment via local advertisement  **51 HC** | 14 F 12 M | 44.5 (SD 14.52) | 14.0 (range 12.0-16.0) | 10.0 (range 4.8-15.5) | yes | Plasma:  -FGF21 | BACS: -verbal memory -working memory -motor speed -category fluency -letter fluency -attention -executive function → total composite score | Spearman correlation | no | **POOR** |
| **Osher et al. (2008)** *Journal of Affective Disorders* | cross-sectional | **57 BD**  (euthymic outpatients)  **84 HC** | 22 F  35 M | 39.4 (SD 12.7) | ? | 13.5 (SD 10.3) | yes (98%) | Serum:  -Hcy  -folate level  -vit B12 | Benton visual retention test  Digit symbol  verbal fluency  RVALT  Digit span  Stroop  WCST | ANCOVA | age | **POOR** |
| **Paribello et al. (2023)** *Brain Sciences* | cross-sectional | **50 BD of which  45 BD with cognitive data for analysis** 32 BD-I (71%) and 13 BD-II (29%)  (euthymic HDRS <14 and YMRS <13)  n=16 lifetime suicide attempts (LSA);  n=22 lifetime history of suicide ideation (LSI) **48 HC** | 30 F,  15 M | non-LSA n=33 mean 51 median 53 SD 8.5  LSA n=17 mean 50 median 49 SD 8.8 | females: <11 yrs (n=7, 23%), 11-13 yrs (n=14, 47%), 13 yrs (n=9, 30%).  males: <11 yrs n=6 (40%), 11-13 yrs (n=7, 47%), +13 yrs (n=2, 13%) | 28.4 years | Yes (96%)  43 out of 45 subjects were prescribed a pharmacological  therapy | Plasma:  -5-HTP -TRP -5-HT  -KYN -QA -KYNA -3-HK -5-HTP/ TRP ratio -KYN/TRP*1000 ratio -QA/KYNA ratio | Brief Assessment of Cognition in Affective Disorder (BAC-A):  -categorical and letter fluency, -Tower of London -digit sequencing task, verbal memory -token motor task  -affective interference tests (further divided into the affective  processing and delayed recognition subtasks) -emotion inhibition subtask (EIS).  The EIS has 4 subtasks: -color naming (CN) -neutral color words -affective color words -neutral words (NW) | linear regression | gender as  a cofactor and total time of illness at recruitment (months) as covariate. | **FAIR** |
| **Permoda-Osip et al. (2014)** *Psychiatria Polska* | cross-sectional | **116 BD** 37 BD-I 79 BD-II   (hospitalized during acute episode) | 83 F 23 M | 51 (SD 13) | ? | 8 (SD 5) | yes | Serum:  -Hcy  -folate  -vit B12 | TMT  WCST  Stroop  WAIS-R | Pearson correlation | no (M/F) | **POOR** |
| **Peters et al. (2022)** *Journal of Affective Disorders*  **Sample is a subset of the sample of Millett et al. (2021)** | cross-sectional | **119 BD** 92 BD-I  (euthymic outpatients) | 49 F (41.2%) | 47.49 (SD 9.9) | 14.04 (SD 2.42) | 24.1  (SD 10.62) | yes,  on average  1.61 SD 1.32 medications | Serum:  CRP | affective Go/no-go task from the CANTAB: 9 subitems: -negative/positive/neutral d’prime = target detection -negative/positive/neutral hits response time (RT) -negative/positive/neutral commissions response time (RT) | univariate: bivariate correlations  multivariate: stepwise regression with log transformed CRP | multivariate: 1st step: HDRS, YMRS 2nd step: age, sex, race, education, smoking, lifetime psychosis, no. psychotropic medications, BD illness duration, no. psychiatric hospitalizations, no. mood episodes | **FAIR** |
| **Platzer et al. (2017)** *Psychoneuroendocrinology* | cross-sectional | **68 BD- I and BD-II**  (euthymic)  **93 HC** | 26 F, 42 M | 45.3 (SD 13.9) | 44.2% completed high school | ? | ? | Serum:  TRP  KYN  KYNA 3-HK  3-HK/ KYNA ratio  KYN/ 3-HK ratio  KYN/ KYNA ratio | -multiple choice word test  ***verbal learning/memory**  -CVLT  ***attention/speed**  -TMT A  -D2  ***Executive functioning**  -TMT B  -Stroop interference | partial correlations | no (M/F) | **POOR** |
| **Poletti et al. (2021)** *Journal of Psychiatric Research* | cross-sectional | **76 BD**  (inpatients with depressive episode) | 53 F, 23 M | 47.1 (SD 11.3) | 12.8 (SD 3.8) | 19.2 (SD 11.5) | ? | Plasma:  IL-1β, IL-1RA, IL-2, IL-4, IL-5, IL-6, IL-7,  IL-8, IL-9, IL-10, IL-12, IL-13, IL-15, IL-17A, CXCL10,MCP-1, MIP-1α  MIP-1β RANTES, Eotaxin, TNF-α, IFN-γ,  bFGF,  PDGF-BB, G-CSF, GM-CSF, VEGF | BACS: verbal memory, working memory, psychomotor speed, selective attention, semantic fluency, letter fluency and executive function.  for each cognitive function:  subgroups BACS equivalent scores poor (score <=1) and good (score>1) performers. | elastic net penalized logistic regressions.  The accuracy of the model was estimated by a 10-folds stratifie  nested cross-validation.  second step: Generalized Linear Model  (GLZM) with Logit link function. | Elastic net models: age, sec, education frequency mood episodes, BMI, imipramine and chlorpromazine doses, lithium, HDRS.  GLZM: Only significant covariates from Elastic net models included. | **POOR** |
| **Reininghaus et al. (2016)**  *Bipolar Disorders* | cross-sectional | **112 BD**  (euthymic)  **80 HC** | 45.5% F | 45.4 (SD 15.0) | ? | 20.1 (SD 13.6) | ? | Serum:  -MMP9  -sICAM-1 | multiple choice words test  ***verbal learning**  CVLT  ***attention/psychomotor**  TMT A  Stroop  D2  ***executive functioning**  Stroop interference  TMT B | partial correlations | education, age, smoking and BMI | **FAIR** |
| **Rolstad et al. (2015a)** *PloS ONE* | cross-sectional | **82 BD** 53 BD-I, 29 BD-II  (outpatients)  **71 HC** | 48 F 34 M | 38.3 (SD 12.5) | 12.9 (SD 2.8) | ? | yes | CSF: -sAPP-α -sAPP-β -T-tau  -P-tau  -Aβ1-42 -Aβ42/40  -Aβ42/38 -NFl | ***speed/attention**  -TMT number sequencing D-KEFS  -Digit symbol WAIS-III  ***learning/memory**  -CleasonDahl verbal memory  -Rey complex figure recall  -digit span WAIS-III  -letter number sequencing WAIS-III  ***visuospatial**  -Rey copy  -Block design WAIS-III  ***verbal functions**  -Similarities WAIS-III  -verbal fluency D-KEFS  ***executive function**  -Stroop D-KEFS  -design fluency D-KEFS  -TMT number-letter D-KEFS  -Tower test D-KEFS  -CPT | linear regression | age, bipolar type, CGI, MADRS, YMRS, GAF, use mood stabilizers, antidepressants, antipsychotics, benzodiazepines, anxiolytics | **POOR** |
| **Rolstad et al. (2015b)** *European Neuropsychopharmacology* | cross-sectional analysis of longitudinal cohort | **78 BD** 48 BD-I (61.5%) and 30 BD-II   (euthymic outpatients, MADRS<14, YMRS <14)  **86 HC** | 47 F 31 M | 38.2 (SD 13.4) | 44.3% university | ? | yes | CSF:  YKL-40  TIMP1 TIMP2  MCP-1  sCD14 | ***speed/attention**  -TMT number sequencing D-KEFS  -Digit symbol WAIS-III  ***learning/memory**  -CleasonDahl verbal memory  -Rey recall  -digit symbol WAIS-III  -letter number sequencing WAIS-III  ***visuospatial**  -Rey copy  -Block design WAIS-III  ***verbal functions**  -Similarities WAIS-III  -verbal fluency D-KEFS  ***executive function**  -Stroop D-KEFS  -design fluency D-KEFS  -TMT number-letter D-KEFS  -Tower test D-KEFS  -CPT | hierarchical linear regression | age, bipolar type, CGI total, MADRS, YMRS, GAF, use mood stabilizers, antidepressants, antipsychotics, benzodiazepines | **POOR** |
| **Rubin et al. (2014)** *Schizophrenia Bulletin* | cross-sectional | **75 BD** (psychotic bipolar disorder)  (also n=61 relatives, n=57 schizophrenia)  **66 HC** | 43 F 32 M | 32.9 (SD 13.6) | 12.9 (SD 2.5) | ? | yes | Serum:  OT  AVP | -The Penn Emotion Recognition-40 test (PENN ER-40)  -BACS → composite score | linear regression | age, race and sex | **FAIR** |
| **Sağlam Aykut et al. (2018)** *Nordic Journal of Psychiatry* | cross-sectional | **28 BD**  (outpatients euthymic)  **22 HC** | 18 F 10 M | 38.9 (SD 12.2) | 10.9 (SD 3.9) | 12.7 (SD 20.1) | Yes | Serum:  NLR  PLR | WCST  Stroop  R-AVLT | Pearson and Spearman correlations | no | **FAIR** |
| **Salvi et al. (2020)**  *Journal of Affective Disorders* | cross-sectional | **100 BD** (59 BD-I)  n=24 with insulin resistance (IR) and n=71 without IR | F (51%) | 45.2 (SD 13.3) | IR group: 12.9 (SD 3.3 non-IR group: 14.9 (SD 2.7) | IR group: 19.9 (SD 14.4) non-IR group: 19.3 (SD 13.4) | Yes | Plasma: -basal insulin -glucose  -total cholesterol -HDL  -triglycerides (TGC) | -California Verbal Learning Test (CVLT) subtests: trials 1–5, trial 6, and trial 8 (total recall, short-delayed recall, long-delayed recall).  -Digit Span: forward and backward  - composite verbal memory score =  (trials 1–5 z-score + trial 6 z-score + trial 8 z-score) / 3  - composite working memory score =  (DS forward z-score + DS backward z-score) / 2 | general linear model  if significant in a bivariate analysis, variable was entered in a multivariable model | age as covariate and site as random factor | **FAIR** |
| **Sanchez-Autet et al. (2018)** *Journal of Affective Disorders* | cross-sectional analysis of longitudinal cohort | **224 BD-I, BD-II and BD-NOS**  (outpatients, all moods allowed) | 146 F 78 M | 47.1 (12.5) | 12.9 (4.7) | 19.5 (11.9) | yes | Serum:  -CRP  -Hcy | SCIP:  working memory, verbal learning immediate, verbal fluency, verbal; learning delayed, speed processing | partial correlations  hierarchical multiple regression | age, years education, number hospital admissions, HDRS, daily tobacco consumption, MBI | **GOOD** |
| **Strawbridge et al. (2021)** *The British Journal of Psychiatry Open (BJPsych Open)* | cross-sectional | **44 BD** 26 BD-I, 18 BD-II  (euthymic for at least 1 month, <=7 HSRD and YMRS) | 13 M,  31 F | 43.7 (12.8) | measured but not stated | - | yes,  on average 3.7 SD 2.2 medications | Plasma: BDNF, bFGF, Flt-1, PIGF, Tie-2, VEGF-C, VEGF-D, VEGF  CRP, Eotaxin, Eotaxin-3, ICAM-1, IFN-g, IL-10/12/15/16/17/1a,/6/7/8, IP-10,MCP-1, MCP-4, Mip-1a, Mip-1b, SAA, TARC, TNF-a, TNF-b, VCAM-1 | → global cognitive performance as a continuous measure calculated from 8 tests across 4 domains: ***processing speed:** -Digit symbol Substitution Test -symbol search (WAIS) ***working memory:** digit span (WAIS) ***verbal learning and memory:** -verbal paired associates tests I and II (Wechsler Memory Scale) ***executive functioning:** -hotel test -matrix reasoning (WASI) -F-A-S test → cognitive impairment as dichotomous outcome: if >= 1 SD below published norms on two or more cognitive tests | univariate associations: Spearman’s correlation for global cognitive performance and t-tests for cognitive impairment  → p<0.1 a priori selected for multivariable models.  multivariable:  linear regression for outcome global cognitive performance  logistic regression for outcome cognitive impairment | non-biological confounders were selected from the spearman’s correlation and t-tests if p<0.01. These differed per model, but included: number of medications, smoking, bipolar type, physical illness, FAST, gender, age, number of episodes, CTQ, HRQOL | **FAIR** |
| **Tanaka et al. (2017)** *Neuroscience Research* | cross-sectional | **32 BD-I and BD-II**  (outpatients)  (also: 82 SZ + schizoaffective, but separate reporting)  **32 HC** | 18 F 14 M | 38.7 (7.5) | 13.6 (2.4) | ? | yes | Serum:  -CMV IgG -CMV IgM  -T gondii IgG -T gondii IgM  -HSV-1 IgG -HSV-2 IgG  -CRP  -pentraxin  -sCD14 | BACS: working memory, motor speed, verbal fluency, attention, speed of information processing and executive function | regression analysis | age and smoking status | **POOR** |
| **Thompson et al. (2005)** *British Journal of Psychiatry* | cross-sectional | **63 BD** 54 BD-I 9 BD-II 5 rapid cycling  (outpatients euthymic)  **63 HC** | 26 F 37 M | 44.4 (8.6) | 14.2 (3.) | 19.5 (10.0) | yes | Saliva:  cortisol | ***Psychomotor:**  -Vigil test  -Digit symbol substitution  -TMT A  ***Attention/executive functions:**  -TMT /B  -Stroop  -TOL  -COWAT  -Digit backwards  -abstract design SOPT  -spatial working memory CANTAB  ***Immediate memory:**  -Digits forward  -CANTAB spatial span  Declarative memory:  -CANTAB pattern ***recognition memory and spatial memory**  -CANTAB simultaneous and delayed matching  ***visual memory**  -CANTAB paired associates learning  -R-AVLT | Spearman | no | **POOR** |
| **Tournikioti et al. (2021)** *Neuropsychobiology* **same sample as Tournikioti et al. (2022)** | cross-sectional | **60 BD-I**  (50% euthymic outpatients and 50% partially remitted inpatients)  **30 HC** | 37 F 23 M | 44.5 (36-53.7) | 12 (9.25-16) | 16 (7.25-21) | yes (100%) | Serum:  -cortisol  -DHEA-S  -cortisol/DHEA-S ratio | CANTAB ***visuospatial memory**  -spatial recognition memory (SRM)  ***executive functions**  -stockings of Cambridge (SOC)  -intradimensional/extradimensional attentional set shifting, total errors adjusted (ID/ED) | linear regression | gender, age and education | **FAIR** |
| **Tournikioti et al. (2022)** *Psychiatry Research* **same sample as Tournikioti et al. (2021)** | cross-sectional | **60 BD-I**  (50% euthymic outpatients and 50% partially remitted inpatients) | 37 F 23 M | 44.6 (11.5) | 12 (9.25-16) | 16 (7.25-21) | yes | Serum:  cortisol | CANTAB ***visuospatial learning**  -paired associative learning (PAL)  ***executive functions**  -stockings of Cambridge (SOC) | stepwise multiple regression analysis | gender education  illness duration  current use of mood stabilizers | **POOR** |
| **Tozoglu et al. (2021)** *Archives of Clinical Psychiatry* | cross-sectional | **40 BD**  (outpatients, in remission)  **30 HC** | 21 F 21 M | 36.1 (SD 9.6) | Primary school 40,5%  high school 21,4%  33.3% university | ? | ? | Serum:  NO  ADMA  SDMA  TAC | -RAVLT  -COWAT  -Digit span  -TMT A/B  -Stroop  -WCST | pearson correlations | no | **POOR** |
| **Van der Werf-Eldering et al. (2012)** *PLoS ONE* | longitudinal (over the course of one day) | **65 BD** (53 BD-I,  12 BD-II)   (depressive symptoms were allowed, IDS-SR≤38, YMRS <7)  multiple timepoints: T1: at awakening T2: 30 min after awakening T3: 45 min after awakening T4: 60 min after awakening T5: at 10 PM T6: at 11 PM T7: at awakening | 44 F 21 M | 46.9 (SD 10.4) | (range 1-6) 3.6 (SD 1.0) | 21.8 (SD 13.1) | Yes (96.9%) | Saliva cortisol, during one day with regular activities  -CAR = cortisol awakening response (T1,2,3,4) -mean evening cortisol  (T5/T6) -cortisol diurnal slope decline per hour (T6-T1/hours in between) | **Psychomotor speed:** simple movement time five-choice movement time **Speed of information processing:** Simple reaction time five-choice reaction time Stroop test (2 subscores) **Attentional switching** Continuous Performance Task **(**CPT) (3 subscores) **Verbal memory** California Verbal Learning Test **(**CVLT) (2 subscores) **Visual memory** Pattern Recognition Memory (PRM) (2 subscores) **Executive functioning/ working memory** Spatial Working Memory (SWM) (2 subscores) Stroop interference  ⇒ mean z-score of all 6 cognitive domains | Linear regression | gender, education, IQ  (cognitive data was already age-adjusted) | **FAIR** |
| **Van Rheenen et al. (2021)** *Journal of Affective Disorders* | cross-sectional | **23 BD**  (outpatients, n=10 stable)    **34 HC** | 15 F 8 M | 31.9 (SD 11.9) | ? | ? | yes | Serum:  triglyvceries | ***executive function:**  -Stroop  -TMT-B | -partial correlation  -regression | age | **FAIR** |
| **Van Rheenen et al. (2023)** *Psychiatry Research* | cross-sectional | **55 BD** 52 BD-I 3 BD-II  (no current mood episode or in 3 weeks before) **22 HC** | 23 F, 32 M | vit D deficient: 37.6 (SD 10.3)  vit D sufficient: 37.8 (SD 8) | not stated, only WTAR:  vit D deficient: 106.5 SD 10.3  vit D sufficient: 108.5 SD 11.4 | vit D deficient: 15.12 SD 7.88  vit D sufficient: 16.07 SD 7.69 | yes (100%)  All BD patients had been on a stable medication  regime for a period of at least 2 months Medication load: 1.7/1.8 SD 1.3 | plasma: vit D | MATRICS Consensus Cognitive Battery (MCCB): The MCCB assesses six cognitive domains: processing speed, attention, working memory, visual learning, executive function,  and social cognition.  → A composite score reflecting global cognition | univariate ANCOVAs in full sample BD+HC with vit D status x group interaction term and in BD sample only  bivariate correlation | FDR of p<0.05 was applied using the Benjamini-Hochberg method  bivariate correlations: conservative alpha of p=<0.01  model of global cognition: corrected for season of blood draw and WTAR | **POOR** |
| **Zazula et al. (2022)** *The World Journal of Biological Psychiatry* | cross-sectional | **31 BD** 17 BD-I (54.8%) 14 BD-II  (outpatients, regular visit)  **27 HC** | 25 F 6 M | 39.5 (SD 11.5) | Primary school 7.4%,  secondary school 14.8%,  university 54.8% | ?  age of onset: 20.5 (SD 7.2) | yes | Plasma:  TNF-α  sTNF-αR1  sTNF-αR2 | CogState research battery: ***executive functioning:**  -Groton maze learning test (GML)  -set shifting task (SETS)  ***working memory**  -Two back task (TWOB) | -Spearman  -multiple regression | -age  -gender  -anxious, depressive and manic symptoms | **FAIR** |

*Our review excluded studies with a mixed sample (BD combined with other diagnoses). However, this study was included as an exception, because the authors considered ‘schizoaffective disorder, bipolar type’ a BD subtype.
