# Supplementary material for: Biofluid Biomarkers of Cognitive Functioning in Bipolar Disorder: A Systematic Review by the Targeting Cognition and Older‐Age Bipolar Disorder ISBD Task Forces
Source: Bipolar Disord. 2026 Jul 1;28(5):e70109. doi: 10.1111/bdi.70109 (PMC13324234; doi:10.1111/bdi.70109)
Supplement: Supplementary file 5 — Appendix S5, Table S1: Study characteristics of all studies on biofluid biomarkers in relation to cognitive function in individuals with bipolar disorder (n = 60). Appendix S5, Table S2: Risk of bias assessment for all of the studies included in this review (n = 60). [file BDI-28-0-s004.zip › bdi70109-sup-0006-TableS2-S6@SUPPLEMENTAL DATA FILE 6 - Table S2. Risk of Bias Assessment v3.docx]

**SUPPLEMENTAL DATA

TABLE S2. Risk of bias assessment for all of the studies included in this review (n=60)***

| **Study** | **1. Were the criteria for inclusion in the sample  (bipolar disorder) clearly defined?** | **2. Were the study subjects and the setting described in detail?** | **3. Was the exposure (biofluid biomarker) measured in a valid and reliable way?** | **4. Were objective, standard criteria used for measurement of the condition (bipolar disorder)?** | ***5. Were confounding factors identified?*** | **6. Were strategies to deal with confounding factors stated?** | **7. Were the outcomes (cognition)measured in a valid and reliable way?** | **8. Was appropriate statistical analysis used?** | **9. Sample size  (of the BD sample)** | **Overall Quality Appraisal** (GOOD/ FAIR/ POOR) |
| --- | --- | --- | --- | --- | --- | --- | --- | --- | --- | --- |
| **Aydemir et al. (2014)**  *Revista Brasileira de Psiquiatria/* *Brazilian Journal of Psychiatry* | Yes | Yes | No  mood scales & NPA after blood draw, probably on same day but not clear | Yes  SCID-CV | N/A | Yes  correction for  -duration of illness  -number of  episodes | No  multiple NP tests, multiple domains  no standardization or use of normative data  cognitive data not compared between BD & HC | Yes  Partial correlation with correction for two confounders | No  51 BD | **POOR** |
| **Barbosa et al. (2018)**  *Journal of Psychiatric Research* | Yes | Yes | Yes  blood draw on same day as clinical assessments | Yes  MINI | N/A | Yes  correction for MMSE and years of study  (maybe also duration of disease?) | Yes  global cognitive performance scale based on 8 tests  standardized Z-scores based on the entire sample, no normative data. Comparison of cognitive data between BD & HC. | Yes  multiple regression | No  20 BD | **FAIR** |
| **Bell et al. (1990)**  *Journal of Geriatric Psychiatry and Neurology* | No  clearly defined sample but with risk of bias. All patients that received a cognitive assessment (so bias in the sample, not all admitted patients received a cognitive assessment). Patients with very low vit B12 and folate were excluded, but study is focused on B12 and folate? | No  no info on duration of illness or medication use | No  blood draw at admission, NPA somewhere during admission | No  “DSM-III-R discharge diagnosis of psychotic depression” | N/A | No | No  single test scores as outcomes  No standardization or normative scores. No comparison of cognitive data with HC | No  correlation with correction for multiple comparisons | No  very small sample size: only 8 BD patients! | **POOR** |
| **Chen et al. (2019)**  *International Journal of Geriatric Psychiatry* | Yes | Yes | No  good protocol for blood draw, but unclear if blood draw and NPA were on same day | Yes  SCID | N/A | Yes  -age -gender - years of education  -duration of illness -10‐year Framingham cardiovascular risk scores -homocysteine -triiodothyronine | No  multiple tests, but single test scores as outcomes.  No standardization or normative scores. No comparison of cognitive data with HC. | No  main analysis on EOBD/ LOBD and cognition, biomarkers as covariates in final multiple regression model | No  95 BD | **POOR** |
| **Chen et al. (2022)** *The British Journal of Psychiatry Open (BJPsych Open)* | Yes | No  no info on duration of illness | Yes  The patients underwent fasting blood sampling in the early morning  on the same day of cognitive assessment. | No  “in accordance with DMS-5” | N/A | Yes  Model 1: age, gender, vitamin D level, NfL level and interaction of vitamin D and NfLs.  Model 2: age, gender, defined daily dose of total psychotropic medications, years of education, number  hospital admissions, level of physical activity determined with the International Physical Activity Questionnaire, vitamin D level, NfL level and interaction of vitamin D and NfLs. | Yes  BAC-A,  8 tests, 1 for each domain, also composite score.  Comparison  with norm references. No comparison of cognitive data with HC. | Yes  general linear models with correction for confounders | Yes  100 BD | **FAIR** |
| **Chou et al. (2012)** *Journal of Affective Disorders* | Yes | Yes | Yes  patients were evaluated bi-weekly | No  DSM-IV | N/A | Yes  Age, gender, education | Yes  Three domains, 2 tests per domain (but many subscores per test)  unclear if use of normative data (a reference to normative data is included) comparison of cognitive data to HC | Yes  Partial correlation with correction | No  23 BD | **FAIR** |
| **Civil Arslan et al. (2017)**  *Türk Psikiyatri Dergisi/* *Turkish Journal of Psychiatry* | Yes | No  no information on duration of illness | Yes  blood draw on same day as NPA | Yes  SCID | N/A | No | Yes  3 NP tests (different cognitive domains)  with multiple scores per test used as outcomes.  Turkish-language validated versions of tests, but unclear if use of normative data. Cognitive data is compared with HC (education matched) | No  -Pearson’s correlation -Spearman’s correlation  without correction | No  36 BD | **POOR** |
| **Dickerson et al. (2004)**  *Biological Psychiatry*  **sample overlap with Dickerson et al. (2006, 2013)** | Yes | Yes | Yes  “Each participant had a blood sample drawn at the time of the  study visit” | Yes  SCID | N/A | Yes  *final MANCOVA model: -years of education -PANSS Negative Symptom  score -current treatment with an  antipsychotic | Yes  very good: multiple tests per domain, multiple domains  use of normative data  cognitive data not compared to HC | Yes  MANOVA with correction for multiple comparisons | Yes  117 BD | **GOOD** |
| **Dickerson et al. (2006)**  *Bipolar Disorders*  **sample overlap with Dickerson et al. (2004, 2013)** | Yes | Yes | Yes  blood draw at the time of the cognitive  testing | Yes  SCID | N/A | Yes  *MANCOVA many confounders tested  *multinomial regression: BPRS, education level | Yes  multiple tests, multiple domains,  use of normative data (RBANS, age-adjusted standard  score with a mean of approximately 100 and a  standard deviation of approximately 15)  cognitive data compared to HC | Yes  MANOVA with correction for multiple comparisons | Yes  107 BD | **GOOD** |
| **Dickerson et al. (2013)**  *Journal of Affective*  *Disorders*  **sample overlap with Dickerson et al. (2004)** | Yes | No  no info on duration of illness | Yes  “Each participant had a blood sample drawn at the time of the  study visit” | Yes  SCID | N/A | Yes  -age -gender -race -maternal education -cigarettesmoking status -BMI -Ham-D -YMRS -seropositivity HSV-1 | Yes  multiple tests, multiple domains  Use of normative data.  Cognitive data compared to HC | Yes  logistic and linear regression | Yes  107 BD | **FAIR** |
| **Dickerson et al. (2014)** | Yes | Yes | Yes  “A blood sample was obtained at the study visit.” | Yes  SCID | N/A | Yes  age, sex, race, maternal education, and participant  education  logistic regression: also seropositivity HSV-1 IgG and Toxo IgG | Yes  Multiple tests, multiple domains  Use of normative data  Cognitive data compared to HC | Yes  logistic and linear regression | Yes  347 BD | **GOOD** |
| **Dittmann et al. (2007)**  *Bipolar Disorders* **Sample overlap with Dittmann et al. 2008** | Yes | Yes | No  not clearly stated if blood draw and NPA on same day | Yes  SCID | N/A | Yes  linear regression controlled for confounders: -age -gender -nr.  of previous episodes -HAM-D -nr. of  psychotropic medications | Yes  multiple tests, multiple domains  Use of normative data. Cognitive data compared to HC | Yes  linear regression | No  55 BD | **FAIR** |
| **Dittmann et al. (2008)**  *Journal of Clinical Psychiatry* **Sample overlap with Dittmann et al. 2007** | Yes | Yes | Yes  Blood was collected directly after NPA | Yes  SCID | N/A | Yes  Stepwise hierarchical regression controlling for confounders | Yes  multiple tests, multiple domains  Use of normative data. Cognitive data compared to HC | Yes  hierarchical regression | No  74 BD | **FAIR** |
| **Doganavsargil-Baysal et al. (2013)**  *Türk Psikiyatri Dergisi/* *Turkish Journal of Psychiatry* | Yes | No  no info on age, sex, or years of education | Yes  blood was drawn  following 12 hours of fasting on the day that neuropsychological  tests were administered | Yes  SCID | N/A | No  no correction for confounding | Yes  five tests, but single raw test scores were used as outcome.  No standardization or use of normative data. Cognitive data compared to HC. | No  correlations without correction | No  60 BD | **POOR** |
| **Garés-Caballer et al. (2022)** *Frontiers in Neurology* prospective, 1-year follow-up cohort study | Yes | Yes | No  Several biomarkers, clinical, sociodemographic data,  neurocognitive performance, and social functioning data were  collected at baseline (TB) and after 1 year (TY).  unclear if on same day! | No  The diagnoses of SZ, BD, and MDD were established following  the criteria of the Diagnostic and Statistical Manual of Mental  Disorders, Fifth Edition. | N/A | Yes  correction for confounders:  first univariate analyses, then multivariate with those biomarkers with most statistical significance. | Yes  Many tests and domains  standardization into Z scores based on HC data. | Yes  linear regression analysis with a predictive model (biomarkers at baseline, executive function at 1-year FU)  No more than  five variables were included in each model, thus ensuring the correct performance of the analysis. | No  42 BD at baseline, 29 at one year follow-up | **POOR** |
| **Gerber et al. (2012)**  *Progress in Neuro-Psychopharmacology & Biological Psychiatry* | Yes | Yes | No  Some protocol is reported but not which antibodies exactly. Also not clear if blood draw was on same day as neuropsychological testing. | Yes  SCID | N/A | No  Unclear? “The association of HSV-1 antibodies with  cognitive impairment proved to be independent of clinical and  demographic determinants” However, separate Pearson correlations between clinical and demographic determinants and cognitive outcomes were performed. | Yes  multiple tests, multiple domains  use of normative data  Comparison of cognitive data to HC.   In text, outcome ‘neuropsychological impairment’ is used, but this is not defined! | No  ANOVA Covariance analyses  Stepwise discriminant function analysis, but unclear if correction was performed and which analysis was performed when. | No  30 BD | **POOR** |
| **Hebbrecht et al. (2022)**  *Neuropsychobiology* longitudinal: baseline, 4 months,  8 months | Yes | No  no data on years of education  unclear gender distribution in table | Yes  “Every test day included the same  clinical, cognitive, and laboratory assessment” | Yes  MINI | N/A | Yes  correction for several confounders: -moment -significant group ×  moment interactions  -sex -age -smoking -BMI -years of education | Yes  multiple tests  tests were ‘standardized’, unclear how.  Cognitive data were compared to HC. | No  Linear mixed model with subject ID as random group | No  67 BD | **POOR** |
| **Hidese et al. (2023)**  *Neuropsychopharmacology Reports* | Yes | Yes | No  Detailed protocols, but unclear if administered on same day | Yes  MINI | N/A | Yes  Bonferroni correction and correction for: age,  sex, BMI, education level, current smoking status, and psychotropic  medication use | Yes  Two domains, many tests  No standardization with Z-scores.  Cognitive data were compared to HC | Yes  Pearson's partial correlation coefficient | Yes  115 BD | **FAIR** |
| **Huang et al. (2021)**  *Journal of Psychiatric Research* **sample overlap with Huang 2022?** | Yes | No  no info on illness duration | No  did not collect serum  samples of all participants at the same time of the day. unclear if on same day as cognitive assessments | No  diagnosed  with bipolar I disorder based on the Diagnostic and Statistical Manual of  Mental Disorders, Fifth Edition (DSM-5) (only MINI for healthy controls) | N/A | Yes  controlled for:  age, sex, education, history of suicide attempts, total MADRS  score, total YMRS score  + adjusted for go-no go task or 2-back task? | No  only two NP tests.  No standardization or use of normative data.  Cognitive data compared to HC. | No  Linear regression,  main analyses are SI on biomarkers as outcomes, adjusted for go-no go task or 2-back task | No  77 BD | **POOR** |
| **Huang et al. (2022)** *Psychiatry and Clinical Neurosciences* **sample overlap with Huang 2021?** | Yes | Yes | Yes  The blood sampling for the measurement of cytokines and clinical/  cognitive function assessments were conducted on the same day. | Yes  MINI | N/A | Yes  correction for:  age, sex, education,  BMI, YMRS total scores, MADRS total scores, psychotropic medication  use, and diagnostic group | Yes  Multiple domains, multiple tests  No standardization or use of normative data.  Cognitive data compared to HC. | Yes  GLM using the complete group (BD, MDD, HC), but including interaction for diagnostic groups | No  70 BD | **FAIR** |
| **Hui et al. (2019)**  *International Journal of Bipolar Disorders* | Yes | Yes | No  clear protocol for blood draw but unclear if on same day as cognitive assessments | Yes  SCID | N/A | Yes  correction for: -age -gender -education -BMI  -age of onset -duration of illness -nr. of episodes -nr. of hospitalizations -medication dosage -clinical sypmtoms | Yes  RBANS: multiple tests RBANS is ‘standardized’, unclear how.  Domain score controlled for gender, age, education, BMI and serum HDL levels.  Cognitive data was compared to HC. | Yes  stepwise multiple regression | No  37 BD | **FAIR** |
| **Jakobsson et al. (2013)**  *Neuropsychopharmacology* | Yes | No  years of education and illness duration not stated | No  “CSF sampling (lumbar puncture) was performed when the  participants were in a stable euthymic mood.”  unclear if on same day as cognitive assessments | Yes  SCID and other sources of data. Final diagnosis based on consensus panel of psychiatrists | N/A | No  no confounders | Yes  WAIS 4 cognitive domains + 1 NP test, not standardized but raw tests scores.  Cognitive data compared to HC. | No  spearman’s correlations with correction for multiple testing (False Discovery Rate) | Yes  139 BD | **POOR** |
| **Jonsson et al. (2022)**  *Brain and Behavior* | Yes | No  years of education and duration of illness not stated | Yes  Blood sampling was performed in the morning after an overnight fast  and in conjunction with the collection of the clinical data. | Yes  The baseline clinical diagnostic  instrument for BD was the Affective Disorder Evaluation (ADE)  (Sachs et al., 2003). The diagnostic assessments  were based on all available sources of information, including medical  records and interviews with family members when feasible. A consensus  panel of experienced board-certified psychiatrists specialized  in BD arrived at “best estimate” diagnoses. | N/A | Yes  adjusted for IQ | Yes  3 NP tests, but 52 subtests in total  “we used scaled scores (adjusted for age and sex) from all conditions across three individual tests in D-KEFS” | No  partial correlation | Yes  121 BD | **FAIR** |
| **King et al. (2019)**  *Frontiers in Psychiatry* | Yes | No  age, years of education, illness duration not clear | No  “blood samples” not clear if plasma or serum.  Blood sampling, behavioral  assessments and neurocognitive testing were conducted on the  same day as the brain scan, and neuropsychiatric assessments  were carried out on Day 1 if the session was split into two. | No  not stated how bipolar diagnosis was determined.  Substance abuse/dependence within the past 6 weeks was determined  by structured clinical interview for DSM-IV. | N/A | No  only correction for multiple comparisons (False Discovery Rate) | Yes  4 NP tests, 3 domains.  No standardization or use of normative data.  Cognitive data compared to HC. | No  Pearson product- moment correlations | No  15 BD (analysis of blood in 13 BD) | **POOR** |
| **Knöchel et al. (2017)**  *European Archives of Psychiatry and Clinical Neuroscience* | Yes | Yes | No  unclear if plasma or serum, both terms are used throughout the article | Yes  SCID | N/A | No  correlations only with Bonferroni correction, no adjustment for confounders | No  only 1 NP test (and IQ score) | No  Bivariate correlations | No  25 BD | **POOR** |
| **Lee et al. (2017)** *Progress in Neuro-Psychopharmacology & Biological Psychiatry* | Yes | Yes | Yes  BACA was assessed right after blood draw | Yes  Diagnosis by senior psychiatrist according to DSM-IV-TR + structured interview: Chinese Version of the Modified Schedule of Affective Disorder and Schizophrenia-Life Time (SADS-L) | N/A | Yes  correction for age, gender, years of education  (diagnosis of BP-II) | Yes  multiple tests, multiple domains,  cognitive data was normalized using the HC data | No  main analyses in BD+HC group, then post-hoc for BD-II and BD-II short | No  32 BD | **FAIR** |
| **Lee et al. (2018)**  *Neuropsychiatric Disease and Treatment*  RCT (open label valproate) | Yes | Yes | Yes  BACA was assessed right after blood draw | Yes  Diagnosis by senior psychiatrist according to DSM-IV-TR + structured interview: Chinese Version of the Modified Schedule of Affective Disorder and Schizophrenia-Life Time (SADS-L) | N/A | No  at baseline only Pearson’s correlations without correction | Yes  many domains and NP tests  Cognitive measures:  compared with the Mandarin-speaking norm, which was already controlled for age and gender for statistical analysis. | No  at baseline only Pearson’s correlations without correction | No  32 BD | **POOR** |
| **Li et al. (2015)**  *Psychoneuroendocrinology* | Yes | Yes | No  unclear if blood draw and cognitive assessment was on same day | Yes  MINI | N/A | No  no correction | No  1 NP test + MoCA + MMSE  Cognitive data compared to HC. | No  Pearson’s correlation  data not shown for analyses with outcome WCST! | No  27 BD | **POOR** |
| **Liou et al. (2023)** *Psychoneuroendocrinology* | Yes  but not completely representative of BD population (all participants received valproate) | No  duration of illness not measured | No  unclear if blood draw and cognitive assessment was on same day | Yes  structured  interview in the Chinese Version of the Modified Schedule of Affective  Disorder and Schizophrenia - Lifetime (SADS-L) | N/A | Yes  corrected for age, sex, education, HDRS, YMRS | Yes  3 NP tests Composite scores were calculated for eight “standardized”  domain scores  No use of normative data. Cognitive data compared to HC. | Yes  linear regression analysis | Yes  641+ 150 BD | **FAIR** |
| **Lotrich et al. (2014)** *International Journal of Geriatric Psychiatry* | Yes | No  duration of illness and medication use not reported | No  unclear if blood draw and cognitive assessment was on same day | Yes  SCID | N/A | No  In separate analyses in the BD+HC group: covarying for BDNF, BMI, IL-6, BD diagnosis BD diagnosis.  Unclear if the model that included BD diagnosis also included other covariates. | Yes  Four factor analysis-derived z-scores for cognitive domains and a global z-score, based on 21 neuropsychological tests. | Yes  multiple linear regressions | No  21 BD | **POOR** |
| **Mansur et al. (2020)**  *Psychoneuroendocrinology* RCT (infliximab) | Yes | No  Duration of illness not stated | Yes  in the same week (week 0)  (but unclear if blood draw and cognitive assessment was on same day) | Yes  MINI | N/A | Yes  controlled for age, gender, BMI, use tobacco and MADRS score | No  2 tests (2 domains)   No use of normative data, no comparison of cognitive data between BD & (HC. | Yes  GEE | No  60 BD (at baseline RCT) | **POOR** |
| **Millett et al. (2020)**  *Brain, Behavior, and Immunity* | Yes | Yes | No  unclear if blood draw and cognitive measure were on same day | Yes  SCID | N/A | Yes  controlled for  sex, age, education, race, IQ | Yes  3 tests  All neurocognitive scores from the MCCB  were standardized on a T scale score (mean = 50, SD = 10), and age  and sex adjusted using published MCCB normative data. | Yes  partial correlations (controlled for age and sex),  SEM & goodness of fit | Yes  219 BD | **FAIR** |
| **Millett et al. (2021)** *Molecular Psychiatry* | Yes | Yes | No    unclear if blood draw and cognitive assessment was on same day | Yes  SCID-V | N/A | Yes  controlled for age, sex and education | Yes  multiple tests, multiple domains  All scores were standardized, and age and sex  adjusted using MCCB normative data. | Yes  Partial correlations controlled for confounders | Yes  222 BD | **FAIR** |
| **Miskowiak et al. (2023)** *Journal of Psychiatric Research*  Longitudinal study | Yes | Yes | Yes  The clinical  assessments of mood, cognitive function, and the CSF and urine sampling  from the participants were done on the same or over two consecutive  days. | Yes  The clinical diagnosis (patients) or lack thereof (HC) was evaluated  using the Schedules for Clinical Assessment in Neuropsychiatry  interview (Wing et al., 1990) conducted by specialist in psychiatry | N/A | Yes  fixed effects: 1) age, sex 2) 1+ years of education, smoking, alcohol | Yes  many domains and many tests  The cognitive scores seem standardized (around 0) but unclear in the text | Yes  Linear mixed model | No  60 BD | **FAIR** |
| **Mora et al. (2019)**  *European Psychiatry* | Yes | Yes | No  Blood at beginning hospitalization, cognition prior to discharge | Yes  SCID | N/A | Yes  Linear regression with age, IQ, BMI, neurological signs as predictors | Yes  6 domains, different tests  comparison with HC | Yes  Linear regression | No  57 BD | **FAIR** |
| **Na et al. (2020)**  *Iran Red Crescent Medical Journal* | Yes | No  unclear if patients used medication | Yes  8.00 AM at day of enrollment | No  criteria DSM evaluated by  “two physicians” | N/A | No  no confounders | No  2 tests, different scores | No  Pearson and Spearman related analyses | Yes  100 BD | **POOR** |
| **Omileke et al. (2019)**  *Neuropsychopharmacology Reports* | Yes | No  unclear if patients used medication | No  no fasting and unclear if blood draw and cognitive assessment was on same day | Yes  MINI | N/A | No  no confounders | Yes  BACS  compared with HC | No  Spearman correlations | No  26 BD | **POOR** |
| **Osher et al. (2008)**  *Journal of Affective Disorders* | Yes | No  years of education not stated | Yes  after cognitive testing, blood was obtained | No  chart + clinical interview | N/A | Yes  Covariate: age | Yes  5 domains, different tests  with comparison group (normal subjects) | Yes  ANCOVA | No  57 BD | **POOR** |
| **Paribello et al. (2023)** *Brain Sciences* | Yes | Yes | No  unclear if blood draw and cognitive assessment was on same day | Yes  SCID-V | N/A | Yes  gender as  a cofactor and total time of illness at recruitment (months) as covariate | Yes  many NP tests, many domains  Corrections according to the normative Italian population  have been applied to the gathered raw data for the BAC-A performances | Yes  linear regression | No  50 BD of which 45 BD with cognitive data for analysis | **FAIR** |
| **Permoda-Osip et al. (2014)**  *Psychiatria Polska* | No  “all patients were hospitilized, no illnessses that may influences] cognition were diagnosed” | No  years of education not stated | No  unclear if blood draw and cognitive assessment was on same day | No  no information on assessment diagnosis | N/A | No  no confounders | No  3 tests  no controls or norm corrections | No  Pearson correlations | Yes  116 BD | **POOR** |
| **Peters et al. (2022)** *Journal of Affective Disorders* **Sample is a subset of the sample of Millett et al. (2021)** | Yes | Yes | No  unclear if blood draw and cognitive assessment was on same day | Yes  SCID-5 | N/A | Yes  multivariate: 1st step: HDRS, YMRS 2nd step: age, sex, race, education, smoking, lifetime psychosis, no. psychotropic medications, BD illness duration, no. psychiatric hospitalizations, no. mood episodes | No  1 test affective Go/no-go task with 9 subitems | Yes  multivariate: stepwise regression with log transformed CRP | Yes  119 BD | **FAIR** |
| **Platzer et al. (2017)**  *Psychoneuroendocrinology* | Yes | No  duration of illness and use of medication not stated | No  unclear if blood draw and cognitive assessment was on same day | Yes  SCID | N/A | Yes  Covariates: obesity, age, level education | Yes  4 tests | Yes  partial correlation | No  68 BD | **POOR** |
| **Poletti et al. (2021)**  *Journal of Psychiatric Research* | Yes | Yes | No  unclear if blood draw and cognitive assessment was on same day | No  no information on assessment diagnosis | N/A | Yes  covariates: age, sex, education, frequencie mood episodes, BMI, imipramine/chlorpromazine doses, lithium, HDRS | Yes  BACS (broad battery, different domains)  all data were normalized (i.e. min-max normalization). | Yes  GLM and logistic regression | No  76 BD | **POOR** |
| **Reininghaus et al. (2016)**  *Bipolar Disorders* | Yes | Yes | No  8.00-9.00 after fasting.  Unclear if blood draw and cognitive assessment was on same day | Yes  SCID | N/A | Yes  correction for age, smoking, BMI | Yes  4 tests  comparison with HC | Yes  partial correlation | Yes  112 BD | **FAIR** |
| **Rolstad et al. (2015a)**  *PloS ONE* | Yes | No  No info on duration of illness | No  both when euthymic,  unclear if it is the same day as testing | Yes  ADE (Affective Disorder Evaluation) | N/A | Yes  covariates: age, sex, bipolar subtype, CGI, MADTS, YMRS, mood stabilizer, antidepressant, antipsychotics, benzodiapines, anxiolytics | Yes  5 domains, different tests  Z scores based on HC | Yes  linear regression | No  82 BD | **POOR** |
| **Rolstad et al. (2015b)**  *European Neuropsychopharmacology* | Yes | No  No info on duration of illness | No  9.00-10.00  after night fasting,  unclear if it is the same day ss testing | Yes  ADE | N/A | Yes  covariates: age, bipolar subtype, CGI, MADS, YRS, GAF, use of mood stabilzers, antidepressants, antipsychotics, benzodiazepines | Yes  5 domains different tests .  The individual test scores were z-transformed (M=0; SD=1) on the  basis of controls´ performance and then combined into cognitive  domains guided by their common measurement properties according to reference literature. | Yes  linear regression | No  78 BD | **POOR** |
| **Rubin et al. (2014)**  *Schizophrenia Bulletin* | Yes | Yes | No  in morning when possible (89%)  unclear if blood draw and cognitive assessment was on same day | Yes  SCID | N/A | Yes  adjusted for age, race, sex | Yes  emotion processing and total BACS  comparison with HC | Yes  linear regression | No  75 BD psychotic | **FAIR** |
| **Sağlam Aykut et al. (2018)**  *Nordic Journal of Psychiatry* | Yes | Yes | Yes  Blood draw on day of cognitive tests | Yes  SCID | N/A | No  no confounders | Yes  3 tests  comparison with HC | No  Pearson or Spearman correlations | No  28 BD | **FAIR** |
| **Salvi et al. (2020)**  *Journal of Affective Disorders* | Yes | Yes | No  Patients were then  asked to undergo assessment by psychiatrist and cognitive testing  within one month from the blood sampling. | Yes  SCID-I | N/A | Yes  covariates: age and site as random factor | No  only 2 NP tests (but more subtests) | Yes  general linear model | Yes  100 BD | **FAIR** |
| **Sanchez-Autet et al. (2018)**  *Journal of Affective Disorders* | Yes | Yes | Yes  8.00-11.00 within 4 weeks, 8 hours fasting | Yes  SCID | N/A | Yes  co-variates: age, years education, duration illness, number hospital admissions, HDRS, tobacco consumption and BMI | Yes  SCIP: 5 cognitive domains  raw scores transformed in impairment groups (unclear if only compared to patients or controls) | Yes  partial correlations  and hierarchical multiple  regression analysis | Yes  224 BD | **GOOD** |
| **Strawbridge et al. (2021)** *The British Journal of Psychiatry Open (BJPsych Open)* | Yes | No  no data on years of education or duration of illness | Yes  “The data examined in this study was then provided in  a single session, before participants were randomised to receive a  cognitive remediation intervention or continue treatment as usual.” | Yes  MINI | N/A | Yes  non-biological confounders were selected from the spearman’s correlation and t-tests if p<0.01. These differed per model, but included: number of medications, smoking, bipolar type, physical illness, FAST, gender, age, number of episodes, CTQ, HRQOL | Yes  multiple tests and multiple domains  For each test, the raw score was transformed  into standardized normative scores | Yes  logistic regression | No  44 BD | **FAIR** |
| **Tanaka et al. (2017)**  *Neuroscience Research* | No  “newly enrolled outpatients recruited between august 2008 and december 2014, none in first lifetime episode” | No  lacks info on duration of illness | No  unclear if blood draw and cognitive assessment was on same day | Yes  “structured interview” | N/A | Yes  controlling for age & smoking | Yes  BACS average  compared to HC | Yes  linear regression | No  32 BD | **POOR** |
| **Thompson et al. (2005)**  *British Journal of Psychiatry* | Yes | Yes | Yes  8.00, 12.00, 16.00 and 20.00 day before testing | Yes  SCID | N/A | No  no confounders | Yes  4 domains, different tests  compared to control group | No  only Spearman correlation,  post hoc multivariate statistics | No  63 BD | **POOR** |
| **Tournikioti et al. (2021)**  *Neuropsychobiology* | Yes | Yes | No  8.00  fasting blood samples, unclear if on the same day as testing | Yes  MINI | N/A | Yes  controlling for gender, age, education & clinical parameters | Yes  3 tests, 2 domains  compared to HC | Yes  stepwise regression | No  60 BD | **FAIR** |
| **Tournikioti et al. (2022)**  *Psychiatry Research* | Yes | Yes | No  8.00  unclear if blood draw and cognitive assessment was on same day | Yes  MINI | N/A | Yes  controlled for gender education  illness duration  current use of mood stabilizers | No  2 tests (one per domain)  no control group | Yes  stepwise regression | No  60 BD | **POOR** |
| **Tozoglu et al. (2021)**  *Archives of Clinical Psychiatry* | Yes | Yes | No  9.00-12.00 after 12 hours fasting  unclear if blood draw and cognitive assessment was on same day | Yes  SCID | N/A | No  no covariates | Yes  5 tests, different domains  No use of normative data  Cognitive data compared with controls | No  only bivariate Pearson correlations | No  42 BD | **POOR** |
| **van der Werf-Eldering et al. (2012)**  *PLoS ONE*  longitudinal study (over the course of one day) | Yes | Yes | Yes  Patients were instructed to collect  saliva as soon as possible after the cognitive  testing day. The median time between the cognitive testing day  and saliva sampling was 6.0 days (25th–75th percentile, 2.0–11.5  days). | Yes  MINI | N/A | Yes  controlled for gender, education, IQ  (cognitive data was already age-adjusted) | Yes  multiple tests, multiple domains  cognitive data were turned into age-adjusted Z-scores based on data from HC | Yes  linear regression | No  65 BD | **FAIR** |
| **Van Rheenen et al. (2021)**  *Journal of Affective Disorders* | Yes | Yes  (10% stable) | Yes  end of study day, absence food 2-3 hours | Yes  MINI | N/A | Yes  controlling for age | No  2 tests (one domain) | Yes  linear regression, controlling for age with  Bonferroni correction | No  23 BD | **FAIR** |
| **Van Rheenen et al. (2023)** *Psychiatry Research* | Yes | Yes | No  unclear if blood draw and cognitive assessment was on same day | Yes  MINI | N/A | No  in model of global cognition (BD+HC): corrected for season of blood draw and WTAR  in BD only sample: no correction for confounders | Yes  Multiple tests, multiple domains  Raw MCCB test scores were converted to domain  t-scores that were age and gender corrected based on a normative  sample of 300 healthy controls | Yes  ANCOVAs | No  55 BD | **POOR** |
| **Zazula et al. (2022)**  *The World Journal of Biological Psychiatry* | Yes | Yes | No  in morning  unclear if blood draw and cognitive assessment was on same day | Yes  SCID | N/A | Yes  Controlled for gender, age, anxious, depression & manic symptoms | Yes  3 tests  compared to controls | Yes  multiple linear regression | No  31 BD euthymic | **FAIR** |

* The criteria were adapted from The Joanna Briggs Institute Critical Appraisal tools for use in JBI Systematic Reviews; Checklist for Analytical Cross Sectional Studies
<http://joannabriggs.org/research/critical-appraisal-tools.html>; https://jbi.global/sites/default/files/2019-05/JBI_Critical_Appraisal-Checklist_for_Analytical_Cross_Sectional_Studies2017_0.pdf
See document “**Supplemental Data – criteria for risk of bias assessment**” for the adapted criteria.
